# Pan-cancer analysis identifies *BIRC5* as a prognostic biomarker

**DOI:** 10.1186/s12885-022-09371-0

**Published:** 2022-03-25

**Authors:** Anna Fäldt Beding, Peter Larsson, Khalil Helou, Zakaria Einbeigi, Toshima Z. Parris

**Affiliations:** 1 Department of Oncology, Institute of Clinical Sciences, Sahlgrenska Center for Cancer Research, Sahlgrenska Academy, University of Gothenburg, Gothenburg, Sweden; 2 Department of Oncology, Southern Älvsborg Hospital, Borås, Sweden; 3 Department of Oncology, Sahlgrenska University Hospital, Gothenburg, Sweden

**Keywords:** Cancer, Pancancer, TCGA, *BIRC5*, Survivin, Gene expression, Amplification, Prognosis, Biomarker

## Abstract

**Background:**

The *BIRC5* gene encodes for the Survivin protein, which is a member of the inhibitor of apoptosis family. Survivin is found in humans during fetal development, but generally not in adult cells thereafter. Previous studies have shown that Survivin is abundant in most cancer cells, thereby making it a promising target for anti-cancer drugs and a potential prognostic tool.

**Methods:**

To assess genetic alterations and mutations in the *BIRC5* gene as well as *BIRC5* co-expression with other genes, genomic and transcriptomic data were downloaded via cBioPortal for approximately 9000 samples from The Cancer Genome Atlas (TCGA) representing 33 different cancer types and 11 pan-cancer organ systems, and validated using the ICGC Data Portal and COSMIC. TCGA *BIRC5* RNA sequencing data from 33 different cancer types and matching normal tissue samples for 16 cancer types were downloaded from Broad GDAC Firehose and validated using breast cancer microarray data from our previous work and data sets from the GENT2 web-based tool. Survival data were analyzed with multivariable Cox proportional hazards regression analysis and validated using KM plotter for breast-, ovarian-, lung- and gastric cancer.

**Results:**

Although genetic alterations in *BIRC5* were not common in cancer, *BIRC5* expression was significantly higher in cancer tissue compared to normal tissue in the 16 different cancer types. For 14/33 cancer types, higher *BIRC5* expression was linked to worse overall survival (OS, 4/14 after adjusting for both age and tumor grade and 10/14 after adjusting only for age). Interestingly, higher *BIRC5* expression was associated with better OS in lung squamous cell carcinoma and ovarian serous cystadenocarcinoma. Higher *BIRC5* expression was also linked to shorter progressive-free interval (PFI) for 14/33 cancer types (4/14 after adjusting for both age and tumor grade and 10/14 after adjusting only for age). External validation showed that high *BIRC5* expression was significantly associated with worse OS for breast-, lung-, and gastric cancer.

**Conclusions:**

Our findings suggest that *BIRC5* overexpression is associated with the initiation and progression of several cancer types, and thereby a promising prognostic biomarker.

**Supplementary Information:**

The online version contains supplementary material available at 10.1186/s12885-022-09371-0.

## Introduction

The *BIRC5* (Baculoviral IAP Repeat Containing 5) gene located on chromosome 17 (17q25.3) encodes for the Survivin protein, which is a member of the IAPs (inhibitor of apoptosis family) that is normally expressed in humans during fetal development and in adult proliferating cells [1]. Survivin is a small protein with different isoforms, the majority of which are related to inhibition of apoptosis and promotion of cell proliferation [2]. Research during the past 20 years has shown that Survivin is highly expressed in most cancer cells [3, 4]. Although attempts have been made to develop small molecules targeting Survivin, there is no treatment currently in therapeutic use [5]. Recently, a study identified an association between *BIRC5* expression and tumor-infiltrating lymphocytes (TILs) [6]. Moreover, we previously evaluated *BIRC5* in breast cancer subtypes, thereby demonstrating that high *BIRC5* expression is associated with worse prognosis in breast cancer patients [7].

Several studies have found that Survivin can be implicated in chemoresistance to platinum-based [8] or taxane-based [9] chemotherapy in ovarian cancer. In contrast, a previous study comparing Survivin expression in ovarian cancer patients (*n* = 435) treated with platinum/cyclophosphamide (PC) (*n* = 244) or taxane/platinum (TP) (*n* = 191) found that patients with high nuclear Survivin expression and an accumulation of TP53 in tumor cells that were treated with TP had a decreased risk of recurrence and death [10]. Furthermore, high nuclear Survivin expression and TP53 dysfunction had a higher likelihood of having high platinum sensitivity. A recent in vitro study on human cell lines of neuroendocrine tumors (NETs) showed increased *BIRC5* expression in irradiated cells, additionally *BIRC5* knockdown resulted in reduced cellular proliferation but not significantly increased radiosensitivity [11]. Kleinberg et al. [12] observed an association between high nuclear Survivin in tumor samples and improved progression-free survival (PFS) in chemotherapy-naïve patients. Expression analysis of the *BIRC* gene family in 30 patients with triple-negative breast cancer (TNBC) [13] identified higher gene expression of the *BIRC* gene family (including *BIRC5*) in patients (< 50 years old) with TNBC. In contrast, TNBCs with lymphovascular and fat tissue invasion had lower expression of *BIRC* genes. Although *BIRC5* had the highest average expression of the tested genes, high *BIRC5* expression had no significant association with tumor size. However, there was a significant difference in *BIRC5* expression when comparing patients with no nodal metastasis (N0) with patients with micrometastases up to 1–3 axillary metastases and when comparing N0 with patients with 10 or more nodal metastases. There was also an association between histopathological grade in breast tumors and *BIRC5* expression [13].

Copy number gains of three *BIRC* genes (*BIRC2*, *BIRC3*, and *BIRC5*) were identified in melanoma [14], while miR-195-5p/− 218-5p, and not genetic/epigenetic aberrations, was correlated in high *BIRC5* levels in gastric cancer [15]. In the present study, we used publicly available -omics (genomics, transcriptomics) and survival data to examine *BIRC5* genetic alterations and altered expression in 33 cancer types in relation to prognosis.

## Methods

### Data collection

#### cBioPortal for Cancer Genomics repository

The cBioPortal for Cancer Genomics repository [16–18] was first used to analyze multi-omics data from The Cancer Genome Atlas (TCGA Pancancer) [19] for the *BIRC5* gene. Genomic and transcriptomic data from approximately 9000 samples representing 33 different cancer types and 11 pan-organ systems were analyzed (Table 1). Esophageal squamous and adenocarcinoma were combined into esophageal carcinoma. Colon and rectal carcinoma were combined into colorectal carcinoma in available genomic data from cBioPortal, resulting in 32 different tumor groups. First, *BIRC5* gene alteration frequency was determined on the DNA level for the different cancer studies. Genetic alterations were subsequently divided into mutation, fusion, amplification, deep deletion, multiple alterations for 8812 samples from 32 different cancer types. From the same platform, we downloaded DNA amplification data for *BIRC5* in the different cancer types.

**Table 1:** Genomic and transcriptomic datasets

Cancer type and pan-organ system	Cohort	Number of samples				
		Genetic alterations^a^	RNA-seq^a^		Co-expression^a^	KM plotter
			Cancer tissue	Normal tissue		
**Central nervous system**						
Glioblastoma multiforme	GBM	592	166	5	145	
Brain lower grade carcinoma	LGG	514	530	0	511	
**Endocrine**						
Adrenocortical carcinoma	ACC	91	79	0	78	
Thyroid carcinoma	THCA	500	496	58	480	
**Gastrointestinal**						
Cholangiocarcinoma	CHOL	36	36	9	36	
Colon adenocarcinoma	COAD^b^	﻿594	191	0	524	
Rectum adenocarcinoma	READ		72	0		
Esophageal Adenocarcinoma	ESCA^c^	182	185	11	181	
Esophageal squamous carcinoma						
Liver hepatocellular carcinoma	LIHC	372	147	50	348	
Pancreatic adenocarcinoma	PAAD	184	56	0	168	
Stomach adenocarcinoma	STAD^f^	440	415	35	407	875
**Gynecologic**						
Breast invasive carcinoma	BRCA	1084	1026	108	994	1879
Cervical squamous cell carcinoma	CESC^d^	297	159	0	275	
Ovarian serous cystadenocarcinoma	OV	584	265	0	201	1656
Uterine corpus endometrial carcinoma	UCEC	529	369	0	507	
**Head and neck**						
Head and neck squamous cell carcinoma	HNSC	523	425	42	488	
**Hematologic and lymphatic malignancies**						
Diffuse Large B-cell Lymphoma	DLBC	41	48	0	37	
Acute myeloid leukemia	LAML	200	173	0	165	
Thymoma	THYM	123	120	0	119	
**Melanocytic**						
Skin cutaneous melanoma	SKCM	444	472	0	363	
Uveal melanoma	UVM	80	80	0	80	
**Neural crest-derived**						
Pheochromocytoma and paraganglioma	PCPG	178	184	3	161	
**Soft tissue**						
Sarcoma	SARC	255	105	0	251	
Uterine carcinosarcoma	UCS	57	57	0	56	
**Thoracic**						
Lung adenocarcinoma	LUAD^e^	566	490	58	503	1925
Lung squamous cell carcinoma	LUSC^e^	487	482	50	466	
Mesothelioma	MESO	87	87	0	82	
**Urologic**						
Bladder urothelial carcinoma	BLCA	411	223	19	402	
Kidney chromophobe	KICH	65	66	25	65	
Kidney renal clear cell carcinoma	KIRC	511	507	72	352	
Kidney renal papillary cell carcinoma	KIRP	283	161	30	274	
Prostate adenocarcinoma	PRAD	494	498	52	488	
Testicular germ cell tumors	TGCT	145	156	0	144	
**Total**		8812	8526	627	9351	6335

^a^TCGA data, see Method

^b^In bioPortal COAD+READ was combined for our analysis

^c^In RNA-seq data esophageal adenocarcinoma and squamous carcinoma were combined. In analysis of RNA-seq data for gene co-expression there was only data for esophageal adenocarcinoma

^d^When analyzing gene co-expression using the RNA-seq data, cervical squamous cell carcinoma and endocervical adenocarcinoma were both included

^e^In KM-plotter both LUAD and LUSC were included in analysis

^f^Stomach cancer was in KM plotter denominated as gastric cancer

The cBioPortal repository was then used to identify genes that were co-expressed with *BIRC5* for 32 tumor types corresponding to 9351 samples (Table 1). Only data for esophageal adenocarcinoma (and not esophageal squamous carcinoma) were available for this analysis, while cervical squamous cell carcinoma and endocervical adenocarcinoma were both included under cervical squamous cell carcinoma (CESC). Spearman’s correlation was used to identify genes with mRNA expression that were significantly correlated with *BIRC5* mRNA expression. Pathway analysis was then performed using Reactome [20, 21] with *BIRC5* and the top 100 co-expressed genes for every tumor type.

#### Broad GDAC Firehose and UCSC Xena Browser

RNA sequencing (RNA-seq) data (UNC RNASeqV2 level 3 expression (normalized RSEM) for *BIRC5* expression were downloaded from Broad GDAC Firehose [22] for 8526 TCGA tumor samples corresponding to the 33 different cancer types, as well as matching normal tissue samples (*n* = 627) for 16 cancer types (*n* = 5507; Table 1). *Liu* et al. recently compiled genomic and clinical data for the TCGA dataset into a standardized version called the TCGA Pan-Cancer Clinical Data Resource (TCGA-CDR) [23]. Therefore, we downloaded survival and phenotype data for the TCGA dataset from UCSC Xena Browser [24, 25] and from National Cancer Institute – Genomic Data Commons [23, 26]. Although the survival data included four clinical end points, i.e. overall survival (OS), disease-specific survival (DSS), disease-free interval (DFI) and progression-free interval (PFI), OS (the time from diagnosis to death of any cause) and PFI (the time from diagnosis to new tumor event, e.g. progression of disease, local recurrence, distant metastasis, new primary tumors, or died with the cancer without a new tumor event) were used in the present study as these were deemed to be relatively accurate endpoints by Liu et al.

#### External validation

External validation of the results found using TCGA data was performed in four steps.

1. ***Genomics datasets:*** Cancer genomics datasets from the International Cancer Genome Consortium (ICGC) and Catalogue of Somatic Mutations in Cancer (COSMIC) genome version GRCh38 were analyzed to validate potential somatic mutations of ‘high mutation impact’ or ‘pathogenic’ in the *BIRC5* gene [27–30].
2. ***Transcriptomics datasets:*** Two RNA microarray datasets (Affymetrix HG-U133A and Affymetrix HG-U133 Plus 2) for *BIRC5* were retrieved from the GENT2 web-based tool for cancer and corresponding normal tissues [31, 32]. Cancer types with less than 10 normal and/or cancer samples were removed from the analysis (i.e. Affymetrix HG-U133A: adrenal gland, bladder, cartilage, larynx, muscle, pharynx, small intestine, soft tissue, tongue, urothelium; Affymetrix HG-U133 Plus 2: bone, eye, gall bladder, lymph node, muscle, pharynx, placenta, spleen, teeth, testis, vagina). After filtering, the Affymetrix HG-U133A dataset included 21 cancer types comprised of 16,539 cancer samples and 4283 normal samples, while the Affymetrix HG-133 Plus 2 dataset included 25 cancer types comprised of 35,523 cancer samples and 5063 normal samples.
3. ***Genomics and transcriptomics breast cancer dataset:*** DNA microarray, SNP genotyping, and RNA-seq data for breast cancer [33] were reevaluated from our previous work to identify DNA copy number alterations, exonic variants, and gene fusions. Additionally, survival analysis was performed using *BIRC5* expression and OS.
4. ***Survival analysis:*** Survival analysis for *BIRC5* gene expression and OS was performed using the KM plotter web-based tool [34] with RNA microarray data for breast- [35], ovarian- [36], lung- [37], and gastric cancer [38]. The following settings were selected in KM plotter: (1) *BIRC5* (Affymetrix probe 202094_at), (2) ‘auto select best cutoff’ to stratify the patient cohort, (3) OS endpoint, and (4) only ‘Jetset’ best probe set [39]. No cutoffs were made with regards to tumor subtype or treatment.

### Statistical analysis

Statistical analyses were performed using R/Bioconductor 3.12 (BiocManager 1.30.12) in RStudio (version 1.3.1073), where *p*-value < 0.05 was considered to be statistically significant. *BIRC5* expression in cancer samples was compared to expression in corresponding normal tissue. Tumor samples with no corresponding normal samples were removed from the analysis (Table 1). Boxplots were then generated with R packages ggpubr version 0.4.0 [40] and rstatix version 0.6.0 [41] using Wilcoxon test adjusted with Benjamini-Hochberg correction. DNA amplification data for *BIRC5* from TCGA Pancancer was matched with RNA sequencing data from Broad GDAC Firehose using Wilcoxon test to determine the effect of DNA amplification on *BIRC5* gene expression.

Multivariable Cox proportional hazards regression analysis was performed using R packages survival version 3.2–7 [42, 43], survminer version 0.4.9 [44], and Publish version 2020.12.23 [45]. Cox regression models were calculated using RNA sequencing data for *BIRC5* expression with the OS and PFI endpoints, adjusting for age and/or tumor grade (if available). Only age was available for 21/33 cancer types (ACC, BRCA, COAD, DLBC, GBM, KICH, KIRP, LAML, LUAD, LUSC, MESO, PCPG, PRAD, READ, SARC, SKCM, TGCT, THCA, THYM, UCS, UVM), while both age and tumor grade were available for 12/33 cancer types (BLCA, CESC, CHOL, ESCA, HNSC, KIRC, LGG, LIHC, OV, PAAD, STAD, UCEC). Forest plots were generated using the R package forestplot version 1.10 [46]. Due to missing data, LAML was excluded in the PFI analysis. For the external breast cancer dataset [33], multivariable Cox regression models adjusted using age and tumor grade were calculated using *BIRC5* expression and OS.

To identify clinicopathologic features that were associated with *BIRC5* expression, *BIRC5* expression was first categorized from RNA sequencing data as low *BIRC5* (lower than median *BIRC5* expression) and high *BIRC5* (higher than median *BIRC5* expression) by calculating the quantiles (0, 25, 50, 75, 100%) for *BIRC5* expression; median *BIRC5* expression (50%, quantile 2) was 0.4996274. Phenotype data were then retrieved for each cancer type from Xena Browser and matched with the RNA sequencing data in one file. Tableone script (version 0.13.0) in R was then used to identify clinicopathologic features associated with *BIRC5* expression. However, 9/33 cancer types (COAD, DLBC, GBM, LAML, OV, READ, SKCM, TGCT, UCS) could not be analyzed in tableone due to that they only had samples with high *BIRC5* expression.

## Results

### Genetic alterations in *BIRC5* are relatively uncommon in cancer

**Fig. 1:**
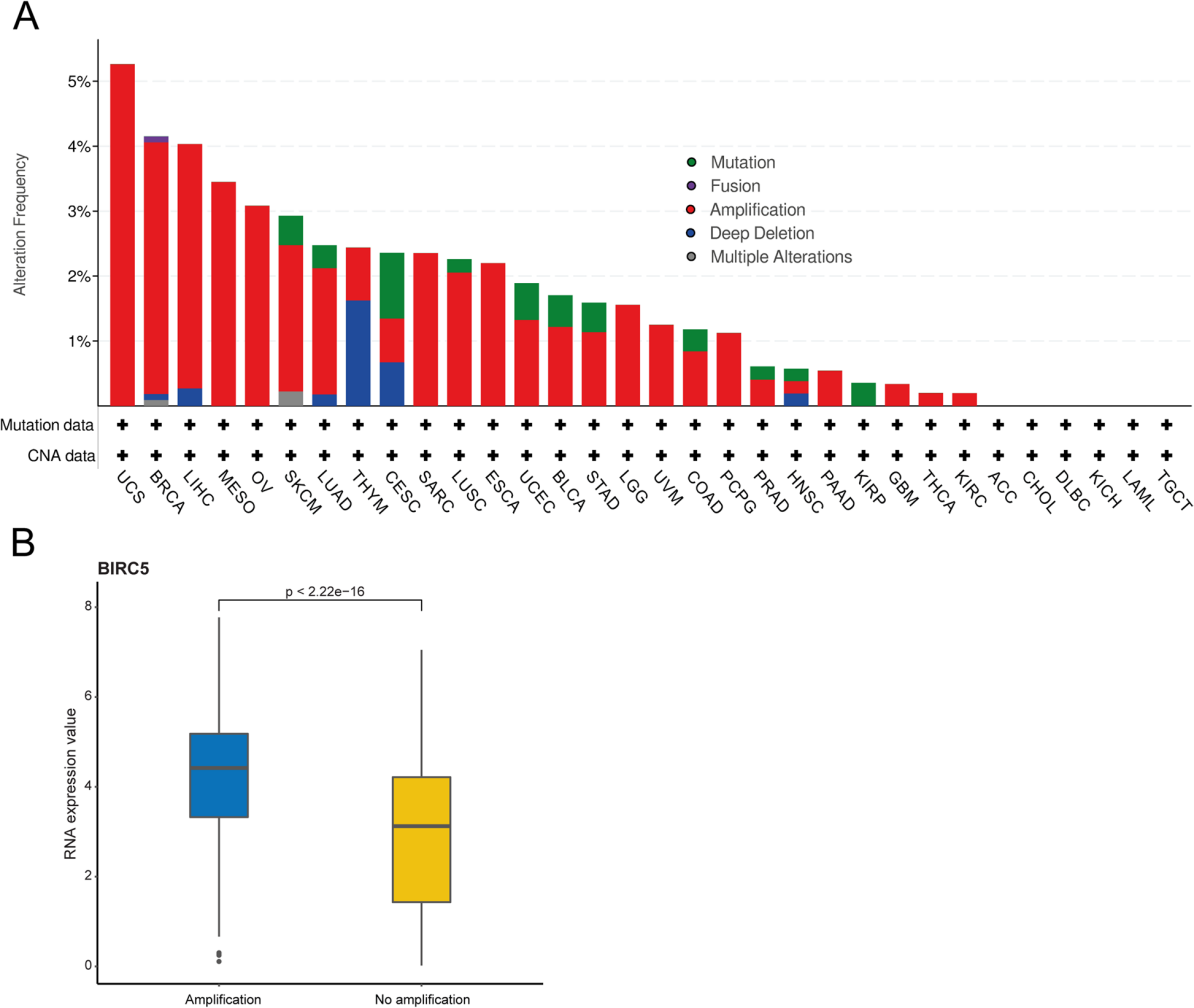
Distribution of genetic alterations in the *BIRC5* gene in 32 cancer types using the interactive web-based online tool cBioPortal (cbioportal.org). **A** Although only 196 of the 8812 cases (2%) had a gene alteration of any kind, DNA amplification was found to be most prevalent. Figure modified from cBioPortal [16, 17]. **B** Amplification of *BIRC5* in relation to mRNA expression levels of *BIRC5* (*P* < 2.2e-16)

To evaluate the prevalence of genetic alterations (i.e. inframe mutation, missense mutation, truncating mutation, fusion, amplification, and deep deletion) in the *BIRC5* gene, genomic profiling data from cBioPortal were analyzed for 8812 cancer samples representing 32 different cancer types. *BIRC5* was only found to be altered in 2% (*n* = 196) of cases, primarily DNA amplification (Fig. 1A). Uterine carcinosarcoma (UCS), invasive breast carcinoma (BRCA), liver hepatocellular carcinoma (LIHC), mesothelioma (MESO) and ovarian cancer (OV) had the highest alteration frequencies, i.e., 5.3, 4.2, 4.0, 3.5 and 3.1%, respectively. Deep deletions were more common in thymoma (THYM), but only 3/123 cases harbored genetic alterations in *BIRC5* (2 cases with deep deletions and 1 case with DNA amplification). No genetic alterations were seen in adrenocortical carcinoma (ACC), cholangiocarcinoma (CHOL), diffuse large B-cell lymphoma (DLBC), kidney chromophobe (KICH), acute myeloid leukemia (LAML) or testicular germ cell tumors (TGCT). RNA expression levels were found to be higher for tumor samples with higher amplification of *BIRC5* (*P* < 2.2e^− 16^; Fig. 1B).

To validate these findings, the ICGC Data Portal and COSMIC were used to identify somatic mutations of ‘high mutation impact’ or ‘pathogenic’ in the *BIRC5* gene. ICGC data showed that eight patients affected by different cancer types harbored eight different *BIRC5* mutations (Additional Table 1). Four of the eight cancer projects (BRCA, PRAD, SKCM, UCEC) were derived from TCGA data and the other four were from projects in China (colorectal cancer, COCA-CN; liver cancer, LICA-CN; nasopharyngeal cancer, NACA-CN) and Spain (chronic lymphocytic leukemia, CLLE-ES). For the TCGA data, high impact *BIRC5* mutations were classified as missense for SKCM and UCEC and stop gain for BRCA and PRAD, which were in line with the findings in cBioPortal, *BIRC5* mutations in the other datasets were classified as a frameshift mutation in COCA-CN, start loss mutation in LICA-CN, missense mutation in NACA-CN, and frameshift mutation in CLLE-ES. Furthermore, genome-wide screening data (array comparative genomic hybridization and SNP genotyping) were reevaluated from our previous work on breast cancer. DNA amplification in the *BIRC5* gene was found in 15/229 (0.066%) breast cancer samples. None of the samples were shown to harbor deep deletions, mutations or fusions. COSMIC data revealed 210 unique cancer samples with somatic mutations in the *BIRC5* gene, of which 33 were classified as pathogenic mutations (Additional Table 2). In total, 6 nonsense substitutions (breast, endometrium, hematopoietic and lymphoid, large intestine), 22 missense substitutions (breast, cervix, endometrium, esophagus, hematopoietic and lymphoid, kidney, large intestine, lung, prostate, skin, stomach, urinary tract), 3 synonymous substitutions (skin), and 1 unclassified mutation (head and neck) were identified. Eighteen of the 33 unique samples were derived from TCGA data.

### *BIRC5* levels are elevated in cancer compared to normal samples

**Fig. 2:**
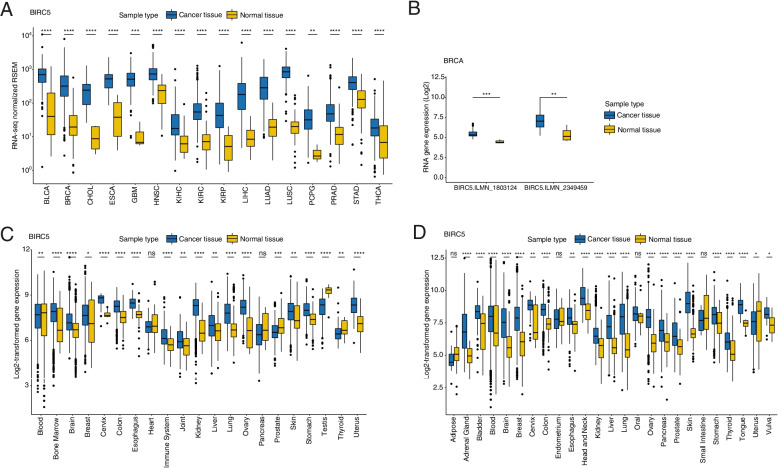
Box plots showing *BIRC5* expression in (**A**) 16 different TCGA cancer types, (**B**) breast cancer compared to corresponding normal tissue. *BIRC5* expression is significantly higher in all of the analyzed cancer types than normal tissue. **C** Affymetrix HG-U133A RNA microarray dataset with 21 cancer types, and (**D**) Affymetrix HG-U133 Plus 2 RNA microarray dataset comprised of 25 cancer types. *BIRC5* expression is significantly higher in the majority of the analyzed cancer types than normal tissue. The Wilcoxon test was used to calculate statistically significant (Benjamini-Hochberg adjusted *p*-values) differences in *BIRC5* expression between cancer and normal tissue. ns = not significant (*P* > 0.05); *P ≤ 0.05; **P ≤ 0.01; ***P ≤ 0.001; ****P ≤ 0.0001

To determine whether *BIRC5* expression patterns differ in cancer and normal tissues, RNA-seq data was used for 16/33 TCGA cancer types (*n* = 5507) containing gene expression data for corresponding normal tissue (*n* = 627; Table 1). For all analyzed cancer types, *BIRC5* levels were shown to be significantly higher in cancer tissue than corresponding normal tissues (Fig. 2A). These findings were confirmed using our previously published RNA microarray data for breast cancer, an Affymetrix HG-U133A RNA microarray dataset with 21 cancer types, and an Affymetrix HG-U133 Plus 2 RNA microarray dataset comprised of 25 cancer types (Fig. 2B-D). However, no statistically significant difference in expression patterns was found for heart and pancreas tissues in the Affymetrix HG-U133A dataset, as well as for adipose, endometrium, oral, and small intestinal tissues in the Affymetrix HG-U133 Plus 2 dataset. Moreover, normal samples derived from blood, prostate, testis, thyroid, and uterus also displayed significantly higher *BIRC5* expression patterns than their tumor counterparts.

### *BIRC5* is frequently co-expressed with genes involved in cell cycle and DNA replication

**Fig. 3:**
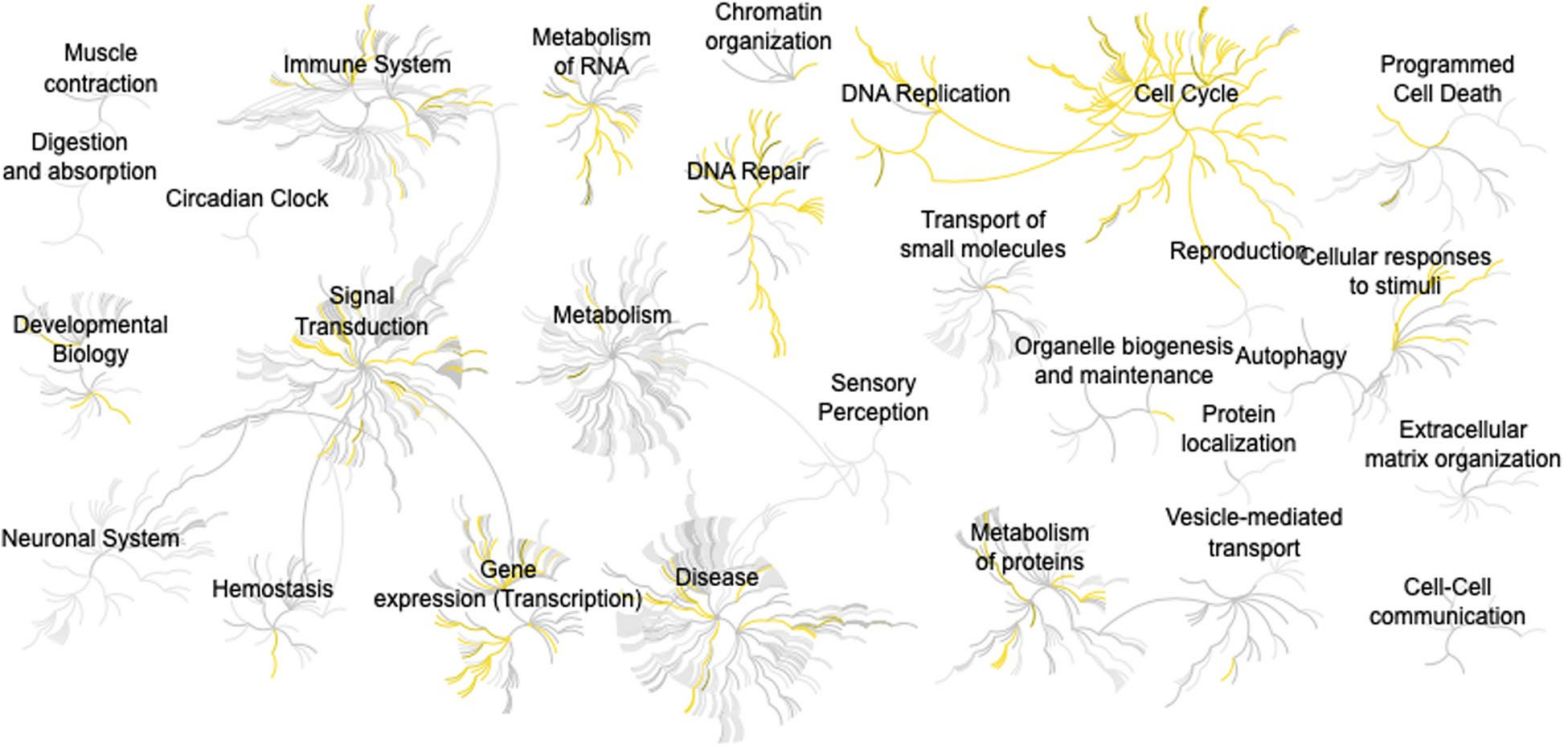
Pathway analysis for genes co-expressed with *BIRC5* using Reactome. Yellow depicts pathways containing genes co-expressed with *BIRC5*. Figure downloaded from www.reactome.org [20]

To identify genes recurrently co-expressed with *BIRC5* in cancer, the top 100 co-expressed genes in the 32 cancer types were extracted from the Spearman correlation analysis (*Q* < 0.05) in cBioPortal. When combining the top 100 co-expressed genes for each cancer type, some genes occurred more than once, e.g. *AURKB* (encoding for Aurora kinase B [47]) and *CDC20* (encoding for Cell division cycle 20 [48]) were the most frequent among the combined list of 3200 genes. In total, 117/3200 genes were negatively correlated with *BIRC5* and the remaining genes were positively correlated (Additional Table 3). When duplicates were removed and *BIRC5* was included in the list, 629 genes remained. The Reactome Pathway Database was then used to identify signaling pathways associated with *BIRC5* and the co-expressed genes. In total, 436/629 genes involving 1039 pathways were identified in Reactome, including pathways playing a pivotal role in cell cycle and DNA replication were found to be overrepresented (Fig. 3, Additional Table 3, Additional file 1).

### Survival analysis demonstrates the prognostic relevance of *BIRC5* expression in cancer

**Fig. 4:**
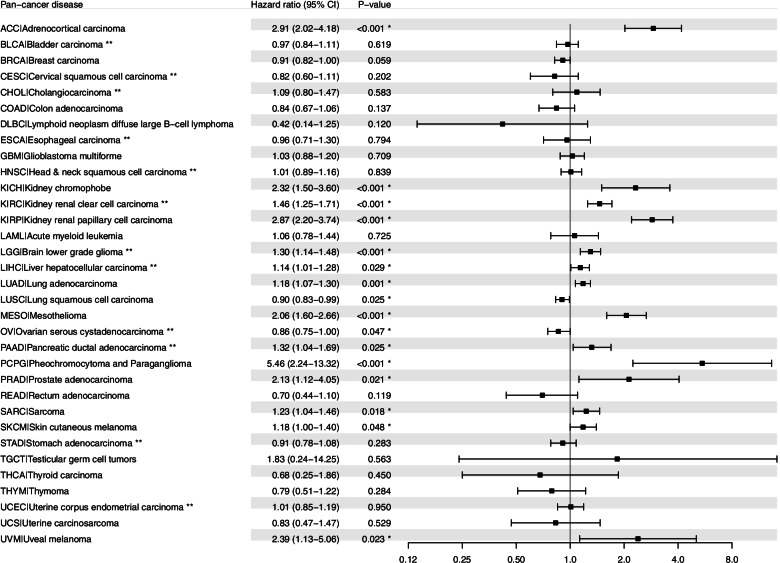
Forest plot of the association between overall survival (OS) and *BIRC5* expression. * denotes significant p-values. All cancer types are adjusted for age using multivariable Cox proportional hazards regression analysis; cancer types marked with ** are adjusted for both age and tumor grade

**Fig. 5:**
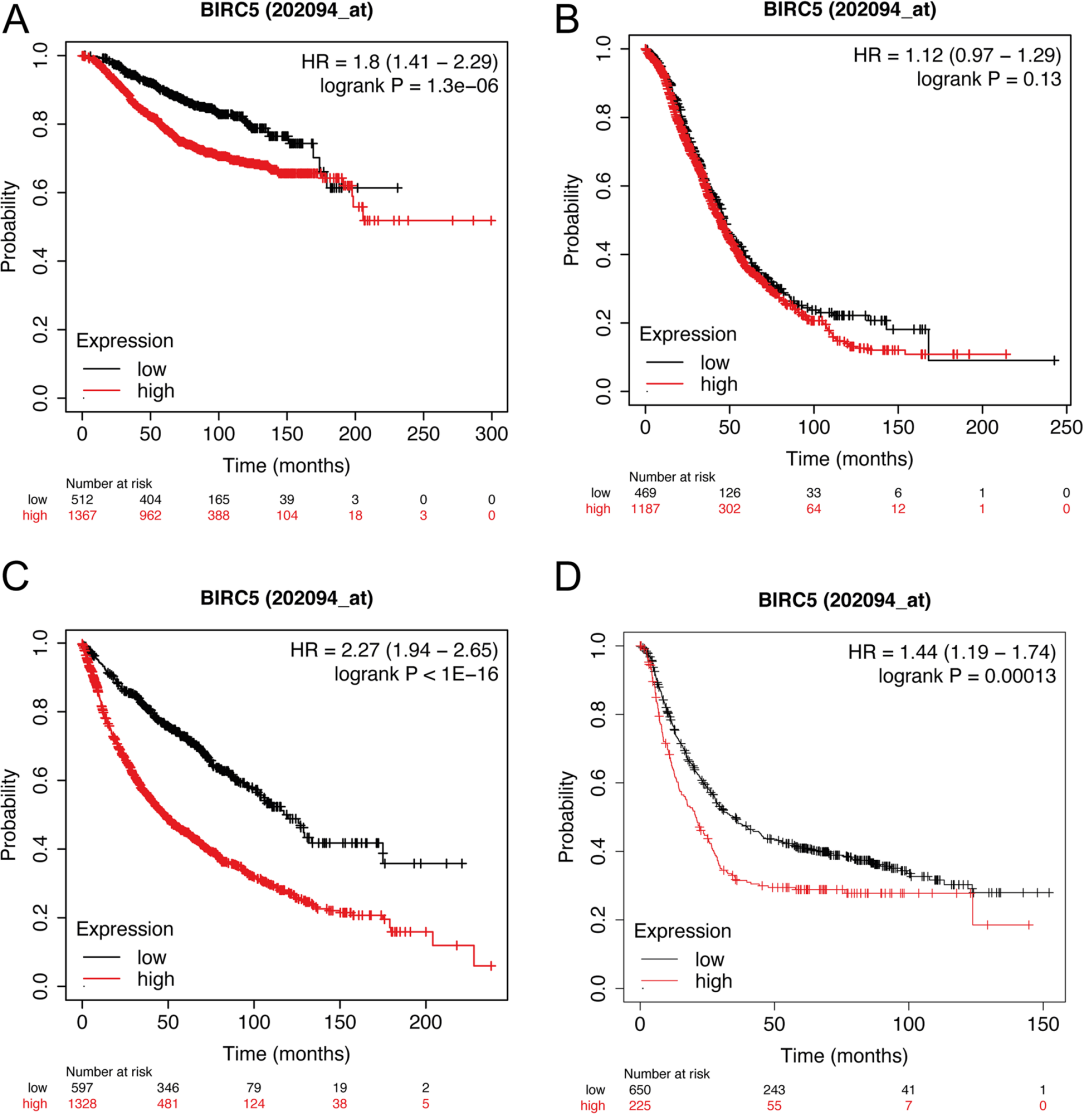
Kaplan-Meier curves from the KM plotter web-based tool showing the association between BIRC5 expression and overall survival (OS) for (**A**) breast cancer, (**B**) ovarian cancer, (**C**) lung cancer, and (**D**) gastric cancer. BIRC5 expression was associated with significantly more unfavorable OS for breast cancer, lung cancer, and gastric cancer

To assess the prognostic significance of *BIRC5* expression in the 33 cancer types, multivariable Cox regression analysis was performed for OS or PFI after adjusting for age and/or tumor grade. In total, 14/33 cancer types were found to be associated with more unfavorable OS in patients with tumor samples expressing *BIRC5*, whereas *BIRC5* expression was linked to a protective effect in 2/33 cancer types (LUSC and OV; Fig. 4). After adjusting for both age and tumor grade, *BIRC5* expression was found to have an adverse effect on OS in KIRC, LGG, LIHC, and PAAD (hazard ratio (HR) > 1, *P* < 0.05), whereas *BIRC5* expression was associated with a protective effect in OV (HR = 0.86; 95% confidence interval (95% CI): 0.75–1.00; *P* = 0.047). After adjusting for age alone, HR > 1 (*P* < 0.05) were found for ACC, KICH, KIRP, LUAD, MESO, PCPG, PRAD, SARC, SKCM and UVM, with the highest HR values found for PCPG (HR = 4.46; 95% CI: 2.24–13.32) and ACC (HR = 2.91; 95% CI: 2.02–4.18). Moreover, adjusting for age showed that *BIRC5* expression was associated with a protective effect in LUSC (HR = 0.90; 95% CI: 0.83–0.99). These data were validated using multivariable Cox regression analysis (adjusted for age and tumor grade) with RNA expression data from our previous work on breast cancer, as well as the web-based KM plotter tool for four different tumor types (breast-, ovarian-, lung-, and gastric cancer). The breast cancer dataset demonstrated that *BIRC5* expression was significantly associated with adverse OS rates (*BIRC5* probe ILMN_1803124: HR = 5.49, 95% CI: 2.32–12.97, *P* < 0.001; *BIRC5* probe ILMN_2349459: HR = 1.85, 95% CI: 1.27–2.6, *P* = 0.0014), while the Kaplan-Meier analysis of the four cancer types revealed that low *BIRC5* expression was significantly associated with better overall survival for breast-, lung and gastric cancer (Fig. 5).

**Fig. 6:**
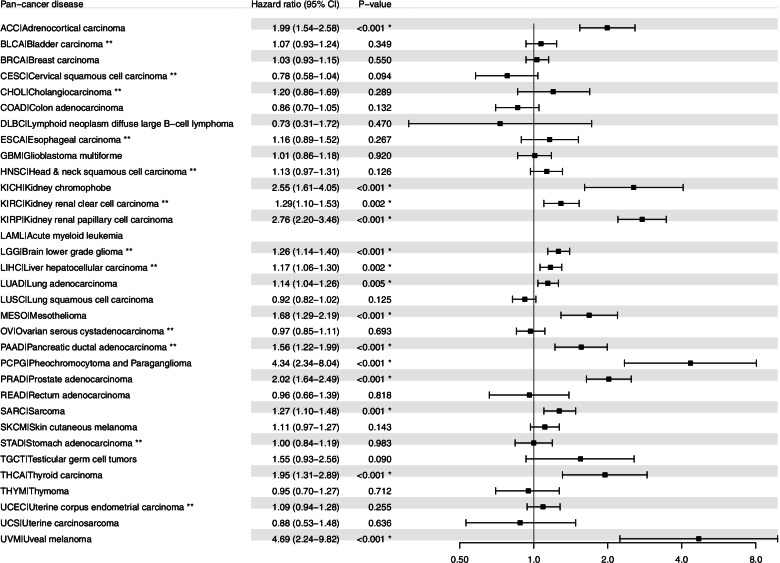
Forest plot of the association between progression-free interval (PFI) and *BIRC5* expression. * denotes significant p-values. All cancer types are adjusted for age using multivariable Cox proportional hazards regression analysis; cancer types marked with ** are adjusted for both age and tumor grade

Intriguingly, *BIRC5* expression was only associated with significantly more unfavorable PFI in 14/33 cancer types, with 4/14 cancer types (KIRC, LGG, LIHC, and PAAD) after adjusting for both age and tumor grade and 10/14 cancer types (ACC, KICH, KIRP, LUAD, MESO, PCPG, PRAD, SARC, THCA and UVM) after adjusting for age (Fig. 6). The highest HR was seen for UVM (HR = 4.69; 95% CI: 2.24–9.82) and PCPG (HR = 4.34; 95% CI: 2.34–8.04).

### Clinicopathological features and *BIRC5* expression

We then determined a relationship between clinicopathological features and BIRC5 expression stratified as high *BIRC5* (higher than median *BIRC5* expression) and low *BIRC5* (lower than median *BIRC5* expression) expression. Intriguingly, it was apparent that most cancer samples were classified as *BIRC5* high (Additional Table 4), with 9/33 tumor types (COAD, DLBC, GBM, LAML, OV, READ, SKCM, TGCT, UCS) only containing samples in the high *BIRC5* group. In most, but not all, tumor types, we observed a trend that patients in the high *BIRC5* group were generally younger at initial pathological diagnosis, e.g. ESCA (mean age 64.21 in high *BIRC5* group vs 78.50 in low *BIRC5* group; *P* = 0.001), KIRP (61.21 vs 64.48 years; *P* = 0.01), LGG (42.31 vs 47.83 years; P = 0.01), PCPG (44.15 vs 54.05 years; *P* < 0.001). Moreover, multiple cancer types were also found to be associated with *BIRC5* expression levels and T stage (BLCA, BRCA, KIRC, LUAD, PAAD, PRAD, STAD), N stage (BRCA, ESCA, PRAD, THCA), M stage (ESCA, KICH, KIRP, LUAD, LUSC), tumor stage (ESCA, KIRC, KIRP, LIHC, PAAD, UCEC), tumor grade (ESCA, KIRC, KIRP, LIHC, PAAD, UCEC), therapy success (BLCA, KIRP, LUAD, LUSC, PAAD, PRAD, STAD), and race/ethnicity (BRCA, KIRP, LGG, LIHC, PRAD, STAD).

## Discussion

Here, we applied a pan-cancer multiomics approach in 33 different cancer types to examine molecular mechanisms that can ultimately lead to the high *BIRC5* gene expression patterns observed in cancer. We show that, although genetic alterations are uncommon in the *BIRC5* gene, DNA amplification is associated with higher RNA levels of *BIRC5*. However, the clinical impact of genetic alterations such as DNA amplification in the *BIRC5* gene is still unclear. In agreement with previous studies [3], our results also show that *BIRC5* expression levels are higher in cancer tissue than normal tissue. In several different cancer types, we observe an association between higher *BIRC5* expression and unfavorable OS. Taken together, our findings demonstrate the prognostic relevance of *BIRC5* expression in a variety of cancer types from different organ systems.

The highest HR values for OS and *BIRC5* expression were found for adrenocortical carcinoma (ACC) and pheochromocytoma and paraganglioma (PCPG), both of which are hormone-producing tumors [49]. A previous study using immunohistochemistry to evaluate Survivin levels in ACC samples revealed overexpression of Survivin in carcinomas compared to adenomas or normal glands, with worse prognosis for patients with tumors expressing higher Survivin levels (not statistically significant). Knockdown of Survivin in an ACC cell line resulted in higher apoptotic rates [50]. Another study comparing Survivin expression in healthy adrenal medulla and pheochromocytoma/paraganglioma (malignant and benign) showed no significant difference between malignant or benign tumors. However, a more recent study showed an association between increased Survivin expression and worse prognosis in pheochromocytoma [51]. For uveal melanoma where we show an association between *BIRC5* expression and worse PFI, two previous studies showed conflicting results, one did not find any difference in immunohistochemical expression and tumor activity [52] and the other indicated the possible involvement of Survivin in Cisplatin-resistance using human uveal melanoma cell lines [53]. Our results show that high *BIRC5* expression is associated with worse prognosis in all three analyzed types of kidney cancer. This is in line with previous results from a meta-analysis on 10 studies containing 1063 renal cancer cases, which demonstrated that high Survivin expression is associated with TNM stage and Fuhrman grade [54]. Other studies have also found a connection with more aggressive renal tumors and high Survivin expression [55–57]. Our study proposes Survivin/*BIRC5* as a promising biomarker using RNA sequencing data. Survivin/*BIRC5* could be an addition to other biological detection indicators. A study investigating cervical cancer cell lines found that Survivin showed more intense fluorescence in cancer cells than in normal cervical cells. Although the authors found Survivin to have a clinical sensitivity of 72.5% and a specificity of 77%, the sensitivity increased to 98% when combining Survivin with HPV16E6 and 96.1% when only using HPV16E6 [58].

Unlike the tumor types discussed above, higher *BIRC5* expression seems to be beneficial for OS for patients with ovarian cancer. A previous study found an association between high Survivin and response to taxane-platinum treatment [10]. However, recently, two meta-analyses found that high Survivin expression in ovarian cancer is associated with poor prognosis and worse tumor stage [59, 60]. Further studies on *BIRC5* expression/Survivin protein levels are needed in order to determine its prognostic significance for ovarian cancer or its possible connection to chemotherapy response. Interestingly, it has been shown that wild type of the tumor suppressor gene p53 could subdue Survivin expression [61], suggesting that non-functional p53 in cancer could result in higher Survivin expression. Similar conclusions were also determined in a recent study showing elevated Survivin expression in mice with p53-mutated esophageal squamous cell carcinoma, which could play a role in aiding lung metastasis [62]. A clinical study examined the genome and transcriptome of 198 lung squamous cell carcinomas and found that *BIRC5* amplification was prevalent in tumors with p53 mutations [63].

There are several in vitro studies that show how *BIRC5* overexpression or silencing could affect cancer cell lines. For instance, *TP53* has been linked to *BIRC5* in both glioblastoma multiforme (GBM) cells and 5-fluorouracil resistant cholangiocarcinoma (CHOL) cell lines [64, 65]. In vitro studies have suggested that *BIRC5*/Survivin could be implicated in chemotherapy resistance of Irinotecan in colon adenocarcinoma (COAD), Oxaliplatin in esophageal squamous and esophageal adenocarcinoma (ESCA) and Cisplatin in hepatocellular carcinoma (LIHC) [66–68]. In breast cancer cell lines, Survivin, as well as *FOXM1* and *XIAP* have been shown to contribute to drug-resistance [69]. Silencing of Survivin in HeLa cells (cervical carcinoma cells) was shown to result in an increased sensitivity to radiation therapy [70]. Several studies on thyroid carcinoma (THYR) cell lines demonstrate the involvement of Survivin in inhibiting cell proliferation [71–73] and an in vivo study using human gastric adenocarcinoma cell lines in mice xenografts showed that inhibition of Survivin expression could promote cell death [74].

In conclusion, *BIRC5* is indeed overexpressed in most cancer types, which frequently correlates with patient clinical outcome. Although publicly available TCGA data are useful for explorative pan-cancer studies, these findings need to be examined further in specific tumor types at the protein level to assess the clinical utility of *BIRC5*/Survivin. A limitation of the current study was the lack of large datasets similar to the TCGA dataset that contained both gene expression and clinical data to validate the prognostic relevance of *BIRC5* expression in cancer. In future studies, it would also be interesting to evaluate the impact of *BIRC5* expression levels on chemotherapy efficacy. In oncology, there is a constant need for better predictive markers in order to choose the right course of treatment [75]. Some treatment regimens are not only associated with acute toxicity, but also long-lasting chronic complications [76, 77]. Our study suggests *BIRC5* as a promising prognostic biomarker for several cancer types, but these findings need to be investigated further.

## Supplementary Information

**Additional file 1.** Report Reactome 8th of October 2021.

**Additional file 2: Additional Table 1** *BIRC5* high impact mutations according to ICGC Data Portal. **Additional Table 2** *BIRC5* pathogenic mutations according to COSMIC. **Additional Table 3** Top 100 genes co-expressed with *BIRC5* for each cancer type. **Additional Table 4** *BIRC5* expression and clinicopathological features.

## Data Availability

The data used in this study have already been deposited in Gene Expression Omnibus (accession GSE97293), as stated in our previous publication [33]. The data bases referenced in the methods section of this article are all open access.
