# Supplementary material for: Pan-cancer analysis identifies BIRC5 as a prognostic biomarker
Source: BMC Cancer. 2022 Mar 25;22:322. doi: 10.1186/s12885-022-09371-0 (PMC8953143; doi:10.1186/s12885-022-09371-0)
Supplement: Supplementary file 1 — Additional file 1. Report Reactome 8th of October 2021. [file 12885_2022_9371_MOESM1_ESM.pdf]

reactome

Pathway Analysis Report

Correlated Genes

This report contains the pathway analysis results for the submitted sample 'Correlated Genes'. Analysis was performed against Reactome version 77 on 08/10/2021. The web link to these results is:

https://reactome.org/PathwayBrowser/#/ANALYSIS=MjAyMTEwMDgxMjU1MDhfNDI4OA%3D%3D

Please keep in mind that analysis results are temporarily stored on our server. The storage period depends on usage of the service but is at least 7 days. As a result, please note that this URL is only valid for a limited time period and it might have expired.

Table of Contents

1. Introduction
2. Properties
3. Genome-wide overview
4. Most significant pathways
5. Pathways details
6. Identifiers found
7. Identifiers not found

1. Introduction

Reactome is a curated database of pathways and reactions in human biology. Reactions can be considered as pathway 'steps'. Reactome defines a 'reaction' as any event in biology that changes the state of a biological molecule. Binding, activation, translocation, degradation and classical biochemical events involving a catalyst are all reactions. Information in the database is authored by expert biologists, entered and maintained by Reactome’s team of curators and editorial staff. Reactome content frequently cross-references other resources e.g. NCBI, Ensembl, UniProt, KEGG (Gene and Compound), ChEBI, PubMed and GO. Orthologous reactions inferred from annotation for Homo sapiens are available for 17 non-human species including mouse, rat, chicken, puffer fish, worm, fly, yeast, rice, and Arabidopsis. Pathways are represented by simple diagrams following an SBGN-like format.

Reactome's annotated data describe reactions possible if all annotated proteins and small molecules were present and active simultaneously in a cell. By overlaying an experimental dataset on these annotations, a user can perform a pathway over-representation analysis. By overlaying quantitative expression data or time series, a user can visualize the extent of change in affected pathways and its progression. A binomial test is used to calculate the probability shown for each result, and the p-values are corrected for the multiple testing (Benjamini–Hochberg procedure) that arises from evaluating the submitted list of identifiers against every pathway.

To learn more about our Pathway Analysis, please have a look at our relevant publications:

Fabregat A, Sidiropoulos K, Garapati P, Gillespie M, Hausmann K, Haw R, … D’Eustachio P (2016). The reactome pathway knowledgebase. Nucleic Acids Research, 44(D1), D481–D487. https://doi.org/10.1093/nar/gkv1351.

Fabregat A, Sidiropoulos K, Viteri G, Forner O, Marin-Garcia P, Arnau V, … Hermjakob H (2017). Reactome pathway analysis: a high-performance in-memory approach. BMC Bioinformatics, 18.

2. Properties

- This is an **overrepresentation** analysis: A statistical (hypergeometric distribution) test that determines whether certain Reactome pathways are over-represented (enriched) in the submitted data. It answers the question 'Does my list contain more proteins for pathway X than would be expected by chance?' This test produces a probability score, which is corrected for false discovery rate using the Benjamani-Hochberg method.
- 436 out of 629 identifiers in the sample were found in Reactome, where 1039 pathways were hit by at least one of them.
- All non-human identifiers have been converted to their human equivalent.
- This report is filtered to show only results for species 'Homo sapiens' and resource 'all resources'.
- The unique ID for this analysis (token) is MjAyMTEwMDgxMjU1MDhfNDI4OA%3D%3D. This ID is valid for at least 7 days in Reactome's server. Use it to access Reactome services with your data.

3. Genome-wide overview

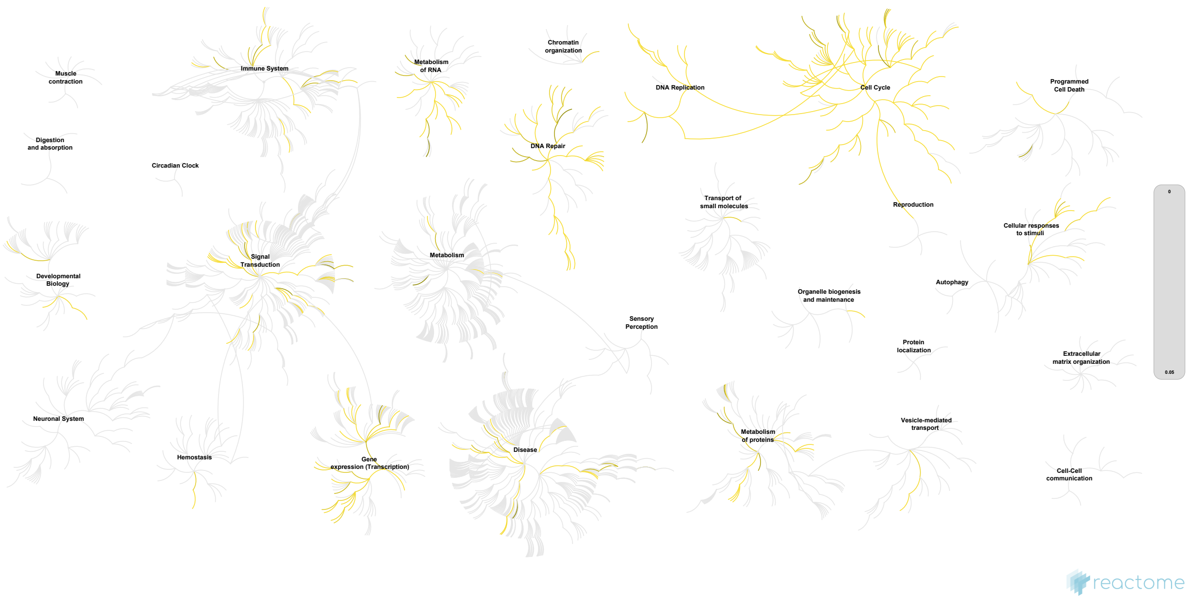

This figure shows a genome-wide overview of the results of your pathway analysis. Reactome pathways are arranged in a hierarchy. The center of each of the circular "bursts" is the root of one top-level pathway, for example "DNA Repair". Each step away from the center represents the next level lower in the pathway hierarchy. The color code denotes over-representation of that pathway in your input dataset. Light grey signifies pathways which are not significantly over-represented.

4. Most significant pathways

The following table shows the 25 most relevant pathways sorted by p-value.

Pathway name	Entities				Reactions	
	found	ratio	p-value	FDR*	found	ratio
G1/S-Specific Transcription	25 / 43	0.003	1.11e-16	4.88e-15	28 / 28	0.002
Amplification of signal from unattached kinetochores via a MAD2 inhibitory signal	37 / 94	0.006	1.11e-16	4.88e-15	4 / 4	2.96e-04
Amplification of signal from the kinetochores	37 / 94	0.006	1.11e-16	4.88e-15	4 / 4	2.96e-04
Mitotic Spindle Checkpoint	41 / 111	0.008	1.11e-16	4.88e-15	7 / 7	5.18e-04
G2/M Checkpoints	45 / 154	0.011	1.11e-16	4.88e-15	24 / 24	0.002
G1/S Transition	56 / 150	0.01	1.11e-16	4.88e-15	57 / 61	0.005
Mitotic G1 phase and G1/S transition	65 / 173	0.012	1.11e-16	4.88e-15	92 / 99	0.007
Synthesis of DNA	44 / 133	0.009	1.11e-16	4.88e-15	24 / 26	0.002
Chromosome Maintenance	41 / 138	0.009	1.11e-16	4.88e-15	35 / 38	0.003
DNA Replication	50 / 142	0.01	1.11e-16	4.88e-15	42 / 47	0.003
Resolution of Sister Chromatid Cohesion	43 / 134	0.009	1.11e-16	4.88e-15	7 / 8	5.92e-04
Separation of Sister Chromatids	59 / 195	0.013	1.11e-16	4.88e-15	7 / 8	5.92e-04
S Phase	54 / 180	0.012	1.11e-16	4.88e-15	47 / 54	0.004
Cell Cycle, Mitotic	162 / 596	0.041	1.11e-16	4.88e-15	303 / 350	0.026
DNA Replication Pre-Initiation	32 / 88	0.006	1.11e-16	4.88e-15	18 / 21	0.002
Mitotic Prometaphase	54 / 211	0.015	1.11e-16	4.88e-15	17 / 20	0.001
Cell Cycle	187 / 734	0.05	1.11e-16	4.88e-15	377 / 449	0.033
M Phase	90 / 416	0.029	1.11e-16	4.88e-15	64 / 91	0.007
Cell Cycle Checkpoints	89 / 280	0.019	1.11e-16	4.88e-15	39 / 56	0.004
Mitotic Metaphase and Anaphase	69 / 250	0.017	1.11e-16	4.88e-15	22 / 33	0.002
Mitotic Anaphase	68 / 249	0.017	1.11e-16	4.88e-15	21 / 32	0.002
EML4 and NUDC in mitotic spindle formation	39 / 121	0.008	1.11e-16	4.88e-15	3 / 5	3.70e-04
RHO GTPases Activate Formins	40 / 149	0.01	1.11e-16	4.88e-15	4 / 27	0.002
Mitotic G2-G2/M phases	46 / 214	0.015	3.33e-16	1.43e-14	72 / 80	0.006
RHO GTPase Effectors	57 / 326	0.022	5.55e-16	2.28e-14	35 / 113	0.008

* False Discovery Rate

5. Pathways details

For every pathway of the most significant pathways, we present its diagram, as well as a short summary, its bibliography and the list of inputs found in it.

1. G1/S-Specific Transcription (R-HSA-69205)

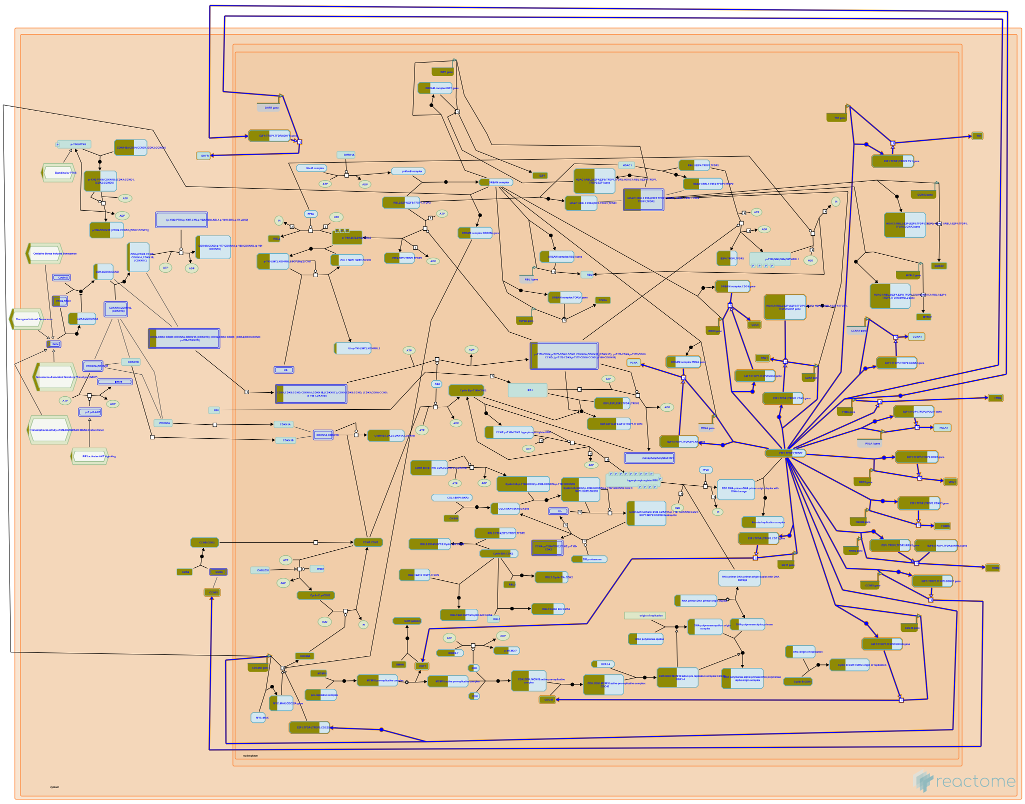

Cellular compartments: nucleoplasm.

The E2F family of transcription factors regulate the transition from the G1 to the S phase in the cell cycle. E2F activity is regulated by members of the retinoblastoma protein (pRb) family, resulting in the tight control of the expression of E2F-responsive genes. Phosphorylation of pRb by cyclin D:CDK complexes releases pRb from E2F, inducing E2F-targeted genes such as cyclin E.

E2F1 binds to E2F binding sites on the genome activating the synthesis of the target proteins. For annotation purposes, the reactions regulated by E2F1 are grouped under this pathway and information about the target genes alone are displayed for annotation purposes.

Cellular targets for activation by E2F1 include thymidylate synthase (TYMS) (DeGregori et al. 1995), Rir2 (RRM2) (DeGregori et al. 1995, Giangrande et al. 2004), Dihydrofolate reductase (DHFR) (DeGregori et al. 1995, Wells et al. 1997, Darbinian et al. 1999), Cdc2 (CDK1) (Furukawa et al. 1994, DeGregori et al. 1995, Zhu et al. 2004), Cyclin A1 (CCNA1) (DeGregori et al. 1995, Liu et al. 1998), CDC6 (DeGregori et al. 1995, Yan et al. 1998; Ohtani et al. 1998), CDT1 (Yoshida and Inoue 2004), CDC45 (Arata et al. 2000), Cyclin E (CCNE1) (Ohtani et al. 1995), Emi1 (FBXO5) (Hsu et al. 2002), and ORC1 (Ohtani et al. 1996, Ohtani et al. 1998). The activation of TK1 (Dnk1) (Dou et al. 1994, DeGregori et al. 1995, Giangrande et al. 2004) and CDC25A (DeGregori et al. 1995, Vigo et al. 1999) by E2F1 is conserved in Drosophila (Duronio and O'Farrell 1994, Reis and Edgar 2004).

RRM2 protein is involved in dNTP level regulation and activation of this enzyme results in higher levels of dNTPs in anticipation of S phase. E2F activation of RRM2 has been shown also in Drosophila by Duronio and O'Farrell (1994). E2F1 activation of CDC45 is shown in mouse cells by using human E2F1 construct (Arata et al. 2000). Cyclin E is also transcriptionally regulated by E2F1. Cyclin E protein plays important role in the transition of G1 in S phase by associating with CDK2 (Ohtani et al. 1996). E2F1-mediated activation of PCNA has been demonstrated in Drosophila (Duronio and O'Farrell 1994) and in some human cells by using recombinant adenovirus constructs (DeGregori et al. 1995). E2F1-mediated activation of the DNA polymerase alpha subunit p180 (POLA1) has been demonstrated in some human cells. It has also been demonstrated in Drosophila by Ohtani and Nevins (1994). It has been observed in Drosophila that E2F1 induced expression of Orc1 stimulates ORC1 6 complex formation and binding to the origin of replication (Asano and Wharton 1999). ORC1 6 recruit CDC6 and CDT1 that are required to recruit the MCM2 7 replication helicases. E2F1 regulation incorporates a feedback mechanism wherein Geminin (GMNN) can inhibit MCM2 7 recruitment of ORC1 6 complex by interacting with CDC6/CDT1. The activation of CDC25A and TK1 (Dnk1) by E2F1 has been inferred from similar events in Drosophila (Duronio RJ and O'Farrell 1994; Reis and Edgar 2004). E2F1 activates string (CDC25) that in turn activates the complex of Cyclin B and CDK1. A similar phenomenon has been observed in mouse NIH 3T3 cells and in Rat1 cells.

References

DeGregori J, Kowalik T & Nevins JR (1995). Cellular targets for activation by the E2F1 transcription factor include DNA synthesis- and G1/S-regulatory genes. Mol Cell Biol, 15, 4215-24.

Yoshida K & Inoue I (2004). Regulation of Geminin and Cdt1 expression by E2F transcription factors . Oncogene, 23, 3802-12.

Arata Y, Fujita M, Ohtani K, Kijima S & Kato JY (2000). Cdk2-dependent and -independent pathways in E2F-mediated S phase induction. J Biol Chem, 275, 6337-45.

Yan Z, DeGregori J, Shohet R, Leone G, Stillman B, Nevins JR & Williams RS (1998). Cdc6 is regulated by E2F and is essential for DNA replication in mammalian cells. Proc Natl Acad Sci U S A, 95, 3603-8.

Ohtani K, Tsujimoto A, Ikeda M & Nakamura M (1998). Regulation of cell growth-dependent expression of mammalian CDC6 gene by the cell cycle transcription factor E2F. Oncogene, 17, 1777-85.

Edit history

Date	Action	Author
2003-06-05	Created	Walworth N, O'Donnell M
2018-12-21	Modified	D'Eustachio P

Entities found in this pathway (15)

Input	UniProt Id	Input	UniProt Id	Input	UniProt Id
CCNE1	P24864	CDC45	O75419	CDC6	Q99741
CDK1	P06493	CDT1	Q9H211	E2F1	Q01094
E2F2	Q16254	FBXO5	Q9UKT4	ORC1	Q13415
RBL2	Q08999	RRM2	P31350	TFDP1	Q14186
TK1	P04183	TYMS	P04818		

Input	Ensembl Id	Input	Ensembl Id	Input	Ensembl Id
CCNE1	ENSG00000105173	CDC25A	ENSG00000164045	CDC45	ENSG00000093009
CDC6	ENSG00000094804	CDK1	ENSG00000170312	CDT1	ENSG00000167513
FBXO5	ENSG00000112029	ORC1	ENSG00000085840	RRM2	ENSG00000171848
TK1	ENSG00000167900	TYMS	ENSG00000176890		

2. Amplification of signal from unattached kinetochores via a MAD2 inhibitory signal (R-HSA-141444)

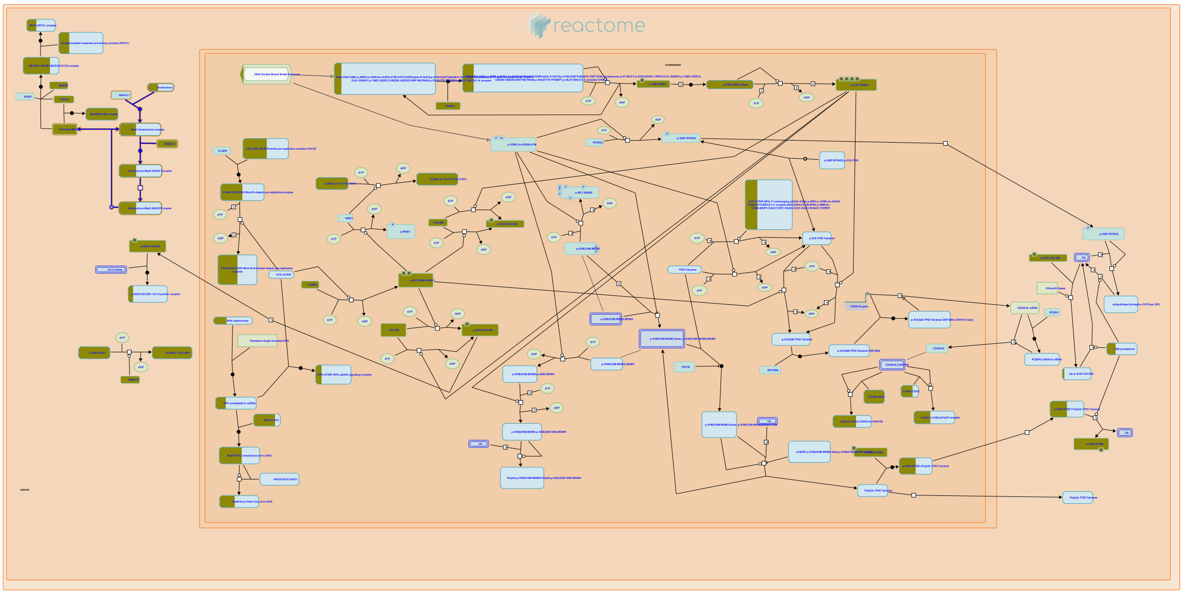

Cellular compartments: cytosol.

The signal from unattached kinetochores is amplified through a Mad2 inhibitory signal that is propagated by the binding of Mad1 to the kinetochore, the association of Mad2 with Mad1, the conversion of Mad2 conformation to an inhibitory form through its association with Mad1 and finally the release of the inhibitory form of Mad2 from the kinetochore.

References

Chan GK & Yen TJ (2003). The mitotic checkpoint: a signaling pathway that allows a single unattached kinetochore to inhibit mitotic exit. Prog Cell Cycle Res, 5, 431-9.

Edit history

Date	Action	Author
2004-05-05	Authored	Yen TJ
2004-05-05	Created	Yen TJ
2021-05-31	Modified	Shorser S

Entities found in this pathway (38)

Input	UniProt Id	Input	UniProt Id	Input	UniProt Id
AURKA	Q96GD4	AURKB	Q96GD4	BIRC5	O15392
BUB1	O43683, O60566	BUB1B	O60566	CDC20	Q12834
CDCA8	Q53HL2	CENPA	P49450	CENPE	Q02224
CENPF	P49454	CENPH	Q9H3R5	CENPK	Q9BS16
CENPL	Q8N0S6	CENPM	Q9NSP4	CENPN	Q96H22
CENPO	Q9BU64	CENPU	Q71F23	DSN1	Q9H410
ERCC6L	Q2NKX8	INCENP	Q9NQS7	KIF18A	Q8NI77
KIF2C	Q99661	MAD2L1	Q13257	NDC80	O14777
NUDC	Q9Y266	NUF2	Q9BZD4	NUP37	Q8NFH4

Input	UniProt Id	Input	UniProt Id	Input	UniProt Id
NUP85	Q9BW27	PLK1	P53350	RANBP2	P49792
RCC2	Q9P258	SGO1	Q5FBB7	SGO2	Q562F6
SKA1	Q96BD8	SKA2	Q8WVK7	SPC24	Q8NBT2
SPC25	Q9HBM1	ZWINT	O95229		

3. Amplification of signal from the kinetochores (R-HSA-141424)

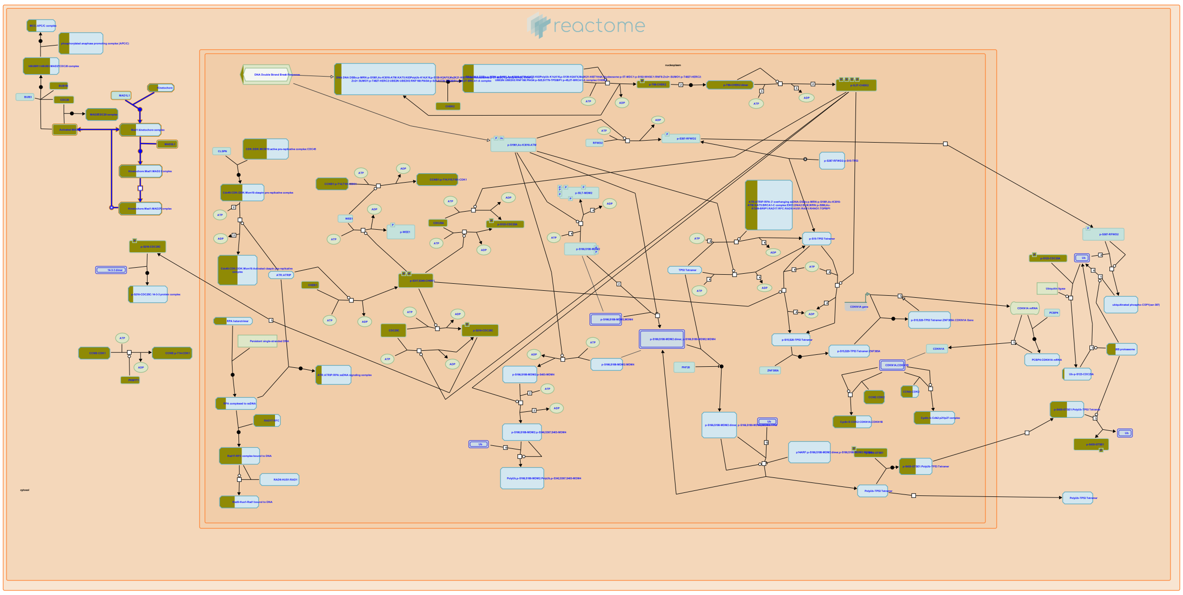

Cellular compartments: cytosol.

A single unattached kinetochore is capable of preventing cells from exiting mitosis. The mitotic checkpoint provides a way for a localized defect to affect the global biochemical status of the cell. In principle, the signal that is generated at an unattached kinetochore diffuses throughout the cell to affect its target. There are currently two models for how this is achieved. One model is based on the observation that the Mad2 checkpoint protein binds and is rapidly released from unattached kinetochores. The kinetochore is believed to act as a catalyst that converts Mad2 into an inhibitory state that diffuses throughout the cell upon its release from the kinetochore. A second model proposes that the signal is amplified by a kinase cascade much like a conventional signal transduction pathway. This kinase cascade is believed to be comprised of the checkpoint kinases, hBUBR1, hBUB1, hMPS1.

References

Chan GK & Yen TJ (2003). The mitotic checkpoint: a signaling pathway that allows a single unattached kinetochore to inhibit mitotic exit. Prog Cell Cycle Res, 5, 431-9.

Edit history

Date	Action	Author
2004-05-05	Authored	Yen TJ
2004-05-05	Created	Yen TJ
2021-05-21	Modified	Shorser S

Entities found in this pathway (38)

Input	UniProt Id	Input	UniProt Id	Input	UniProt Id
AURKA	Q96GD4	AURKB	Q96GD4	BIRC5	O15392
BUB1	O43683, O60566	BUB1B	O60566	CDC20	Q12834
CDCA8	Q53HL2	CENPA	P49450	CENPE	Q02224
CENPF	P49454	CENPH	Q9H3R5	CENPK	Q9BS16

Input	UniProt Id	Input	UniProt Id	Input	UniProt Id
CENPL	Q8N0S6	CENPM	Q9NSP4	CENPN	Q96H22
CENPO	Q9BU64	CENPU	Q71F23	DSN1	Q9H410
ERCC6L	Q2NKX8	INCENP	Q9NQS7	KIF18A	Q8NI77
KIF2C	Q99661	MAD2L1	Q13257	NDC80	O14777
NUDC	Q9Y266	NUF2	Q9BZD4	NUP37	Q8NFH4
NUP85	Q9BW27	PLK1	P53350	RANBP2	P49792
RCC2	Q9P258	SGO1	Q5FBB7	SGO2	Q562F6
SKA1	Q96BD8	SKA2	Q8WVK7	SPC24	Q8NBT2
SPC25	Q9HBM1	ZWINT	O95229		

4. Mitotic Spindle Checkpoint (R-HSA-69618)

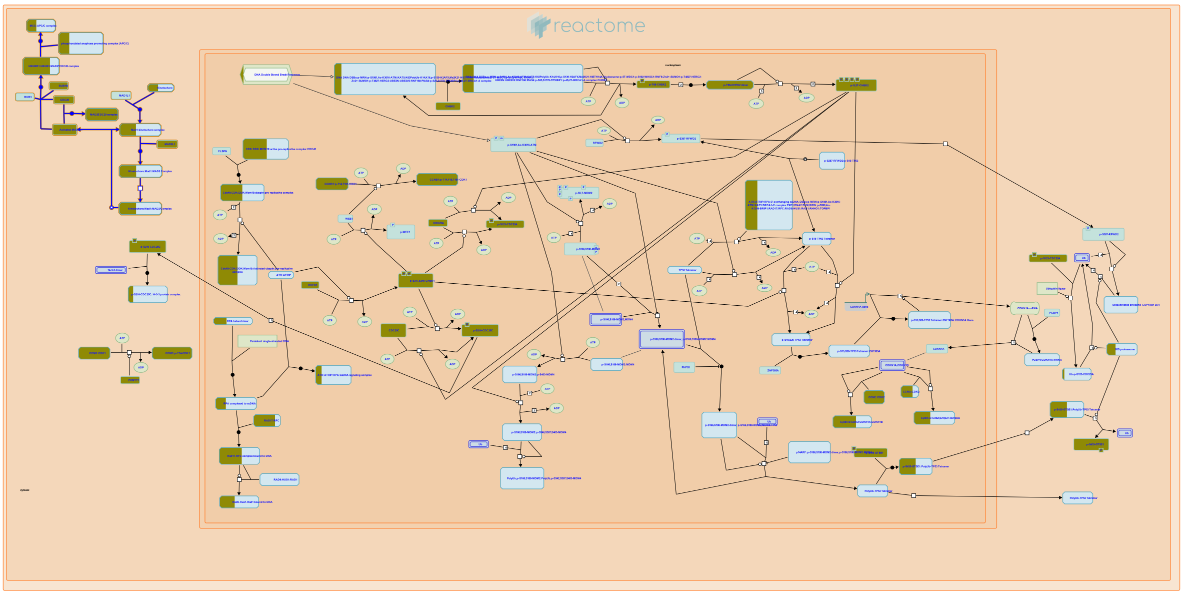

Cellular compartments: cytosol.

The mitotic checkpoint or spindle assembly checkpoint is an evolutionarily conserved mechanism that ensures that cells with misaligned chromosomes do not exit mitosis and divide to form aneuploid cells. As chromosome attachment to the spindle microtubules is a stochastic process, not all chromosomes achieve alignment at the spindle equator at the same time. It is therefore essential that even a single unaligned chromosome can prevent the onset of anaphase. The ability of the checkpoint to monitor the status of chromosome alignment is achieved by assigning checkpoint proteins to the kinetochore, a macromolecular complex that resides at centromeres of chromosomes that establishes connections with spindle microtubules.

The checkpoint proteins monitor, in an unknown way, the mechanical activities between kinetochore-associated proteins and microtubules. Defects in mechanical activities at kinetochores activate the resident checkpoint proteins to initiate a signal that is amplified throughout the cell that ultimately prevents the activation of the proteolytic process that is required for sister chromatid separation and the onset of anaphase.
Kinetochores of unaligned chromosomes differ from those of aligned chromosomes in two ways. Kinetochores of aligned chromosomes are saturated with between 20 to 30 microtubules. In addition, poleward directed forces exerted at each sister kinetochore generates tension between them. Unaligned kinetochores on the other hand, are not saturated with microtubules and are not under tension. The mitotic checkpoint detects the presence of unattached kinetochores rather than monitoring for the presence of attached kinetochores. Consequently, unattached kinetochores emit an inhibitory signal that inhibits the biochemical events that are required to initiate the onset of anaphase. The mechanism by which this inhibitory signal is generated at unattached kinetochores has not precisely been determined but the signal is generated as a result of the lack of microtubule occupancy and kinetochore tension.
A single unattached kinetochore is capable of preventing cells from exiting mitosis. The mitotic checkpoint provides a way for a localized defect to affect the global biochemical status of the cell. In principle, the signal that is generated at an unattached kinetochore diffuses throughout the cell to affect its target. There are currently two models for how this is achieved. One model is based on the observation that the Mad2 checkpoint protein binds and is rapidly released from unattached kinetochores. The kinetochore is believed to act as a catalyst that converts Mad2 into an inhibitory state that diffuses throughout the cell upon its release from the kinetochore. A second model proposes that the signal is amplified by a kinase cascade much like a conventional signal transduction pathway. This kinase cascade is believed to be comprised of the checkpoint kinases, hBUBR1, hBUB1, hMPS1.

References

Chan GK & Yen TJ (2003). The mitotic checkpoint: a signaling pathway that allows a single unattached kinetochore to inhibit mitotic exit. Prog Cell Cycle Res, 5, 431-9.

Musacchio A & Hardwick KG (2002). The spindle checkpoint: structural insights into dynamic signalling. Nat Rev Mol Cell Biol, 3, 731-41.

Edit history

Date	Action	Author
2004-05-05	Authored	Yen TJ
2004-05-05	Created	Yen TJ
2021-05-22	Modified	Shorser S

Entities found in this pathway (42)

Input	UniProt Id	Input	UniProt Id	Input	UniProt Id
ANAPC11	Q9NYG5	ANAPC15	P60006	AURKA	Q96GD4
AURKB	Q96GD4	BIRC5	O15392	BUB1	O43683, O60566
BUB1B	O60566	CDC20	Q12834	CDCA8	Q53HL2
CENPA	P49450	CENPE	Q02224	CENPF	P49454
CENPH	Q9H3R5	CENPK	Q9BS16	CENPL	Q8N0S6

Input	UniProt Id	Input	UniProt Id	Input	UniProt Id
CENPM	Q9NSP4	CENPN	Q96H22	CENPO	Q9BU64
CENPU	Q71F23	DSN1	Q9H410	ERCC6L	Q2NKX8
INCENP	Q9NQS7	KIF18A	Q8NI77	KIF2C	Q99661
MAD2L1	Q13257	NDC80	O14777	NUDC	Q9Y266
NUF2	Q9BZD4	NUP37	Q8NFH4	NUP85	Q9BW27
PLK1	P53350	RANBP2	P49792	RCC2	Q9P258
SGO1	Q5FBB7	SGO2	Q562F6	SKA1	Q96BD8
SKA2	Q8WVK7	SPC24	Q8NBT2	SPC25	Q9HBM1
UBE2C	O00762	UBE2S	Q16763	ZWINT	O95229

5. G2/M Checkpoints (R-HSA-69481)

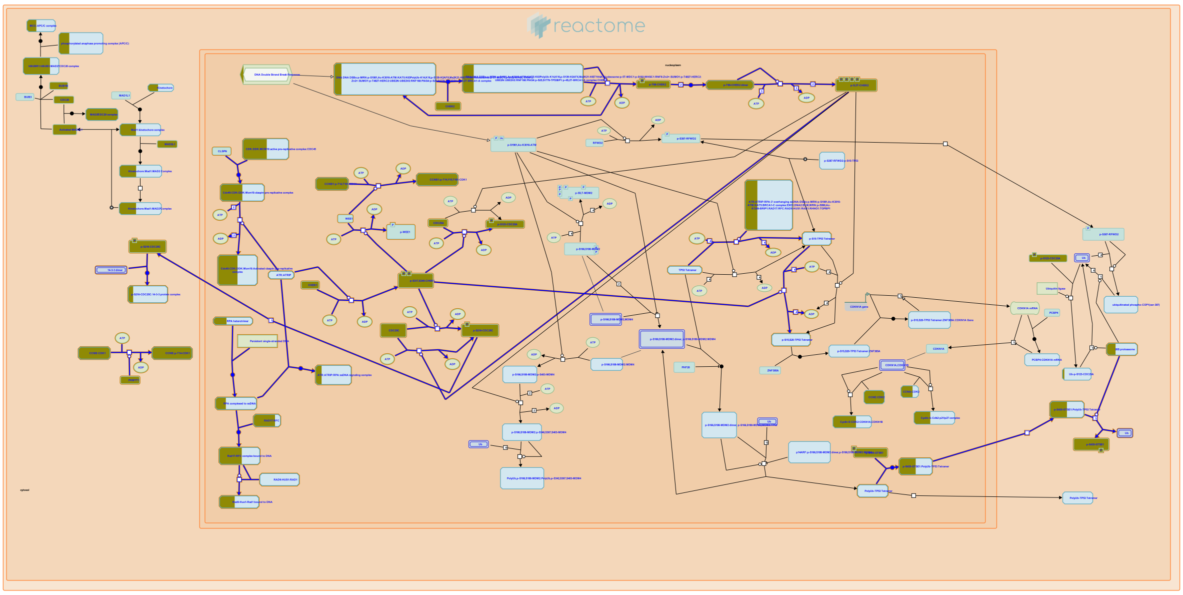

G2/M checkpoints include the checks for damaged DNA, unreplicated DNA, and checks that ensure that the genome is replicated once and only once per cell cycle. If cells pass these checkpoints, they follow normal transition to the M phase. However, if any of these checkpoints fail, mitotic entry is prevented by specific G2/M checkpoint events.

The G2/M checkpoints can fail due to the presence of unreplicated DNA or damaged DNA. In such instances, the cyclin-dependent kinase, Cdc2(Cdk1), is maintained in its inactive, phosphorylated state, and mitotic entry is prevented. Events that ensure that origins of DNA replication fire once and only once per cell cycle are also an example of a G2/M checkpoint.

In the event of high levels of DNA damage, the cells may also be directed to undergo apoptosis (not covered).

References

Edit history

Date	Action	Author
2003-06-05	Created	Walworth N, O'Donnell M
2021-05-22	Modified	Shorser S

Entities found in this pathway (42)

Input	UniProt Id	Input	UniProt Id	Input	UniProt Id
BLM	P54132	BRIP1	Q9BX63	CCNB1	P14635
CCNB2	O95067	CDC25A	P30304	CDC25C	P30307
CDC45	O75419	CDC6	Q99741	CDK1	P06493
CDK2	P24941	CHEK1	O14757	CHEK2	O96017
DBF4	Q9UBU7	EXO1	Q9UQ84	GTSE1	Q9NYZ3
H2AFX	P16104	HIST1H2BH	P62807, Q93079	MCM10	Q7L590
MCM2	P33993, P49736	MCM5	P33992	MCM6	Q14566
MCM7	P33993	ORC1	Q13415	ORC6	Q9Y5N6
PKMYT1	Q99640	PSMA2	P25787	PSMA4	P25789

Input	UniProt Id	Input	UniProt Id	Input	UniProt Id
PSMA6	P60900	PSMB3	P49720	PSMB4	P28070
PSMB7	P40306, Q99436	PSMC1	P62191	PSMC3	P17980
PSMC4	P43686	PSMC5	P62195	PSMD12	O00232
PSMD3	O43242	RFC2	P35249, P35250	RFC4	P35249, P35250
RFC5	P40937, P40938	RMI2	Q96E14	RPA3	P35244

6. G1/S Transition (R-HSA-69206)

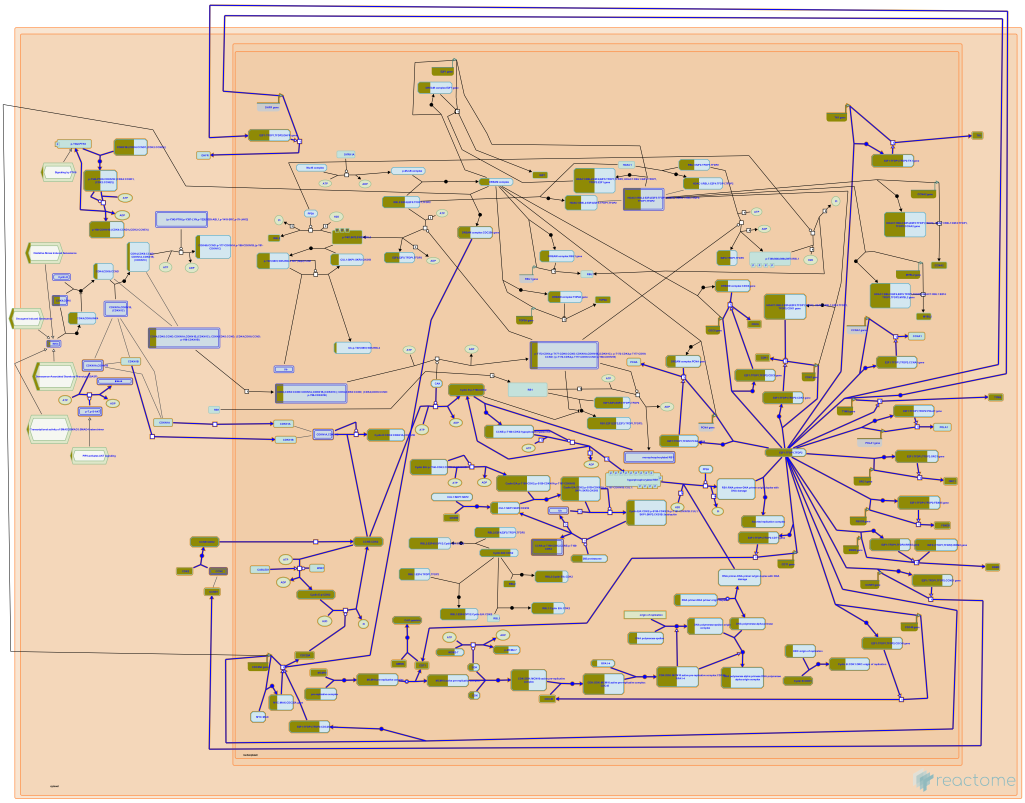

Cyclin E - Cdk2 complexes control the transition from G1 into S-phase. In this case, the binding of p21Cip1/Waf1 or p27kip1 is inhibitory. Important substrates for Cyclin E - Cdk2 complexes include proteins involved in the initiation of DNA replication. The two Cyclin E proteins are subjected to ubiquitin-dependent proteolysis, under the control of an E3 ubiquitin ligase known as the SCF. Cyclin A - Cdk2 complexes, which are also regulated by p21Cip1/Waf1 and p27kip1, are likely to be important for continued DNA synthesis, and progression into G2. An additional level of control of Cdk2 is reversible phosphorylation of Threonine-14 (T14) and Tyrosine-15 (Y15), catalyzed by the Wee1 and Myt1 kinases, and dephosphorylation by the three Cdc25 phosphatases, Cdc25A, B and C.

References

Edit history

Date	Action	Author
2003-06-05	Created	Walworth N, O'Donnell M
2021-05-22	Modified	Shorser S

Entities found in this pathway (44)

Input	UniProt Id	Input	UniProt Id	Input	UniProt Id
CCNA2	P20248	CCNB1	P14635	CCNE1	P24864
CCNE2	O96020	CDC25A	P30304	CDC45	O75419
CDC6	Q99741	CDK1	P06493, P24941	CDK2	P24941
CDK4	P11802	CDT1	Q9H211	CKS1B	P61024
DBF4	Q9UBU7	E2F1	Q01094	E2F2	Q16254
FBXO5	Q9UKT4	GMNN	O75496	MCM10	Q7L590
MCM2	P33993, P49736	MCM5	P33992	MCM6	Q14566

Input	UniProt Id	Input	UniProt Id	Input	UniProt Id
MCM7	P33993	ORC1	Q13415	ORC6	Q9Y5N6
POLA2	Q14181	POLE2	P56282	PSMA2	P25787
PSMA4	P25789	PSMA6	P60900	PSMB3	P49720
PSMB4	P28070	PSMB7	P40306, Q99436	PSMC1	P62191
PSMC3	P17980	PSMC4	P43686	PSMC5	P62195
PSMD12	O00232	PSMD3	O43242	RBL2	Q08999
RPA3	P35244	RRM2	P31350	TFDP1	Q14186
TK1	P04183	TYMS	P04818		

Input	Ensembl Id	Input	Ensembl Id	Input	Ensembl Id
CCNE1	ENSG00000105173	CDC25A	ENSG00000164045	CDC45	ENSG00000093009
CDC6	ENSG00000094804	CDK1	ENSG00000170312	CDT1	ENSG00000167513
FBXO5	ENSG00000112029	ORC1	ENSG00000085840	RRM2	ENSG00000171848
TK1	ENSG00000167900	TYMS	ENSG00000176890		

7. Mitotic G1 phase and G1/S transition (R-HSA-453279)

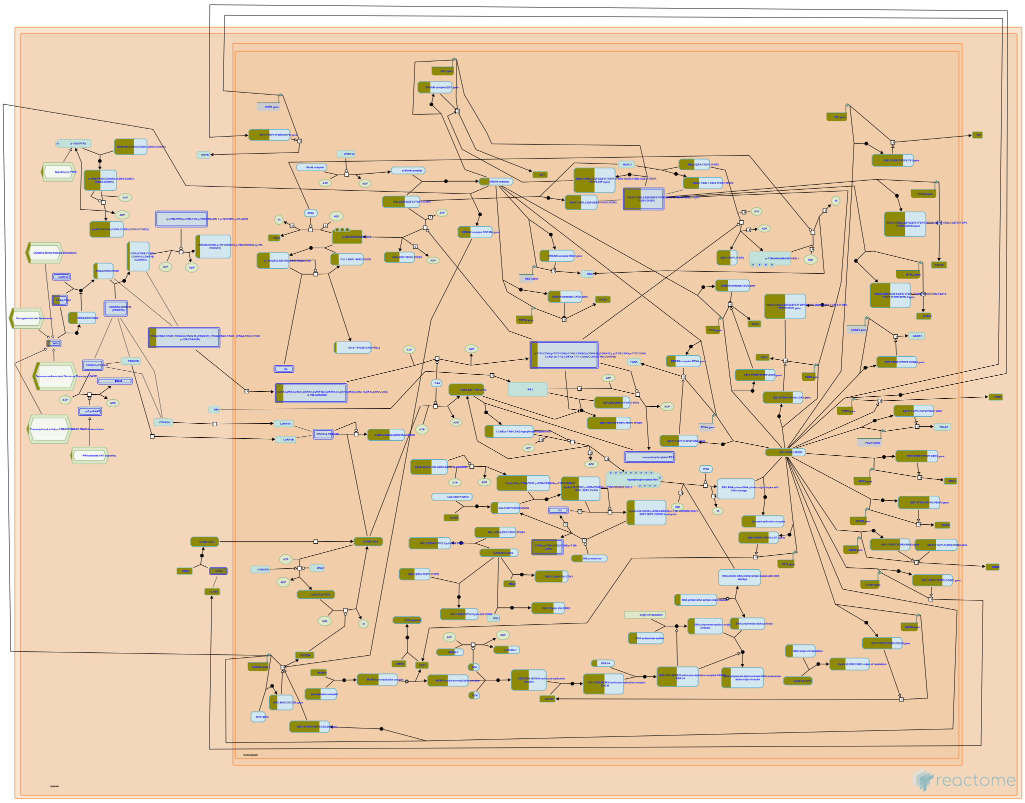

Mitotic G1-G1/S phase involves G1 phase of the mitotic interphase and G1/S transition, when a cell commits to DNA replication and divison genetic and cellular material to two daughter cells.

During early G1, cells can enter a quiescent G0 state. In quiescent cells, the evolutionarily conserved DREAM complex, consisting of the pocket protein family member p130 (RBL2), bound to E2F4 or E2F5, and the MuvB complex, represses transcription of cell cycle genes (reviewed by Sadasivam and DeCaprio 2013).

During early G1 phase in actively cycling cells, transcription of cell cycle genes is repressed by another pocket protein family member, p107 (RBL1), which forms a complex with E2F4 (Ferreira et al. 1998, Cobrinik 2005). RB1 tumor suppressor, the product of the retinoblastoma susceptibility gene, is the third member of the pocket protein family. RB1 binds to E2F transcription factors E2F1, E2F2 and E2F3 and inhibits their transcriptional activity, resulting in prevention of G1/S transition (Chellappan et al. 1991, Bagchi et al. 1991, Chittenden et al. 1991, Lees et al. 1993, Hiebert 1993, Wu et al. 2001). Once RB1 is phosphorylated on serine residue S795 by Cyclin D:CDK4/6 complexes, it can no longer associate with and inhibit E2F1-3. Thus, CDK4/6-mediated phosphorylation of RB1 leads to transcriptional activation of E2F1-3 target genes needed for the S phase of the cell cycle (Connell-Crowley et al. 1997). CDK2, in complex with cyclin E, contributes to RB1 inactivation and also activates proteins needed for the initiation of DNA replication (Zhang 2007). Expression of D type cyclins is regulated by extracellular mitogens (Cheng et al. 1998, Depoortere et al. 1998). Catalytic activities of CDK4/6 and CDK2 are controlled by CDK inhibitors of the INK4 family (Serrano et al. 1993, Hannon and Beach 1994, Guan et al. 1994, Guan et al. 1996, Parry et al. 1995) and the Cip/Kip family, respectively.

References

Cobrinik D (2005). Pocket proteins and cell cycle control. Oncogene, 24, 2796-809.

Sadasivam S & DeCaprio JA (2013). The DREAM complex: master coordinator of cell cycle-dependent gene expression. Nat. Rev. Cancer, 13, 585-95.

Ferreira R, Magnaghi-Jaulin L, Robin P, Harel-Bellan A & Trouche D (1998). The three members of the pocket proteins family share the ability to repress E2F activity through recruitment of a histone deacetylase. Proc Natl Acad Sci U S A, 95, 10493-8.

Connell-Crowley L, Harper JW & Goodrich DW (1997). Cyclin D1/Cdk4 regulates retinoblastoma protein-mediated cell cycle arrest by site-specific phosphorylation. Mol Biol Cell, 8, 287-301.

Chellappan SP, Hiebert S, Mudryj M, Horowitz JM & Nevins JR (1991). The E2F transcription factor is a cellular target for the RB protein. Cell, 65, 1053-61.

Edit history

Date	Action	Author
2010-01-19	Edited	Matthews L
2010-01-20	Authored	Matthews L
2010-01-20	Created	Matthews L
2011-06-15	Reviewed	Grana X
2011-08-25	Reviewed	MacPherson D
2011-08-26	Revised	Orlic-Milacic M
2011-08-26	Authored	Orlic-Milacic M
2017-02-08	Edited	Orlic-Milacic M
2018-07-10	Reviewed	Manfredi JJ
2021-05-22	Modified	Shorser S

Entities found in this pathway (47)

Input	UniProt Id	Input	UniProt Id	Input	UniProt Id
CCNA2	P20248	CCNB1	P14635	CCNE1	P24864
CCNE2	O96020	CDC25A	P30304	CDC45	O75419
CDC6	Q99741	CDK1	P06493, P24941	CDK2	P24941
CDK4	P11802	CDKN2D	P55273	CDT1	Q9H211
CKS1B	P61024	DBF4	Q9UBU7	E2F1	O00716, Q01094
E2F2	Q14209, Q16254	FBXO5	Q9UKT4	GMNN	O75496
MCM10	Q7L590	MCM2	P33993, P49736	MCM5	P33992
MCM6	Q14566	MCM7	P33993	MYBL2	P10244
ORC1	Q13415	ORC6	Q9Y5N6	POLA2	Q14181
POLE2	P56282	PSMA2	P25787	PSMA4	P25789
PSMA6	P60900	PSMB3	P49720	PSMB4	P28070
PSMB7	P40306, Q99436	PSMC1	P62191	PSMC3	P17980
PSMC4	P43686	PSMC5	P62195	PSMD12	O00232
PSMD3	O43242	RBL2	Q08999	RPA3	P35244
RRM2	P31350	TFDP1	Q14186	TK1	P04183
TOP2A	P11388	TYMS	P04818		

Input	Ensembl Id	Input	Ensembl Id	Input	Ensembl Id
CCNA2	ENSG00000145386	CCNE1	ENSG00000105173	CDC25A	ENSG00000164045
CDC45	ENSG00000093009	CDC6	ENSG00000094804	CDK1	ENSG00000170312
CDT1	ENSG00000167513	E2F1	ENSG00000101412	FBXO5	ENSG00000112029
MYBL2	ENSG00000101057	ORC1	ENSG00000085840	RRM2	ENSG00000171848
TK1	ENSG00000167900	TOP2A	ENSG00000131747	TYMS	ENSG00000176890

8. Synthesis of DNA (R-HSA-69239)

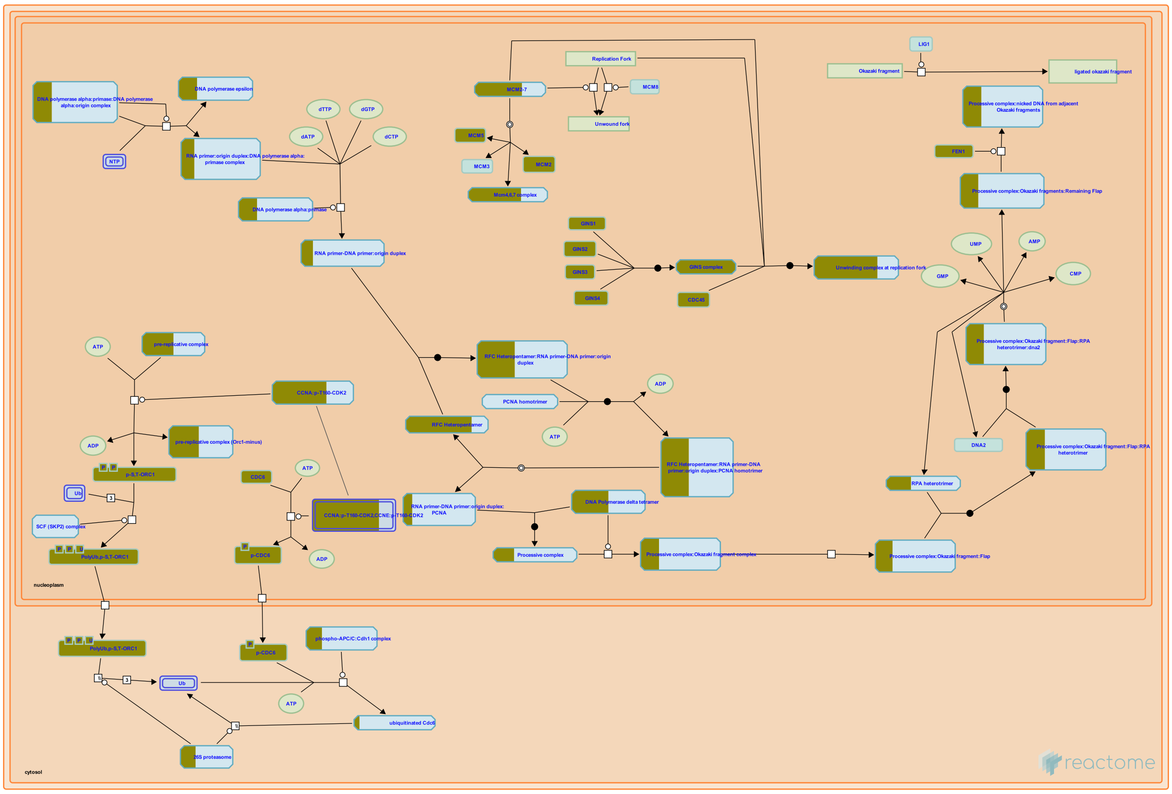

Cellular compartments: nucleoplasm, cytosol.

The actual synthesis of DNA occurs in the S phase of the cell cycle. This includes the initiation of DNA replication, when the first nucleotide of the new strand is laid down during the synthesis of the primer. The DNA replication preinitiation events begin in late M or early G1 phase.

References

Edit history

Date	Action	Author
2021-05-22	Modified	Shorser S

Entities found in this pathway (43)

Input	UniProt Id	Input	UniProt Id	Input	UniProt Id
ANAPC11	Q9NYG5	ANAPC15	P60006	CCNA2	P20248
CCNE1	P24864	CCNE2	O96020	CDC45	O75419
CDC6	Q99741	CDK1	P24941	CDK2	P24941
CDT1	Q9H211	FEN1	P39748	GINS1	Q14691
GINS2	Q9Y248	GINS3	Q9BRX5	GINS4	Q9BRT9
MCM2	P33993, P49736	MCM5	P33992	MCM6	Q14566
MCM7	P33993	ORC1	Q13415	ORC6	Q9Y5N6
POLA2	Q14181	POLD1	P28340	POLD2	P49005
POLE2	P56282	PSMA2	P25787	PSMA4	P25789
PSMA6	P60900	PSMB3	P49720	PSMB4	P28070
PSMB7	P40306, Q99436	PSMC1	P62191	PSMC3	P17980
PSMC4	P43686	PSMC5	P62195	PSMD12	O00232

Input	UniProt Id	Input	UniProt Id	Input	UniProt Id
PSMD3	O43242	RFC2	P35249, P35250	RFC4	P35249, P35250
RFC5	P40937, P40938	RPA3	P35244	UBE2C	O00762
UBE2S	Q16763				

9. Chromosome Maintenance (R-HSA-73886)

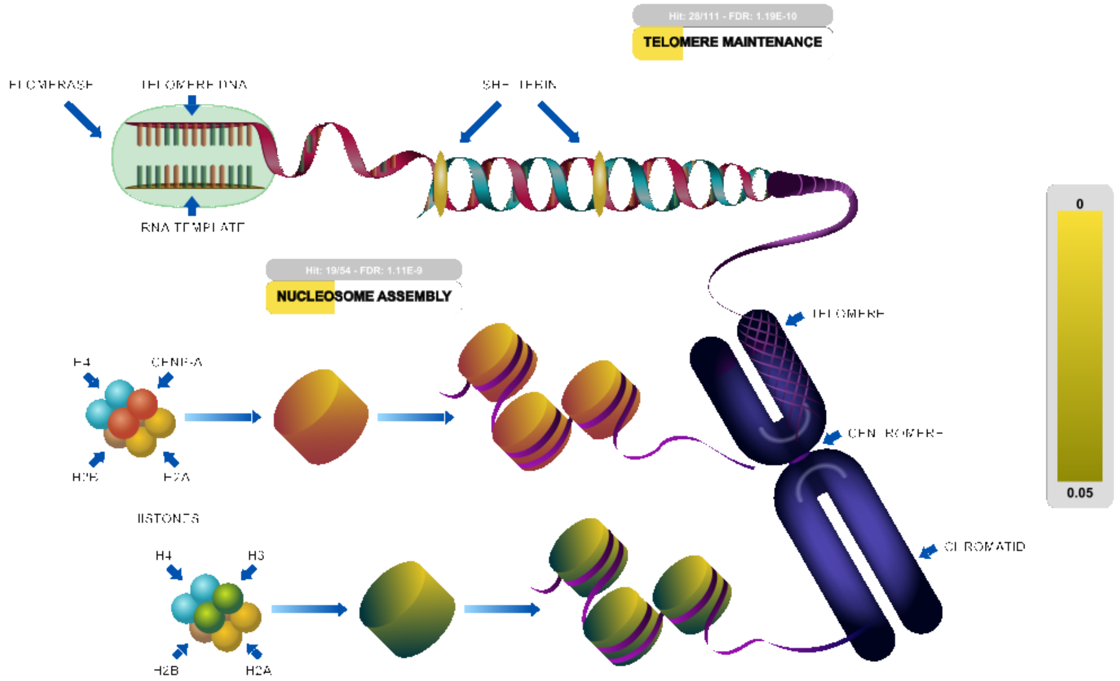

Cellular compartments: nucleoplasm, nuclear envelope.

Maintenance of chromosomal organization is critical for stable chromosome function. Two aspects of maintenance annotated in Reactome are centromeric chromatin assembly outside the context of DNA replication, involving **nucleosome assembly** with the histone H3 variant CenH3 (also called CENP-A), and the **maintenance of telomeres**, protein-DNA complexes at the ends of linear chromosomes that are important for genome stability.

References

Edit history

Date	Action	Author
2005-01-01	Created	Joshi-Tope G
2021-05-18	Edited	Joshi-Tope G
2021-05-18	Authored	Gillespie ME
2021-05-22	Modified	Shorser S

Entities found in this pathway (40)

Input	UniProt Id	Input	UniProt Id	Input	UniProt Id
BLM	P54132	CCNA2	P20248	CDK1	P24941
CDK2	P24941	CENPA	P49450	CENPH	Q9H3R5
CENPK	Q9BS16	CENPL	Q8N0S6	CENPM	Q9NSP4
CENPN	Q96H22	CENPO	Q9BU64	CENPU	Q71F23
CENPW	Q5EE01	CENPX	A8MT69	DSCC1	Q9BVC3
FEN1	P39748	H2AFX	P16104	H2AFZ	P0C0S5
HIST1H2AJ	Q99878	HIST1H2BH	P62807, Q93079	HIST2H2AC	Q16777

Input	UniProt Id	Input	UniProt Id	Input	UniProt Id
HJURP	Q8NCD3	MIS18A	Q9NYP9	NHP2	Q9NX24
OIP5	O43482	PIF1	Q9H611	POLA2	Q14181
POLD1	P28340	POLD2	P49005	POLR2D	O15514
POLR2F	P61218	POLR2I	P36954	POLR2J	P52435
RAD54L	P46100	RFC2	P35249, P35250	RFC4	P35249, P35250
RFC5	P40937, P40938	RPA3	P35244	RUVBL2	Q9Y230
WRAP53	Q9BUR4				

10. DNA Replication (R-HSA-69306)

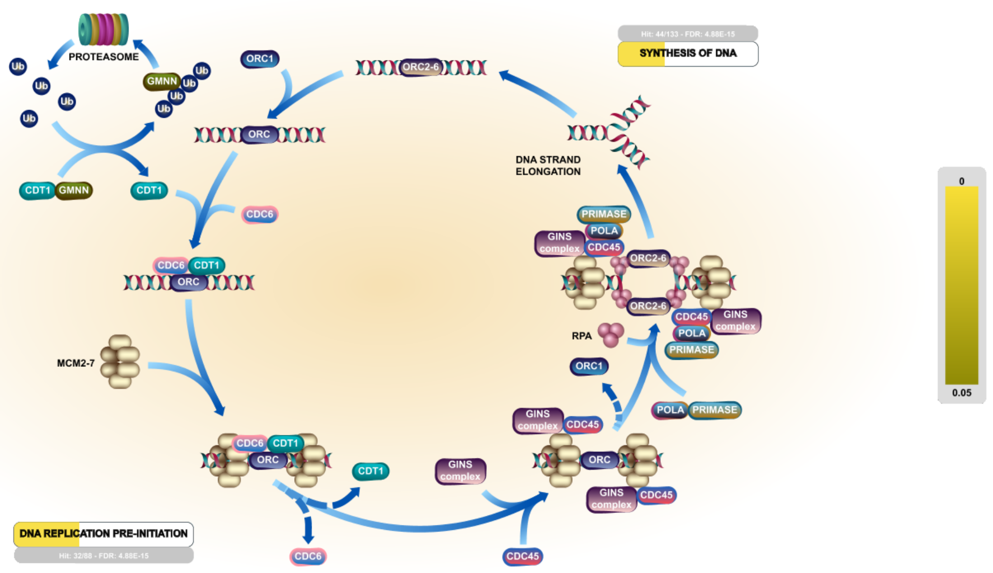

Cellular compartments: nucleoplasm, cytosol.

Studies in the past decade have suggested that the basic mechanism of DNA replication initiation is conserved in all kingdoms of life. Initiation in unicellular eukaryotes, in particular Saccharomyces cerevisiae (budding yeast), is well understood, and has served as a model for studies of DNA replication initiation in multicellular eukaryotes, including humans. In general terms, the first step of initiation is the binding of the replication initiator to the origin of replication. The replicative helicase is then assembled onto the origin, usually by a helicase assembly factor. Either shortly before or shortly after helicase assembly, some local unwinding of the origin of replication occurs in a region rich in adenine and thymine bases (often termed a DNA unwinding element, DUE). The unwound region provides the substrate for primer synthesis and initiation of DNA replication. The best-defined eukaryotic origins are those of S. cerevisiae, which have well-conserved sequence elements for initiator binding, DNA unwinding and binding of accessory proteins. In multicellular eukaryotes, unlike S. cerevisiae, these loci appear not to be defined by the presence of a DNA sequence motif. Indeed, choice of replication origins in a multicellular eukaryote may vary with developmental stage and tissue type. In cell-free models of metazoan DNA replication, such as the one provided by Xenopus egg extracts, there are only limited DNA sequence specificity requirements for replication initiation (Kelly & Brown 2000; Bell & Dutta 2002; Marahrens & Stillman 1992; Cimbora & Groudine 2001; Mahbubani et al 1992, Hyrien & Mechali 1993).

References

Marahrens Y & Stillman B (1992). A yeast chromosomal origin of DNA replication defined by multiple functional elements. Science, 255, 817-23.

Hyrien O & Méchali M (1993). Chromosomal replication initiates and terminates at random sequences but at regular intervals in the ribosomal DNA of Xenopus early embryos. EMBO J, 12, 4511-20.

Bell SP & Dutta A (2002). DNA replication in eukaryotic cells. Annu Rev Biochem, 71, 333-74.

Mahbubani HM, Paull T, Elder JK & Blow JJ (1992). DNA replication initiates at multiple sites on plasmid DNA in Xenopus egg extracts. Nucleic Acids Res, 20, 1457-62.

Kelly TJ & Brown GW (2000). Regulation of chromosome replication. Annu Rev Biochem, 69, 829-80.

Edit history

Date	Action	Author
2003-01-06	Authored	Catlett M, Davey MJ, Tye BK, O'Donnell M, Forsburg SL et al.
2003-01-06	Created	Catlett M, Davey MJ, Tye BK, O'Donnell M, Forsburg SL et al.
2005-09-07	Revised	Tye BK, Borowiec JA, Mendez J, Aladjem M
2021-05-18	Edited	Joshi-Tope G, Nickerson E, D'Eustachio P
2021-05-18	Reviewed	Mendez J, Aladjem M
2021-05-22	Modified	Shorser S

Entities found in this pathway (48)

Input	UniProt Id	Input	UniProt Id	Input	UniProt Id
ANAPC11	Q9NYG5	ANAPC15	P60006	CCNA2	P20248
CCNE1	P24864	CCNE2	O96020	CDC45	O75419
CDC6	Q99741	CDK1	P24941	CDK2	P24941
CDT1	Q9H211	DBF4	Q9UBU7	E2F1	O00716, Q01094
E2F2	Q14209	FEN1	P39748	GINS1	Q14691
GINS2	Q9Y248	GINS3	Q9BRX5	GINS4	Q9BRT9
GMNN	O75496	MCM10	Q7L590	MCM2	P33993, P49736
MCM5	P33992	MCM6	Q14566	MCM7	P33993
ORC1	Q13415	ORC6	Q9Y5N6	POLA2	Q14181
POLD1	P28340	POLD2	P49005	POLE2	P56282
PSMA2	P25787	PSMA4	P25789	PSMA6	P60900
PSMB3	P49720	PSMB4	P28070	PSMB7	P40306, Q99436
PSMC1	P62191	PSMC3	P17980	PSMC4	P43686
PSMC5	P62195	PSMD12	O00232	PSMD3	O43242
RFC2	P35249, P35250	RFC4	P35249, P35250	RFC5	P40937, P40938
RPA3	P35244	UBE2C	O00762	UBE2S	Q16763

11. Resolution of Sister Chromatid Cohesion (R-HSA-2500257)

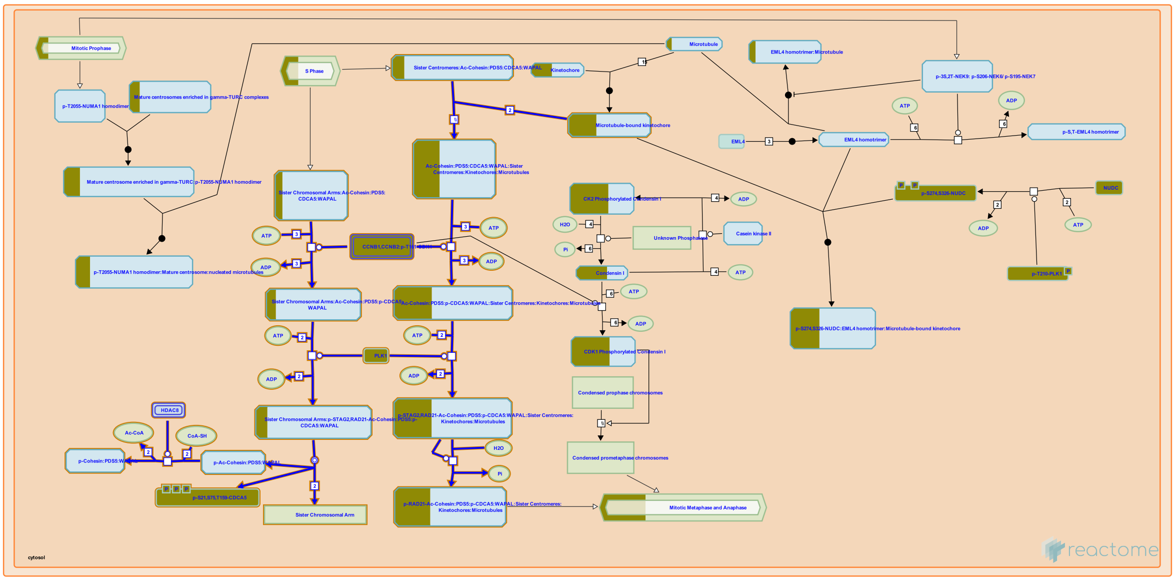

Cellular compartments: cytosol, chromosome, chromosome, centromeric region.

The resolution of sister chromatids in mitotic prometaphase involves removal of cohesin complexes from chromosomal arms, with preservation of cohesion at centromeres (Losada et al. 1998, Hauf et al. 2001, Hauf et al. 2005).

CDK1-mediated phosphorylation of cohesin-bound CDCA5 (Sororin) at threonine T159 provides a docking site for PLK1, enabling PLK1-mediated phosphorylation of cohesin subunits STAG2 (SA2) and RAD21 (Hauf et al. 2005, Dreier et al. 2011, Zhang et al. 2011). Further phosphorylation of CDCA5 by CDK1 results in dissociation of CDCA5 from cohesin complex, which restores the activity of WAPAL in removing STAG2-phosphorylated cohesin from chromosomal arms (Hauf et al. 2005, Gandhi et al. 2006, Kueng et al. 2006, Shintomi and Hirano 2006, Nishiyama et al. 2010, Zhang et al. 2011).

At centromeres, kinetochore proteins shugoshins (SGOL1 and SGOL2) enable PP2A-B56 (also a kinetochore constituent) to dephosphorylate the STAG2 subunit of centromeric cohesin. Dephosphorylation of STAG2 enables maintenance of centromeric cohesion, thus preventing separation of sister chromatids until anaphase (Salic et al. 2004, Kitajima et al. 2004, Kitajima et al. 2005, Kitajima et al. 2006).

References

Losada A, Hirano M & Hirano T (1998). Identification of Xenopus SMC protein complexes required for sister chromatid cohesion. Genes Dev., 12, 1986-97.

Hauf S, Waizenegger IC & Peters JM (2001). Cohesin cleavage by separase required for anaphase and cytokinesis in human cells. Science, 293, 1320-3.

Hauf S, Roitinger E, Koch B, Dittrich CM, Mechtler K & Peters JM (2005). Dissociation of cohesin from chromosome arms and loss of arm cohesion during early mitosis depends on phosphorylation of SA2. PLoS Biol, 3, e69.

Kitajima TS, Hauf S, Ohsugi M, Yamamoto T & Watanabe Y (2005). Human Bub1 defines the persistent cohesion site along the mitotic chromosome by affecting Shugoshin localization. Curr. Biol., 15, 353-9.

Kitajima TS, Sakuno T, Ishiguro K, Iemura S, Natsume T, Kawashima SA & Watanabe Y (2006). Shugoshin collaborates with protein phosphatase 2A to protect cohesin. Nature, 441, 46-52.

Edit history

Date	Action	Author
2012-10-01	Created	Orlic-Milacic M
2012-10-02	Authored	Orlic-Milacic M
2012-10-05	Edited	Matthews L, Gillespie ME
2012-10-22	Reviewed	Zhang N
2012-11-20	Reviewed	Tanno Y, Watanabe Y
2021-05-22	Modified	Shorser S

Entities found in this pathway (44)

Input	UniProt Id	Input	UniProt Id	Input	UniProt Id
AURKA	Q96GD4	AURKB	Q96GD4	BIRC5	O15392
BUB1	O43683, O60566	BUB1B	O60566	CCNB1	P14635
CCNB2	O95067	CDC20	Q12834	CDCA5	Q96FF9
CDCA8	Q53HL2	CDK1	P06493	CENPA	P49450
CENPE	Q02224	CENPF	P49454	CENPH	Q9H3R5
CENPK	Q9BS16	CENPL	Q8N0S6	CENPM	Q9NSP4
CENPN	Q96H22	CENPO	Q9BU64	CENPU	Q71F23
DSN1	Q9H410	ERCC6L	Q2NKX8	INCENP	Q9NQS7
KIF18A	Q8NI77	KIF2C	Q99661	MAD2L1	Q13257
NDC80	O14777	NUDC	Q9Y266	NUF2	Q9BZD4
NUP37	Q8NFH4	NUP85	Q9BW27	PLK1	P53350
RANBP2	P49792	RCC2	Q9P258	SGO1	Q5FBB7
SGO2	Q562F6	SKA1	Q96BD8	SKA2	Q8WVK7
SPC24	Q8NBT2	SPC25	Q9HBM1	TUBA1B	P68363
TUBA1C	Q9BQE3	ZWINT	O95229		

12. Separation of Sister Chromatids (R-HSA-2467813)

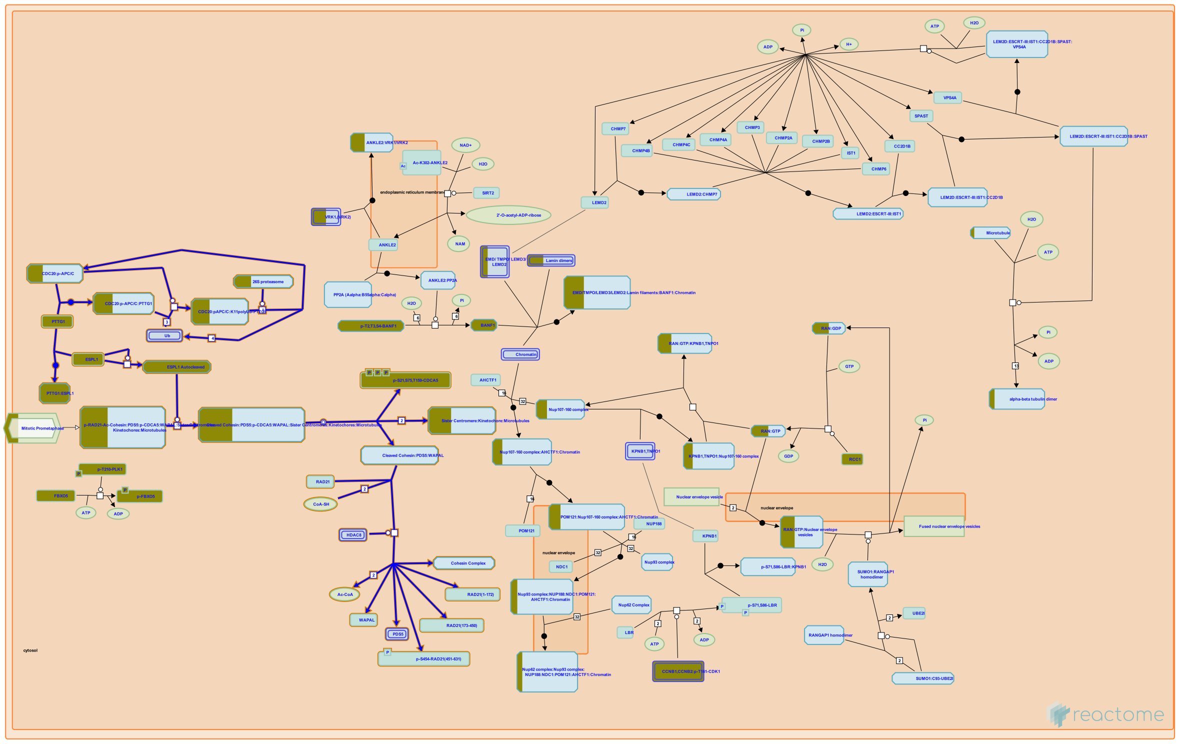

Cellular compartments: cytosol.

While sister chromatids resolve in prometaphase, separating along chromosomal arms, the cohesion of sister centromeres persists until anaphase. At the anaphase onset, the anaphase promoting complex/cyclosome (APC/C) ubiquitinates PTTG1 (securin), targeting it for degradation (Hagting et al. 2002). PTTG1 acts as an inhibitor of ESPL1 (known as separin i.e. separase). Hence, PTTG1 removal initiated by APC/C, enables ESPL1 to become catalytically active (Zou et al. 1999, Waizenegger et al. 2002). ESPL1 undergoes autoleavage (Waizenegger et al. 2002) and also cleaves RAD21 subunit of centromeric cohesin (Hauf et al. 2001). RAD21 cleavage promotes dissociation of cohesin complexes from sister centromeres, leading to separation of sister chromatids. Subsequent movement of sister chromatids to opposite poles of the mitotic spindle segregates replicated chromosomes to two daughter cells (Waizenegger et al. 2000, Hauf et al. 2001, Waizenegger et al. 2002).

References

Hauf S, Waizenegger IC & Peters JM (2001). Cohesin cleavage by separase required for anaphase and cytokinesis in human cells. Science, 293, 1320-3.

Hagting A, Den Elzen N, Vodermaier HC, Waizenegger IC, Peters JM & Pines J (2002). Human securin proteolysis is controlled by the spindle checkpoint and reveals when the APC/C switches from activation by Cdc20 to Cdh1. J Cell Biol, 157, 1125-37.

Waizenegger I, Giménez-Abián JF, Wernic D & Peters JM (2002). Regulation of human separase by securin binding and autocleavage. Curr. Biol., 12, 1368-78.

Waizenegger IC, Hauf S, Meinke A & Peters JM (2000). Two distinct pathways remove mammalian cohesin from chromosome arms in prophase and from centromeres in anaphase. Cell, 103, 399-410.

Zou H, McGarry TJ, Bernal T & Kirschner MW (1999). Identification of a vertebrate sister-chromatid separation inhibitor involved in transformation and tumorigenesis. Science, 285, 418-22.

Edit history

Date	Action	Author
2012-09-13	Created	Orlic-Milacic M
2012-10-02	Authored	Orlic-Milacic M
2012-10-05	Edited	Matthews L, Gillespie ME
2012-10-22	Reviewed	Zhang N
2012-11-20	Reviewed	Tanno Y, Watanabe Y
2021-05-31	Modified	Shorser S

Entities found in this pathway (59)

Input	UniProt Id	Input	UniProt Id	Input	UniProt Id
ANAPC11	Q9NYG5	ANAPC15	P60006	AURKA	Q96GD4
AURKB	Q96GD4	BIRC5	O15392	BUB1	O43683, O60566
BUB1B	O60566	CDC20	Q12834	CDCA5	Q96FF9
CDCA8	Q53HL2	CENPA	P49450	CENPE	Q02224
CENPF	P49454	CENPH	Q9H3R5	CENPK	Q9BS16
CENPL	Q8N0S6	CENPM	Q9NSP4	CENPN	Q96H22
CENPO	Q9BU64	CENPU	Q71F23	DSN1	Q9H410
ERCC6L	Q2NKX8	ESPL1	Q14674	INCENP	Q9NQS7
KIF18A	Q8NI77	KIF2C	Q99661	MAD2L1	Q13257
NDC80	O14777	NUDC	Q9Y266	NUF2	Q9BZD4
NUP37	Q8NFH4	NUP85	Q9BW27	PLK1	P53350
PSMA2	P25787	PSMA4	P25789	PSMA6	P60900
PSMB3	P49720	PSMB4	P28070	PSMB7	P40306, Q99436
PSMC1	P62191	PSMC3	P17980	PSMC4	P43686
PSMC5	P62195	PSMD12	O00232	PSMD3	O43242
PTTG1	O95997	RANBP2	P49792	RCC2	Q9P258
SGO1	Q5FBB7	SGO2	Q562F6	SKA1	Q96BD8
SKA2	Q8WVK7	SPC24	Q8NBT2	SPC25	Q9HBM1
TUBA1B	P68363	TUBA1C	Q9BQE3	UBE2C	O00762
UBE2S	Q16763	ZWINT	O95229		

13. S Phase (R-HSA-69242)

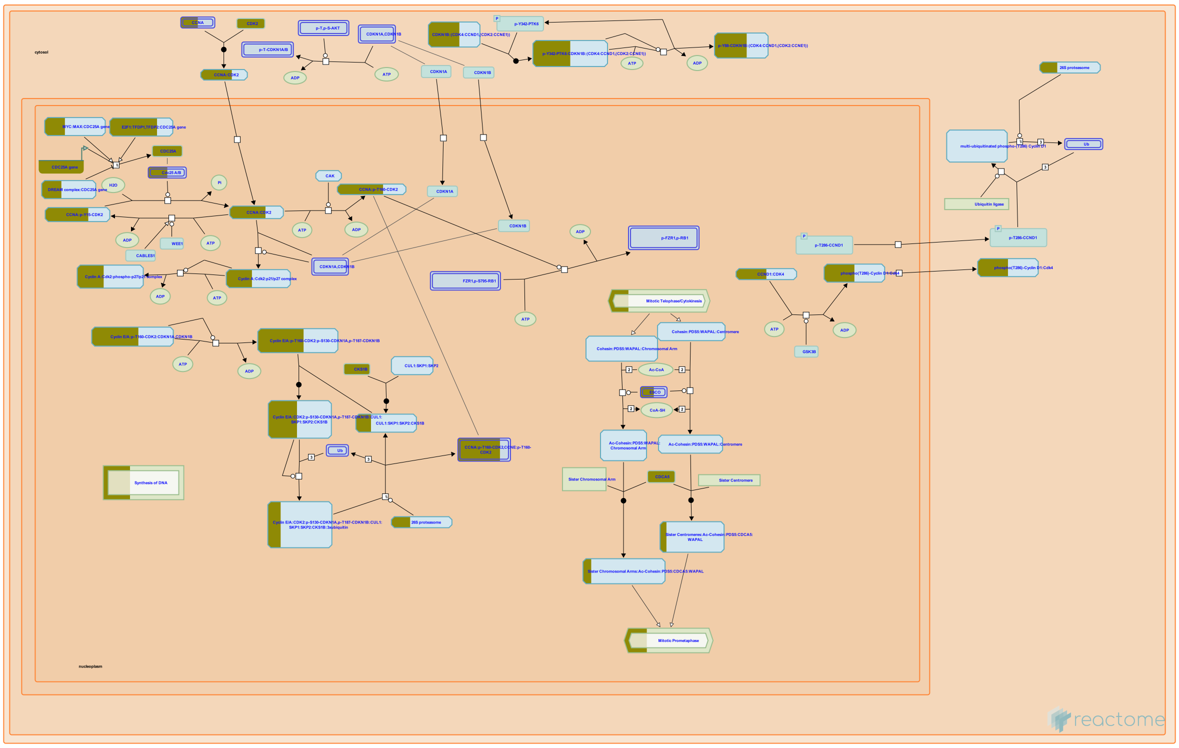

DNA synthesis occurs in the S phase, or the synthesis phase, of the cell cycle. The cell duplicates its hereditary material, and two copies of the chromosome are formed. As DNA replication continues, the E type cyclins shared by the G1 and S phases, are destroyed and the levels of the mitotic cyclins rise.

References

Edit history

Date	Action	Author
2018-07-10	Reviewed	Manfredi JJ
2021-05-22	Modified	Shorser S

Entities found in this pathway (52)

Input	UniProt Id	Input	UniProt Id	Input	UniProt Id
ANAPC11	Q9NYG5	ANAPC15	P60006	CCNA2	P20248
CCNE1	P24864	CCNE2	O96020	CDC25A	P30304
CDC45	O75419	CDC6	Q99741	CDCA5	Q96FF9
CDK1	P24941	CDK2	P24941	CDK4	P11802
CDT1	Q9H211	CKS1B	P61024	E2F1	Q01094
E2F2	Q16254	ESCO2	Q56NI9	FEN1	P39748
GINS1	Q14691	GINS2	Q9Y248	GINS3	Q9BRX5
GINS4	Q9BRT9	MCM2	P33993, P49736	MCM5	P33992
MCM6	Q14566	MCM7	P33993	ORC1	Q13415
ORC6	Q9Y5N6	POLA2	Q14181	POLD1	P28340
POLD2	P49005	POLE2	P56282	PSMA2	P25787
PSMA4	P25789	PSMA6	P60900	PSMB3	P49720

Input	UniProt Id	Input	UniProt Id	Input	UniProt Id
PSMB4	P28070	PSMB7	P40306, Q99436	PSMC1	P62191
PSMC3	P17980	PSMC4	P43686	PSMC5	P62195
PSMD12	O00232	PSMD3	O43242	RBL2	Q08999
RFC2	P35249, P35250	RFC4	P35249, P35250	RFC5	P40937, P40938
RPA3	P35244	TFDP1	Q14186	UBE2C	O00762
UBE2S	Q16763				

Input	Ensembl Id
CDC25A	ENSG00000164045

14. Cell Cycle, Mitotic (R-HSA-69278)

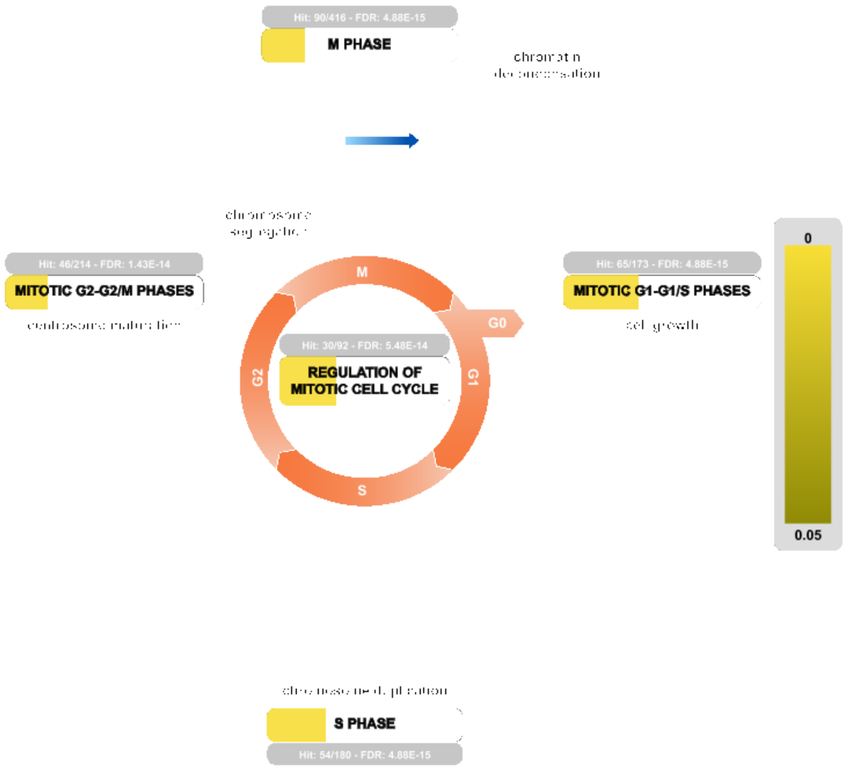

The events of replication of the genome and the subsequent segregation of chromosomes into daughter cells make up the cell cycle. DNA replication is carried out during a discrete temporal period known as the S (synthesis)-phase, and chromosome segregation occurs during a massive reorganization of cellular architecture at mitosis. Two gap-phases separate these cell cycle events: G1 between mitosis and S-phase, and G2 between S-phase and mitosis. Cells can exit the cell cycle for a period and enter a quiescent state known as G0, or terminally differentiate into cells that will not divide again, but undergo morphological development to carry out the wide variety of specialized functions of individual tissues.

A family of protein serine/threonine kinases known as the cyclin-dependent kinases (CDKs) controls progression through the cell cycle. As the name suggests, the kinase activity of the catalytic subunits is dependent on binding to cyclin partners, and control of cyclin abundance is one of several mechanisms by which CDK activity is regulated throughout the cell cycle.

A complex network of regulatory processes determines whether a quiescent cell (in G0 or early G1) will leave this state and initiate the processes to replicate its chromosomal DNA and divide. This regulation, during the **Mitotic G1-G1/S phases** of the cell cycle, centers on transcriptional regulation by the DREAM complex, with major roles for D and E type cyclin proteins.

Chromosomal DNA synthesis occurs in the **S phase**, or the synthesis phase, of the cell cycle. The cell duplicates its hereditary material, and two copies of each chromosome are formed. A key aspect of the **regulation of DNA** replication is the assembly and modification of a pre-replication complex assembled on ORC proteins.

Mitotic G2-G2/M phases encompass the interval between the completion of DNA synthesis and the beginning of mitosis. During G2, the cytoplasmic content of the cell increases. At G2/M transition, duplicated centrosomes mature and separate and CDK1:cyclin B complexes become active, setting the stage for spindle assembly and chromosome condensation at the start of mitotic **M phase**. Mitosis, or M phase, results in the generation of two daughter cells each with a complete diploid set of chromosomes. Events of the **M/G1 transition**, progression out of mitosis and division of the cell into two daughters (cytokinesis) are regulated by the Anaphase Promoting Complex.

The Anaphase Promoting Complex or Cyclosome (APC/C) plays additional roles in **regulation of the mitotic cell cycle**, insuring the appropriate length of the G1 phase. The APC/C itself is regulated by phosphorylation and interactions with checkpoint proteins.

References

Edit history

Date	Action	Author
2005-01-01	Authored	Walworth N, Bosco G, O'Donnell M
2005-01-01	Created	Walworth N, Bosco G, O'Donnell M
2010-01-19	Revised	Matthews L
2011-06-15	Reviewed	Grana X
2011-08-25	Reviewed	MacPherson D
2011-08-27	Revised	Orlic-Milacic M
2013-11-25	Edited	Matthews L, Gopinathrao G
2018-07-10	Reviewed	Manfredi JJ
2021-05-22	Modified	Shorser S

Entities found in this pathway (137)

Input	UniProt Id	Input	UniProt Id	Input	UniProt Id
ACTR1A	P61163	ANAPC11	Q9NYG5	ANAPC15	P60006
AURKA	O14965, Q96GD4	AURKB	O14965, Q96GD4	BANF1	O75531
BIRC5	O15392	BUB1	O43683, O60566	BUB1B	O60566
CCNA2	P20248	CCNB1	P14635	CCNB2	O95067
CCNE1	P24864	CCNE2	O96020	CDC20	Q12834
CDC25A	P30304	CDC25C	P30307	CDC45	O75419
CDC6	Q99741	CDCA5	Q96FF9	CDCA8	Q53HL2
CDK1	P06493, P24941	CDK2	P24941	CDK4	P11802
CDKN2D	P55273	CDT1	Q9H211	CENPA	P49450
CENPE	Q02224	CENPF	P49454	CENPH	Q9H3R5
CENPK	Q9BS16	CENPL	Q8N0S6	CENPM	Q9NSP4
CENPN	Q96H22	CENPO	Q9BU64	CENPU	Q71F23
CEP78	Q5JTW2	CKS1B	P61024	DBF4	Q9UBU7
DSN1	Q9H410	E2F1	O00716, Q01094	E2F2	Q14209, Q16254
ERCC6L	Q2NKX8	ESCO2	Q56NI9	ESPL1	Q14674
FBXO5	Q9UKT4	FEN1	P39748	FOXM1	Q08050
GINS1	Q14691	GINS2	Q9Y248	GINS3	Q9BRX5

Input	UniProt Id	Input	UniProt Id	Input	UniProt Id
GINS4	Q9BRT9	GMNN	O75496	GOLGA2	Q08379
GTSE1	Q9NYZ3	H2AFX	P16104	H2AFZ	P0C0S5
HAUS8	Q9BT25	HIST1H2AJ	Q99878	HIST1H2BH	P62807, Q93079
HIST1H3F	P68431	HIST2H2AC	Q16777	HMMR	O75330
INCENP	Q9NQS7	KIF18A	Q8NI77	KIF20A	O95235
KIF23	Q02241	KIF2C	Q99661	LMNB1	P20700
MAD2L1	Q13257	MCM10	Q7L590	MCM2	P33993, P49736
MCM5	P33992	MCM6	Q14566	MCM7	P33993
MYBL2	P10244	MZT2B	Q6NZ67	NCAPD2	Q15021
NCAPG	Q9BPX3	NCAPH	Q15003	NDC80	O14777
NEK2	P51955	NUDC	Q9Y266	NUF2	Q9BZD4
NUP37	Q8NFH4	NUP85	Q9BW27	ORC1	Q13415
ORC6	Q9Y5N6	PKMYT1	Q99640	PLK1	P53350
PLK4	O00444	POLA2	Q14181	POLD1	P28340
POLD2	P49005	POLE2	P56282	PSMA2	P25787
PSMA4	P25789	PSMA6	P60900	PSMB3	P49720
PSMB4	P28070	PSMB7	P40306, Q99436	PSMC1	P62191
PSMC3	P17980	PSMC4	P43686	PSMC5	P62195
PSMD12	O00232	PSMD3	O43242	PTTG1	O95997
RAN	P62826	RANBP2	P49792	RBL2	Q08999
RCC1	P18754	RCC2	Q9P258	RFC2	P35249, P35250
RFC4	P35249, P35250	RFC5	P40937, P40938	RPA3	P35244
RRM2	P31350	SGO1	Q5FBB7	SGO2	Q562F6
SKA1	Q96BD8	SKA2	Q8WVK7	SPC24	Q8NBT2
SPC25	Q9HBM1	TFDP1	Q14186	TK1	P04183
TMPO	P42167-1	TOP2A	P11388	TPX2	Q9ULW0
TUBA1B	P68363	TUBA1C	Q9BQE3	TUBG1	P23258, Q9NRH3
TYMS	P04818	UBE2C	O00762	UBE2S	Q16763
VRK1	Q99986	ZWINT	O95229		

Input	Ensembl Id	Input	Ensembl Id	Input	Ensembl Id
CCNA2	ENSG00000145386	CCNB1	ENSG00000134057	CCNB2	ENSG00000157456
CCNE1	ENSG00000105173	CDC25A	ENSG00000164045	CDC45	ENSG00000093009
CDC6	ENSG00000094804	CDK1	ENSG00000170312	CDT1	ENSG00000167513
CENPF	ENSG00000117724	E2F1	ENSG00000101412	FBXO5	ENSG00000112029
MYBL2	ENSG00000101057	ORC1	ENSG00000085840	PLK1	ENSG00000166851
RRM2	ENSG00000171848	TK1	ENSG00000167900	TOP2A	ENSG00000131747
TYMS	ENSG00000176890				

15. DNA Replication Pre-Initiation (R-HSA-69002)

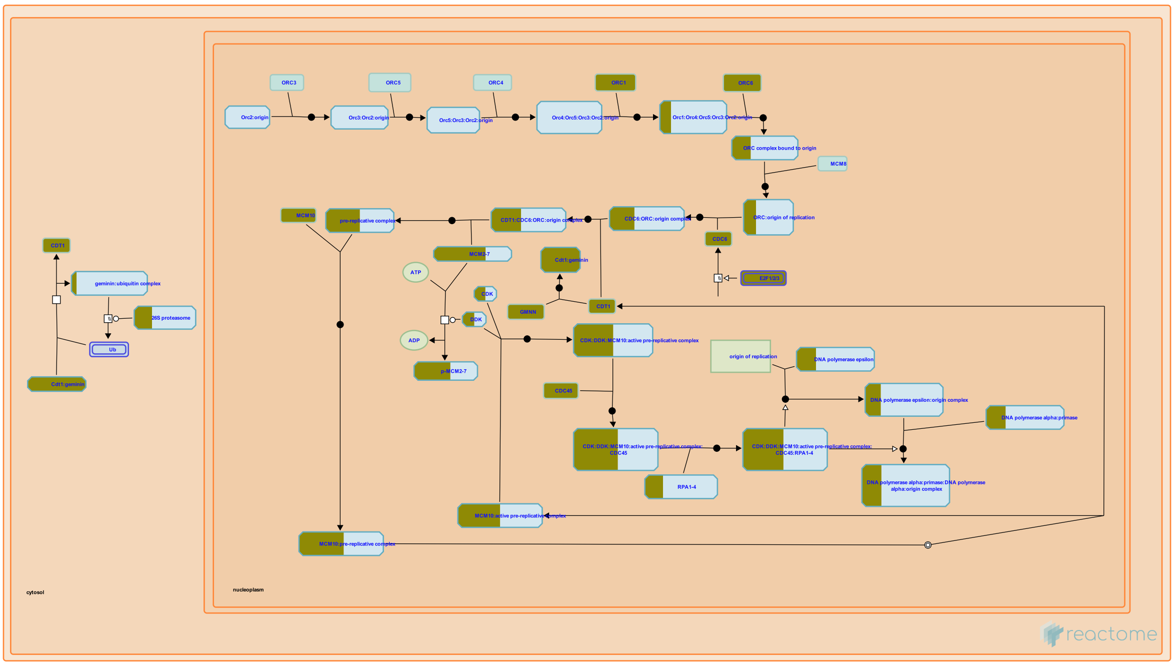

Cellular compartments: nucleoplasm, cytosol.

Although, DNA replication occurs in the S phase of the cell cycle, the formation of the DNA replication pre-initiation complex begins during G1 phase.

References

Edit history

Date	Action	Author
2003-06-05	Created	Davey MJ, O'Donnell M
2006-03-17	Authored	Davey MJ, Tye BK, O'Donnell M
2021-05-18	Edited	Joshi-Tope G
2021-05-22	Modified	Shorser S

Entities found in this pathway (30)

Input	UniProt Id	Input	UniProt Id	Input	UniProt Id
CDC45	O75419	CDC6	Q99741	CDK2	P24941
CDT1	Q9H211	DBF4	Q9UBU7	E2F1	O00716, Q01094
E2F2	Q14209	GMNN	O75496	MCM10	Q7L590
MCM2	P33993, P49736	MCM5	P33992	MCM6	Q14566
MCM7	P33993	ORC1	Q13415	ORC6	Q9Y5N6
POLA2	Q14181	POLE2	P56282	PSMA2	P25787
PSMA4	P25789	PSMA6	P60900	PSMB3	P49720
PSMB4	P28070	PSMB7	P40306, Q99436	PSMC1	P62191
PSMC3	P17980	PSMC4	P43686	PSMC5	P62195
PSMD12	O00232	PSMD3	O43242	RPA3	P35244

16. Mitotic Prometaphase (R-HSA-68877)

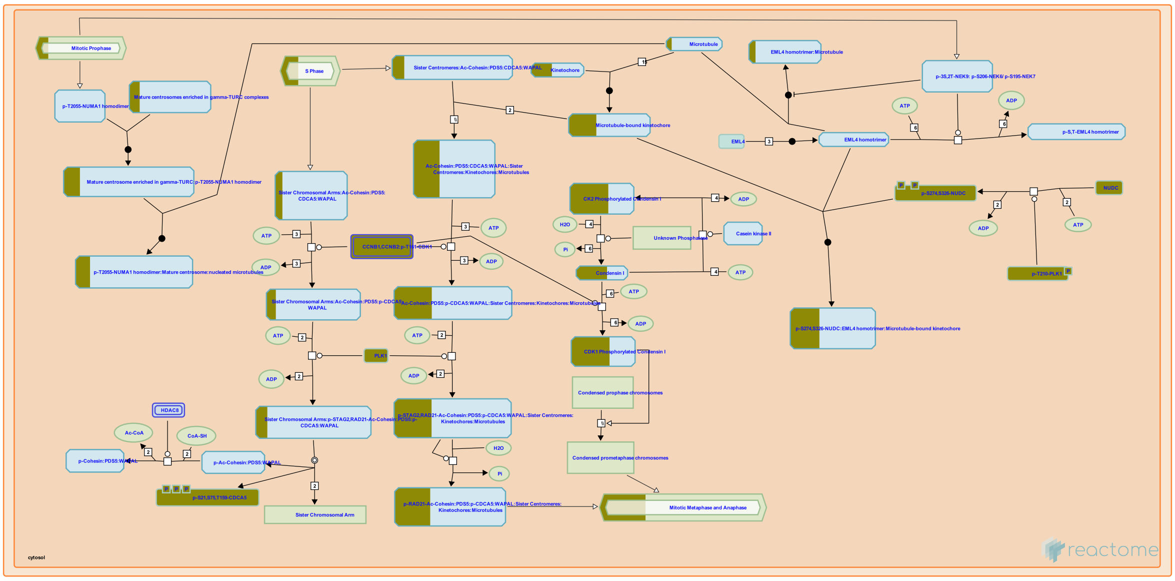

The dissolution of the nuclear membrane marks the beginning of the prometaphase. Kinetochores are created when proteins attach to the centromeres. Microtubules then attach at the kinetochores, and the chromosomes begin to move to the metaphase plate.

References

Edit history

Date	Action	Author
2021-05-22	Modified	Shorser S

Entities found in this pathway (54)

Input	UniProt Id	Input	UniProt Id	Input	UniProt Id
ACTR1A	P61163	AURKA	Q96GD4	AURKB	Q96GD4
BIRC5	O15392	BUB1	O43683, O60566	BUB1B	O60566
CCNB1	P14635	CCNB2	O95067	CDC20	Q12834
CDCA5	Q96FF9	CDCA8	Q53HL2	CDK1	P06493
CENPA	P49450	CENPE	Q02224	CENPF	P49454
CENPH	Q9H3R5	CENPK	Q9BS16	CENPL	Q8N0S6
CENPM	Q9NSP4	CENPN	Q96H22	CENPO	Q9BU64
CENPU	Q71F23	CEP78	Q5JTW2	DSN1	Q9H410
ERCC6L	Q2NKX8	HAUS8	Q9BT25	INCENP	Q9NQS7
KIF18A	Q8NI77	KIF2C	Q99661	MAD2L1	Q13257
MZT2B	Q6NZ67	NCAPD2	Q15021	NCAPG	Q9BPX3
NCAPH	Q15003	NDC80	O14777	NEK2	P51955
NUDC	Q9Y266	NUF2	Q9BZD4	NUP37	Q8NFH4
NUP85	Q9BW27	PLK1	P53350	PLK4	O00444
RANBP2	P49792	RCC2	Q9P258	SGO1	Q5FBB7
SGO2	Q562F6	SKA1	Q96BD8	SKA2	Q8WVK7
SPC24	Q8NBT2	SPC25	Q9HBM1	TUBA1B	P68363
TUBA1C	Q9BQE3	TUBG1	P23258, Q9NRH3	ZWINT	O95229

17. Cell Cycle (R-HSA-1640170)

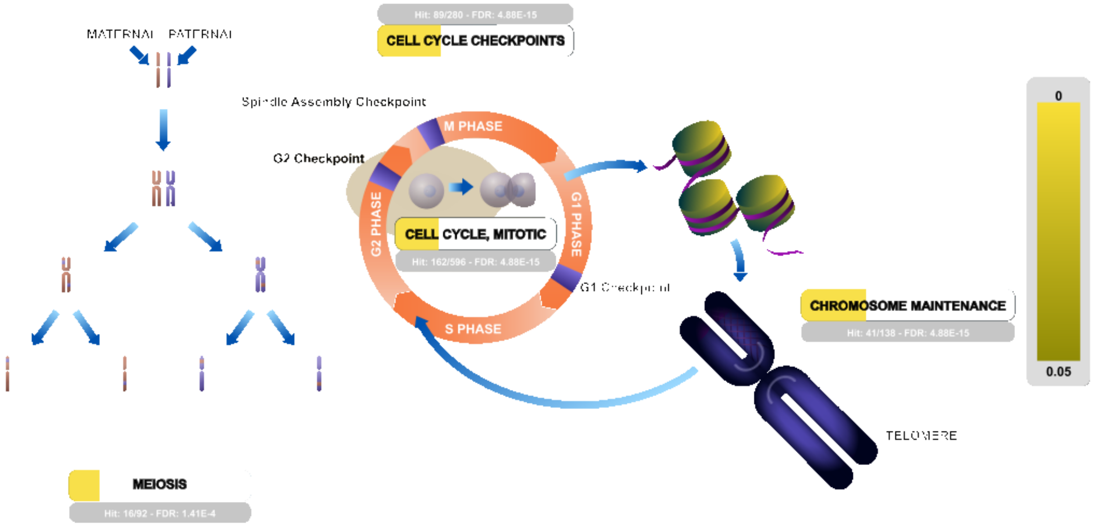

The replication of the genome and the subsequent segregation of chromosomes into daughter cells are controlled by a series of events collectively known as the **cell cycle**. DNA replication is carried out during a discrete temporal period known as the S (synthesis)-phase, and chromosome segregation occurs during a massive reorganization to cellular architecture at mitosis. Two gap-phases separate these major cell cycle events: G1 between mitosis and S-phase, and G2 between S-phase and mitosis. In the development of the human body, cells can exit the cell cycle for a period and enter a quiescent state known as G0, or terminally differentiate into cells that will not divide again, but undergo morphological development to carry out the wide variety of specialized functions of individual tissues.

A family of protein serine/threonine kinases known as the cyclin-dependent kinases (CDKs) controls progression through the cell cycle. As the name suggests, the activity of the catalytic subunit is dependent on binding to a cyclin partner. The human genome encodes several cyclins and several CDKs, with their names largely derived from the order in which they were identified. The oscillation of cyclin abundance is one important mechanism by which these enzymes phosphorylate key substrates to promote events at the relevant time and place. Additional post-translational modifications and interactions with regulatory proteins ensure that CDK activity is precisely regulated, frequently confined to a narrow window of activity.

In addition, genome integrity in the cell cycle is maintained by the action of a number of signal transduction pathways, known as **cell cycle checkpoints**, which monitor the accuracy and completeness of DNA replication during S phase and the orderly chromosomal condensation, pairing and partition into daughter cells during mitosis.

Replication of telomeric DNA at the ends of human chromosomes and packaging of their centromeres into chromatin are two aspects of **chromosome maintenance** that are integral parts of the cell cycle.

Meiosis is the specialized form of cell division that generates haploid gametes from diploid germ cells, associated with recombination (exchange of genetic material between chromosomal homologs).

References

Edit history

Date	Action	Author
2011-10-10	Edited	Matthews L
2011-10-10	Created	Matthews L
2021-05-22	Modified	Shorser S

Entities found in this pathway (162)

Input	UniProt Id	Input	UniProt Id	Input	UniProt Id
ACTR1A	P61163	ANAPC11	Q9NYG5	ANAPC15	P60006
AURKA	O14965, Q96GD4	AURKB	O14965, Q96GD4	BANF1	O75531
BIRC5	O15392	BLM	P54132	BRIP1	Q9BX63
BUB1	O43683, O60566	BUB1B	O60566	CCNA2	P20248
CCNB1	P14635	CCNB2	O95067	CCNE1	P24864
CCNE2	O96020	CDC20	Q12834	CDC25A	P30304
CDC25C	P30307	CDC45	O75419	CDC6	Q99741
CDCA5	Q96FF9	CDCA8	Q53HL2	CDK1	P06493, P24941
CDK2	P24941	CDK4	P11802	CDKN2D	P55273
CDT1	Q9H211	CENPA	P49450	CENPE	Q02224
CENPF	P49454	CENPH	Q9H3R5	CENPK	Q9BS16
CENPL	Q8N0S6	CENPM	Q9NSP4	CENPN	Q96H22
CENPO	Q9BU64	CENPU	Q71F23	CENPW	Q5EE01
CENPX	A8MT69	CEP78	Q5JTW2	CHEK1	O14757
CHEK2	O96017	CKS1B	P61024	DBF4	Q9UBU7
DSCC1	Q9BVC3	DSN1	Q9H410	E2F1	O00716, Q01094
E2F2	Q14209, Q16254	ERCC6L	Q2NKX8	ESCO2	Q56NI9
ESPL1	Q14674	EXO1	Q9UQ84	FBXO5	Q9UKT4
FEN1	P39748	FOXM1	Q08050	GINS1	Q14691
GINS2	Q9Y248	GINS3	Q9BRX5	GINS4	Q9BRT9
GMNN	O75496	GOLGA2	Q08379	GTSE1	Q9NYZ3
H2AFX	P16104	H2AFZ	P0C0S5	HAUS8	Q9BT25
HIST1H2AJ	Q99878	HIST1H2BH	P62807, Q93079	HIST1H3F	P68431
HIST2H2AC	Q16777	HJURP	Q8NCD3	HMMR	O75330
INCENP	Q9NQS7	KIF18A	Q8NI77	KIF20A	O95235
KIF23	Q02241	KIF2C	Q99661	LMNB1	P20700
MAD2L1	Q13257	MCM10	Q7L590	MCM2	P33993, P49736
MCM5	P33992	MCM6	Q14566	MCM7	P33993
MIS18A	Q9NYP9	MND1	Q9BWT6	MYBL2	P10244
MZT2B	Q6NZ67	NCAPD2	Q15021	NCAPG	Q9BPX3
NCAPH	Q15003	NDC80	O14777	NEK2	P51955
NHP2	Q9NX24	NUDC	Q9Y266	NUF2	Q9BZD4
NUP37	Q8NFH4	NUP85	Q9BW27	OIP5	O43482
ORC1	Q13415	ORC6	Q9Y5N6	PIF1	Q9H611
PKMYT1	Q99640	PLK1	P53350	PLK4	O00444
POLA2	Q14181	POLD1	P28340	POLD2	P49005
POLE2	P56282	POLR2D	O15514	POLR2F	P61218
POLR2I	P36954	POLR2J	P52435	PSMA2	P25787

Input	UniProt Id	Input	UniProt Id	Input	UniProt Id
PSMA4	P25789	PSMA6	P60900	PSMB3	P49720
PSMB4	P28070	PSMB7	P40306, Q99436	PSMC1	P62191
PSMC3	P17980	PSMC3IP	Q9P2W1	PSMC4	P43686
PSMC5	P62195	PSMD12	O00232	PSMD3	O43242
PTTG1	O95997	RAD51	Q06609	RAD54L	P46100
RAN	P62826	RANBP2	P49792	RBL2	Q08999
RCC1	P18754	RCC2	Q9P258	RFC2	P35249, P35250
RFC4	P35249, P35250	RFC5	P40937, P40938	RMI2	Q96E14
RPA3	P35244	RRM2	P31350	RUVBL2	Q9Y230
SGO1	Q5FBB7	SGO2	Q562F6	SKA1	Q96BD8
SKA2	Q8WVK7	SPC24	Q8NBT2	SPC25	Q9HBM1
SYCE2	Q6PIF2	TFDP1	Q14186	TK1	P04183
TMPO	P42167-1	TOP2A	P11388	TPX2	Q9ULW0
TUBA1B	P68363	TUBA1C	Q9BQE3	TUBG1	P23258, Q9NRH3
TYMS	P04818	UBE2C	O00762	UBE2S	Q16763
VRK1	Q99986	WRAP53	Q9BUR4	ZWINT	O95229

Input	Ensembl Id	Input	Ensembl Id	Input	Ensembl Id
CCNA2	ENSG00000145386	CCNB1	ENSG00000134057	CCNB2	ENSG00000157456
CCNE1	ENSG00000105173	CDC25A	ENSG00000164045	CDC45	ENSG00000093009
CDC6	ENSG00000094804	CDK1	ENSG00000170312	CDT1	ENSG00000167513
CENPF	ENSG00000117724	E2F1	ENSG00000101412	FBXO5	ENSG00000112029
MYBL2	ENSG00000101057	ORC1	ENSG00000085840	PLK1	ENSG00000166851
RRM2	ENSG00000171848	TK1	ENSG00000167900	TOP2A	ENSG00000131747
TYMS	ENSG00000176890				

18. M Phase (R-HSA-68886)

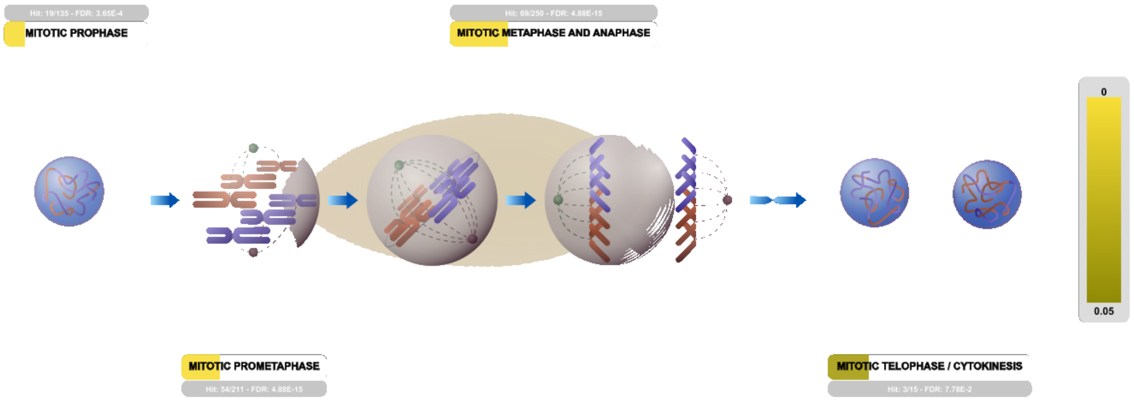

Mitosis, or the M phase, involves nuclear division and cytokinesis, where two identical daughter cells are produced. Mitosis involves prophase, prometaphase, metaphase, anaphase, and telophase. Finally, cytokinesis leads to cell division. The phase between two M phases is called the interphase; it encompasses the G1, S, and G2 phases of the cell cycle.

References

Edit history

Date	Action	Author
2018-07-10	Reviewed	Manfredi JJ
2021-05-22	Modified	Shorser S

Entities found in this pathway (88)

Input	UniProt Id	Input	UniProt Id	Input	UniProt Id
ACTR1A	P61163	ANAPC11	Q9NYG5	ANAPC15	P60006
AURKA	Q96GD4	AURKB	Q96GD4	BANF1	O75531
BIRC5	O15392	BUB1	O43683, O60566	BUB1B	O60566
CCNB1	P14635	CCNB2	O95067	CDC20	Q12834
CDCA5	Q96FF9	CDCA8	Q53HL2	CDK1	P06493
CENPA	P49450	CENPE	Q02224	CENPF	P49454
CENPH	Q9H3R5	CENPK	Q9BS16	CENPL	Q8N0S6
CENPM	Q9NSP4	CENPN	Q96H22	CENPO	Q9BU64
CENPU	Q71F23	CEP78	Q5JTW2	DSN1	Q9H410
ERCC6L	Q2NKX8	ESPL1	Q14674	FBXO5	Q9UKT4
GOLGA2	Q08379	H2AFX	P16104	H2AFZ	P0C0S5
HAUS8	Q9BT25	HIST1H2AJ	Q99878	HIST1H2BH	P62807, Q93079
HIST1H3F	P68431	HIST2H2AC	Q16777	INCENP	Q9NQS7
KIF18A	Q8NI77	KIF20A	O95235	KIF23	Q02241
KIF2C	Q99661	LMNB1	P20700	MAD2L1	Q13257
MZT2B	Q6NZ67	NCAPD2	Q15021	NCAPG	Q9BPX3
NCAPH	Q15003	NDC80	O14777	NEK2	P51955
NUDC	Q9Y266	NUF2	Q9BZD4	NUP37	Q8NFH4
NUP85	Q9BW27	PLK1	P53350	PLK4	O00444

Input	UniProt Id	Input	UniProt Id	Input	UniProt Id
PSMA2	P25787	PSMA4	P25789	PSMA6	P60900
PSMB3	P49720	PSMB4	P28070	PSMB7	P40306, Q99436
PSMC1	P62191	PSMC3	P17980	PSMC4	P43686
PSMC5	P62195	PSMD12	O00232	PSMD3	O43242
PTTG1	O95997	RAN	P62826	RANBP2	P49792
RCC1	P18754	RCC2	Q9P258	SGO1	Q5FBB7
SGO2	Q562F6	SKA1	Q96BD8	SKA2	Q8WVK7
SPC24	Q8NBT2	SPC25	Q9HBM1	TMPO	P42167-1
TUBA1B	P68363	TUBA1C	Q9BQE3	TUBG1	P23258, Q9NRH3
UBE2C	O00762	UBE2S	Q16763	VRK1	Q99986
ZWINT	O95229				

19. Cell Cycle Checkpoints (R-HSA-69620)

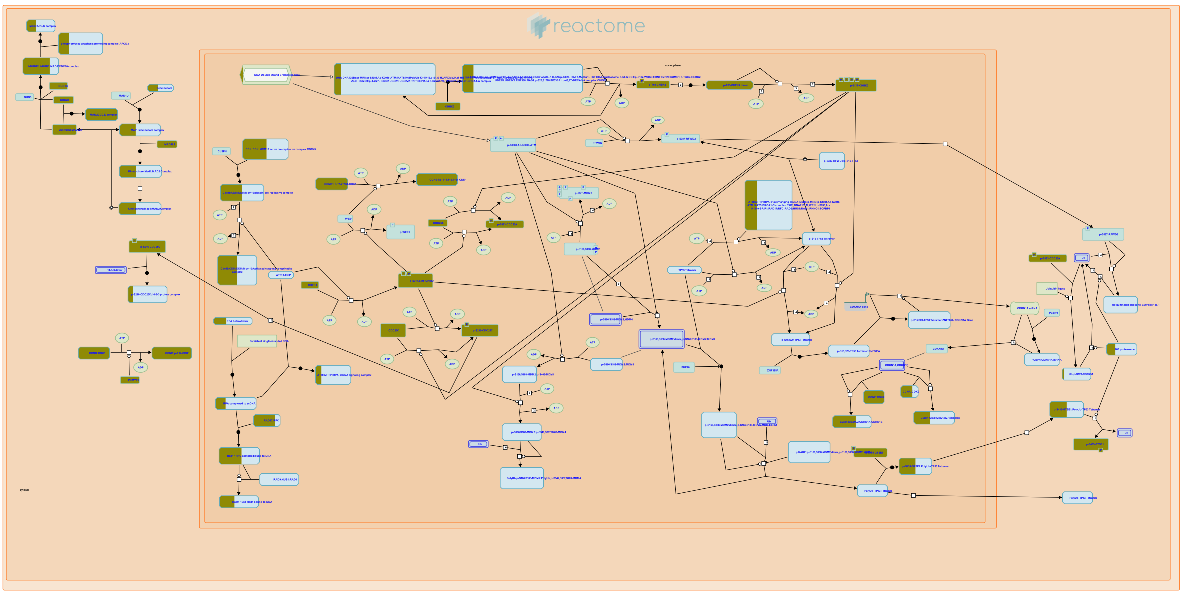

A hallmark of the human cell cycle in normal somatic cells is its precision. This remarkable fidelity is achieved by a number of signal transduction pathways, known as checkpoints, which monitor cell cycle progression ensuring an interdependency of S-phase and mitosis, the integrity of the genome and the fidelity of chromosome segregation.

Checkpoints are layers of control that act to delay CDK activation when defects in the division program occur. As the CDKs functioning at different points in the cell cycle are regulated by different means, the various checkpoints differ in the biochemical mechanisms by which they elicit their effect. However, all checkpoints share a common hierarchy of a sensor, signal transducers, and effectors that interact with the CDKs.

The stability of the genome in somatic cells contrasts to the almost universal genomic instability of tumor cells. There are a number of documented genetic lesions in checkpoint genes, or in cell cycle genes themselves, which result either directly in cancer or in a predisposition to certain cancer types. Indeed, restraint over cell cycle progression and failure to monitor genome integrity are likely prerequisites for the molecular evolution required for the development of a tumor. Perhaps most notable amongst these is the p53 tumor suppressor gene, which is mutated in >50% of human tumors. Thus, the importance of the checkpoint pathways to human biology is clear.

References

Edit history

Date	Action	Author
2005-01-01	Authored	Walworth N, Hoffmann I, Yen TJ, O'Donnell M, Khanna KK
2005-01-01	Created	Walworth N, Hoffmann I, Yen TJ, O'Donnell M, Khanna KK
2013-11-25	Edited	Matthews L
2021-05-18	Reviewed	Sanchez Y, Knudsen E, Hardwick KG
2021-05-22	Modified	Shorser S

Entities found in this pathway (87)

Input	UniProt Id	Input	UniProt Id	Input	UniProt Id
ANAPC11	Q9NYG5	ANAPC15	P60006	AURKA	Q96GD4
AURKB	Q96GD4	BIRC5	O15392	BLM	P54132
BRIP1	Q9BX63	BUB1	O43683, O60566	BUB1B	O60566
CCNA2	P20248	CCNB1	P14635	CCNB2	O95067
CCNE1	P24864	CCNE2	O96020	CDC20	Q12834
CDC25A	P30304	CDC25C	P30307	CDC45	O75419
CDC6	Q99741	CDCA8	Q53HL2	CDK1	P06493
CDK2	P24941	CENPA	P49450	CENPE	Q02224
CENPF	P49454	CENPH	Q9H3R5	CENPK	Q9BS16
CENPL	Q8N0S6	CENPM	Q9NSP4	CENPN	Q96H22
CENPO	Q9BU64	CENPU	Q71F23	CHEK1	O14757
CHEK2	O96017	DBF4	Q9UBU7	DSN1	Q9H410
ERCC6L	Q2NKX8	EXO1	Q9UQ84	GTSE1	Q9NYZ3
H2AFX	P16104	HIST1H2BH	P62807, Q93079	INCENP	Q9NQS7
KIF18A	Q8NI77	KIF2C	Q99661	MAD2L1	Q13257
MCM10	Q7L590	MCM2	P33993, P49736	MCM5	P33992
MCM6	Q14566	MCM7	P33993	NDC80	O14777
NUDC	Q9Y266	NUF2	Q9BZD4	NUP37	Q8NFH4
NUP85	Q9BW27	ORC1	Q13415	ORC6	Q9Y5N6
PKMYT1	Q99640	PLK1	P53350	PSMA2	P25787
PSMA4	P25789	PSMA6	P60900	PSMB3	P49720
PSMB4	P28070	PSMB7	P40306, Q99436	PSMC1	P62191
PSMC3	P17980	PSMC4	P43686	PSMC5	P62195
PSMD12	O00232	PSMD3	O43242	RANBP2	P49792
RCC2	Q9P258	RFC2	P35249, P35250	RFC4	P35249, P35250
RFC5	P40937, P40938	RMI2	Q96E14	RPA3	P35244
SGO1	Q5FBB7	SGO2	Q562F6	SKA1	Q96BD8
SKA2	Q8WVK7	SPC24	Q8NBT2	SPC25	Q9HBM1
UBE2C	O00762	UBE2S	Q16763	ZWINT	O95229

20. Mitotic Metaphase and Anaphase (R-HSA-2555396)

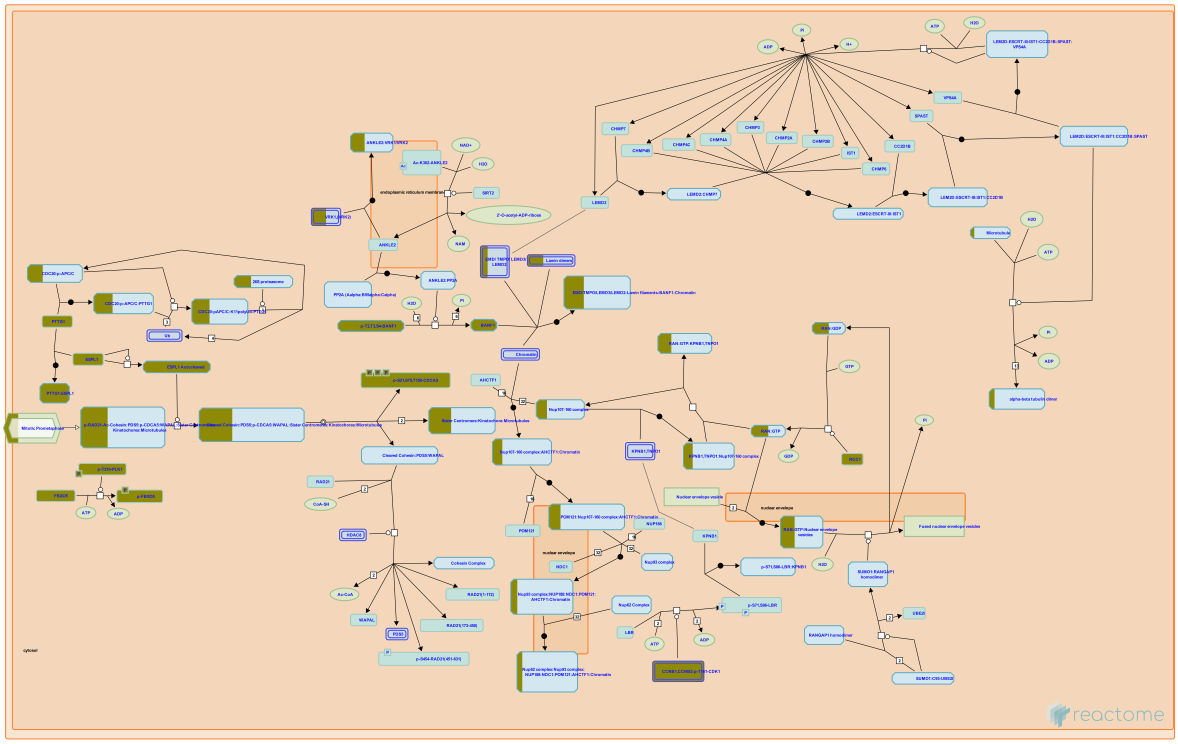

Cellular compartments: cytosol.

Metaphase is marked by the formation of the metaphase plate. The metaphase plate is formed when the spindle fibers align the chromosomes along the middle of the cell. Such an organization helps to ensure that later, when the chromosomes are separated, each new nucleus that is formed receives one copy of each chromosome. This pathway has not yet been annotated in Reactome.

The metaphase to anaphase transition during mitosis is triggered by the destruction of mitotic cyclins.

In anaphase, the paired chromosomes separate at the centromeres, and move to the opposite sides of the cell. The movement of the chromosomes is facilitated by a combination of kinetochore movement along the spindle microtubules and through the physical interaction of polar microtubules.

References

Edit history

Date	Action	Author
2012-11-02	Created	Orlic-Milacic M
2021-05-22	Modified	Shorser S

Entities found in this pathway (69)

Input	UniProt Id	Input	UniProt Id	Input	UniProt Id
ANAPC11	Q9NYG5	ANAPC15	P60006	AURKA	Q96GD4
AURKB	Q96GD4	BANF1	O75531	BIRC5	O15392
BUB1	O43683, O60566	BUB1B	O60566	CCNB1	P14635

Input	UniProt Id	Input	UniProt Id	Input	UniProt Id
CCNB2	O95067	CDC20	Q12834	CDCA5	Q96FF9
CDCA8	Q53HL2	CDK1	P06493	CENPA	P49450
CENPE	Q02224	CENPF	P49454	CENPH	Q9H3R5
CENPK	Q9BS16	CENPL	Q8N0S6	CENPM	Q9NSP4
CENPN	Q96H22	CENPO	Q9BU64	CENPU	Q71F23
DSN1	Q9H410	ERCC6L	Q2NKX8	ESPL1	Q14674
FBXO5	Q9UKT4	INCENP	Q9NQS7	KIF18A	Q8NI77
KIF2C	Q99661	LMNB1	P20700	MAD2L1	Q13257
NDC80	O14777	NUDC	Q9Y266	NUF2	Q9BZD4
NUP37	Q8NFH4	NUP85	Q9BW27	PLK1	P53350
PSMA2	P25787	PSMA4	P25789	PSMA6	P60900
PSMB3	P49720	PSMB4	P28070	PSMB7	P40306, Q99436
PSMC1	P62191	PSMC3	P17980	PSMC4	P43686
PSMC5	P62195	PSMD12	O00232	PSMD3	O43242
PTTG1	O95997	RAN	P62826	RANBP2	P49792
RCC1	P18754	RCC2	Q9P258	SGO1	Q5FBB7
SGO2	Q562F6	SKA1	Q96BD8	SKA2	Q8WVK7
SPC24	Q8NBT2	SPC25	Q9HBM1	TMPO	P42167-1
TUBA1B	P68363	TUBA1C	Q9BQE3	UBE2C	O00762
UBE2S	Q16763	VRK1	Q99986	ZWINT	O95229

21. Mitotic Anaphase (R-HSA-68882)

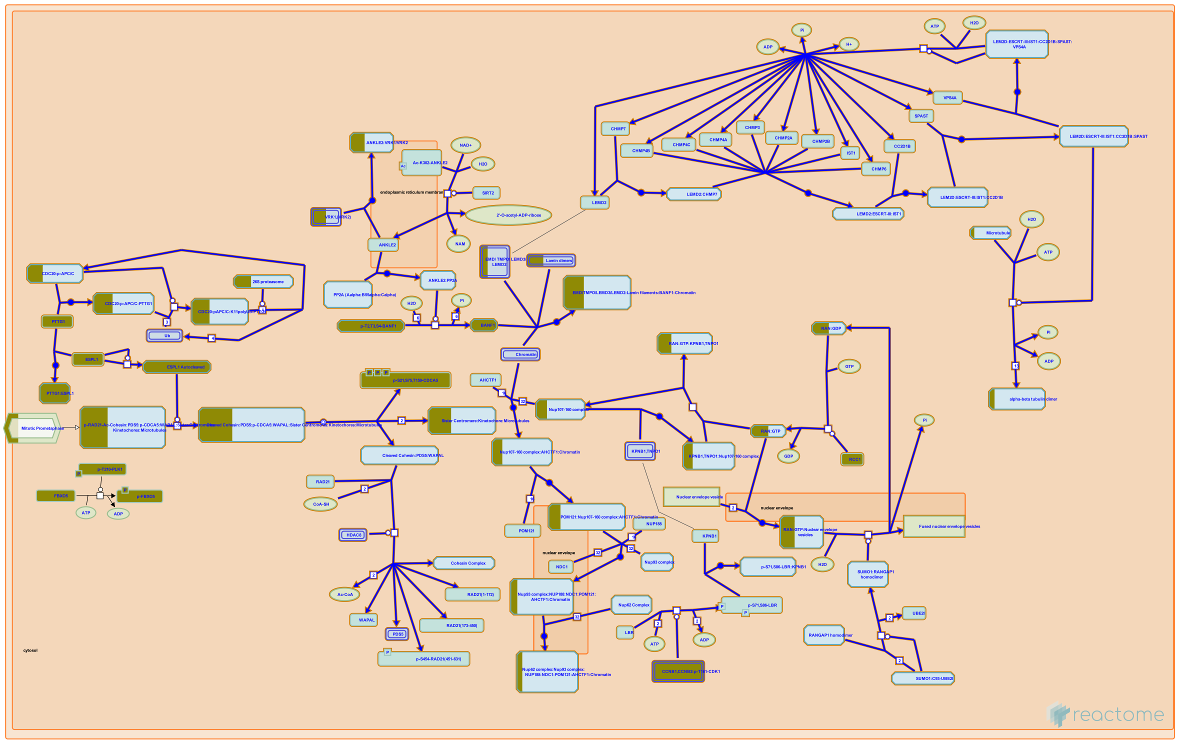

In anaphase, the paired chromosomes separate at the centromeres, and move to the opposite sides of the cell. The movement of the chromosomes is facilitated by a combination of kinetochore movement along the spindle microtubules and through the physical interaction of polar microtubules.

References

Edit history

Date	Action	Author
2021-05-22	Modified	Shorser S

Entities found in this pathway (68)

Input	UniProt Id	Input	UniProt Id	Input	UniProt Id
ANAPC11	Q9NYG5	ANAPC15	P60006	AURKA	Q96GD4
AURKB	Q96GD4	BANF1	O75531	BIRC5	O15392
BUB1	O43683, O60566	BUB1B	O60566	CCNB1	P14635
CCNB2	O95067	CDC20	Q12834	CDCA5	Q96FF9
CDCA8	Q53HL2	CDK1	P06493	CENPA	P49450
CENPE	Q02224	CENPF	P49454	CENPH	Q9H3R5
CENPK	Q9BS16	CENPL	Q8N0S6	CENPM	Q9NSP4
CENPN	Q96H22	CENPO	Q9BU64	CENPU	Q71F23
DSN1	Q9H410	ERCC6L	Q2NKX8	ESPL1	Q14674
INCENP	Q9NQS7	KIF18A	Q8NI77	KIF2C	Q99661
LMNB1	P20700	MAD2L1	Q13257	NDC80	O14777
NUDC	Q9Y266	NUF2	Q9BZD4	NUP37	Q8NFH4
NUP85	Q9BW27	PLK1	P53350	PSMA2	P25787
PSMA4	P25789	PSMA6	P60900	PSMB3	P49720

Input	UniProt Id
PSMB4	P28070
PSMC3	P17980
PSMD12	O00232
RAN	P62826
RCC2	Q9P258
SKA1	Q96BD8
SPC25	Q9HBM1
TUBA1C	Q9BQE3
VRK1	Q99986

Input	UniProt Id
PSMB7	P40306, Q99436
PSMC4	P43686
PSMD3	O43242
RANBP2	P49792
SGO1	Q5FBB7
SKA2	Q8WVK7
TMPO	P42167-1
UBE2C	O00762
ZWINT	O95229

Input	UniProt Id
PSMC1	P62191
PSMC5	P62195
PTTG1	O95997
RCC1	P18754
SGO2	Q562F6
SPC24	Q8NBT2
TUBA1B	P68363
UBE2S	Q16763

22. EML4 and NUDC in mitotic spindle formation (R-HSA-9648025)

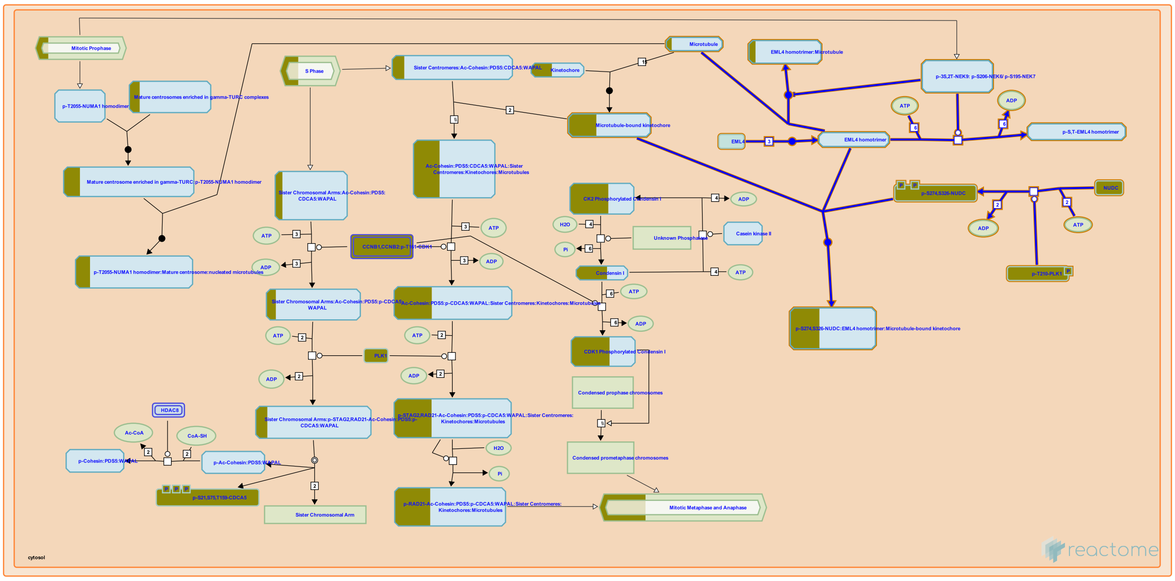

EML4 and NUDC proteins are required for mitotic spindle formation, attachment of spindle microtubule ends to kinetochores, and alignment of mitotic chromosome at the metaphase plate. EML4 is a WD40 family protein that binds to interphase microtubules and stabilizes them (Houtman et al. 2007, Adib et al. 2019). At mitotic entry, EML4 undergoes phosphorylation (Pollmann et al. 2006, Adib et al. 2019) by serine/threonine kinases NEK6 and NEK7, leading to its dissociation from microtubules, which is necessary for the assembly of a dynamic mitotic spindle (Adib et al. 2019). EML4, through its WD40 repeats, interacts with NUDC and recruits it to the kinetochores of the mitotic spindle (Chen et al. 2015). It is possible that other mitotic kinases, besides NEK6 and NEK7, also phosphorylate EML4. Phosphorylation of different residues of EML4 could reduce or increase affinity of EML4 for specific subpopulations of microtubules in mitosis.

A recurrent genomic rearrangement, reported in about 5% cases of non-small cell lung cancer (NSCLC) fuses the N-terminal portion of EML4 with the C-terminal portion of ALK (anaplastic lymphoma kinase), resulting in a constitutively active ALK (Soda et al. 2007, Richards et al. 2015).

References

Chen D, Ito S, Yuan H, Hyodo T, Kadomatsu K, Hamaguchi M & Senga T (2015). EML4 promotes the loading of NUDC to the spindle for mitotic progression. Cell Cycle, 14, 1529-39.

Pollmann M, Parwaresch R, Adam-Klages S, Kruse ML, Buck F & Heidebrecht HJ (2006). Human EML4, a novel member of the EMAP family, is essential for microtubule formation. Exp. Cell Res., 312, 3241-51.

Richards MW, O'Regan L, Roth D, Montgomery JM, Straube A, Fry AM & Bayliss R (2015). Microtubule association of EML proteins and the EML4-ALK variant 3 oncoprotein require an N-terminal trimerization domain. Biochem. J., 467, 529-36.

Soda M, Choi YL, Enomoto M, Takada S, Yamashita Y, Ishikawa S, ... Mano H (2007). Identification of the transforming EML4-ALK fusion gene in non-small-cell lung cancer. Nature, 448, 561-6.

Adib R, Montgomery JM, Atherton J, O'Regan L, Richards MW, Straatman KR, ... Fry AM (2019). Mitotic phosphorylation by NEK6 and NEK7 reduces the microtubule affinity of EML4 to promote chromosome congression. Sci Signal, 12.

Edit history

Date	Action	Author
2019-06-04	Created	Orlic-Milacic M
2019-06-25	Authored	Orlic-Milacic M
2019-09-30	Reviewed	O'Regan L, Fry AM, Lucken KJ
2019-10-03	Reviewed	Bechstedt S
2019-10-07	Edited	Orlic-Milacic M
2021-05-22	Modified	Shorser S

Entities found in this pathway (40)

Input	UniProt Id	Input	UniProt Id	Input	UniProt Id
AURKA	Q96GD4	AURKB	Q96GD4	BIRC5	O15392
BUB1	O43683, O60566	BUB1B	O60566	CDC20	Q12834
CDCA8	Q53HL2	CENPA	P49450	CENPE	Q02224
CENPF	P49454	CENPH	Q9H3R5	CENPK	Q9BS16
CENPL	Q8N0S6	CENPM	Q9NSP4	CENPN	Q96H22
CENPO	Q9BU64	CENPU	Q71F23	DSN1	Q9H410
ERCC6L	Q2NKX8	INCENP	Q9NQS7	KIF18A	Q8NI77
KIF2C	Q99661	MAD2L1	Q13257	NDC80	O14777
NUDC	Q9Y266	NUF2	Q9BZD4	NUP37	Q8NFH4
NUP85	Q9BW27	PLK1	P53350	RANBP2	P49792
RCC2	Q9P258	SGO1	Q5FBB7	SGO2	Q562F6
SKA1	Q96BD8	SKA2	Q8WVK7	SPC24	Q8NBT2
SPC25	Q9HBM1	TUBA1B	P68363	TUBA1C	Q9BQE3
ZWINT	O95229				

23. RHO GTPases Activate Formins (R-HSA-5663220)

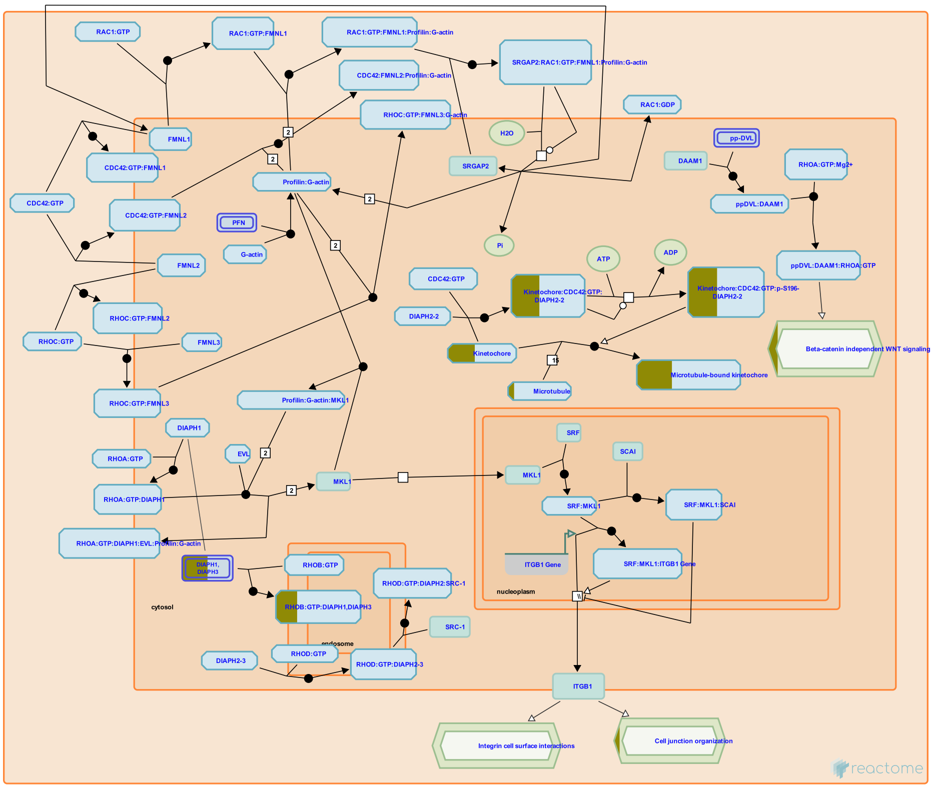

Cellular compartments: cytosol, endosome membrane, nucleoplasm, plasma membrane.

Formins are a family of proteins with 15 members in mammals, organized into 8 subfamilies. Formins are involved in the regulation of actin cytoskeleton. Many but not all formin family members are activated by RHO GTPases. Formins that serve as effectors of RHO GTPases belong to different formin subfamilies but they all share a structural similarity to Drosophila protein diaphanous and are hence named diaphanous-related formins (DRFs).

DRFs activated by RHO GTPases contain a GTPase binding domain (GBD) at their N-terminus, followed by formin homology domains 3, 1, and 2 (FH3, FH1, FH2) and a diaphanous autoregulatory domain (DAD) at the C-terminus. Most DRFs contain a dimerization domain (DD) and a coiled-coil region (CC) in between FH3 and FH1 domains (reviewed by Kuhn and Geyer 2014). RHO GTPase-activated DRFs are autoinhibited through the interaction between FH3 and DAD which is disrupted upon binding to an active RHO GTPase (Li and Higgs 2003, Lammers et al. 2005, Nezami et al. 2006). Since formins dimerize, it is not clear whether the FH3-DAD interaction is intra- or intermolecular. FH2 domain is responsible for binding to the F-actin and contributes to the formation of head-to-tail formin dimers (Xu et al. 2004). The proline-rich FH1 domain interacts with the actin-binding proteins profilins, thereby facilitating actin recruitment to formins and accelerating actin polymerization (Romero et al. 2004, Kovar et al. 2006).

Different formins are activated by different RHO GTPases in different cell contexts. FMNL1 (formin-like protein 1) is activated by binding to the RAC1:GTP and is involved in the formation of lamellipodia in macrophages (Yayoshi-Yamamoto et al. 2000) and is involved in the regulation of the Golgi complex structure (Colon-Franco et al. 2011). Activation of FMNL1 by CDC42:GTP contributes to the formation of the phagocytic cup (Seth et al. 2006). Activation of FMNL2 (formin-like protein 2) and FMNL3 (formin-like protein 3) by RHOC:GTP is involved in cancer cell motility and invasiveness (Kitzing et al. 2010, Vega et al. 2011). DIAPH1, activated by RHOA:GTP, promotes elongation of actin filaments and activation of SRF-mediated transcription which is inhibited by unpolymerized actin (Miralles et al. 2003). RHOF-mediated activation of DIAPH1 is implicated in formation of stress fibers (Fan et al. 2010). Activation of DIAPH1 and DIAPH3 by RHOB:GTP leads to actin coat formation around endosomes and regulates endosome motility and trafficking (Fernandez-Borja et al. 2005, Wallar et al. 2007). Endosome trafficking is also regulated by DIAPH2 transcription isoform 3 (DIAPH2-3) which, upon activation by RHOD:GTP, recruits SRC kinase to endosomes (Tominaga et al. 2000, Gasman et al. 2003). DIAPH2 transcription isoform 2 (DIAPH2-2) is involved in mitosis where, upon being activated by CDC42:GTP, it facilitates the capture of astral microtubules by kinetochores (Yasuda et al. 2004, Cheng et al. 2011). DIAPH2 is implicated in ovarian maintenance and premature ovarian failure (Bione et al. 1998). DAAM1, activated by RHOA:GTP, is involved in linking WNT signaling to cytoskeleton reorganization (Habas et al. 2001).

References

Kühn S & Geyer M (2014). Formins as effector proteins of Rho GTPases. Small GTPases, 5, e29513.

Li F & Higgs HN (2003). The mouse Formin mDia1 is a potent actin nucleation factor regulated by autoinhibition. Curr. Biol., 13, 1335-40.

Lammers M, Rose R, Scrima A & Wittinghofer A (2005). The regulation of mDia1 by autoinhibition and its release by Rho*GTP. EMBO J., 24, 4176-87.

Nezami AG, Poy F & Eck MJ (2006). Structure of the autoinhibitory switch in formin mDia1. Structure, 14, 257-63.

Xu Y, Moseley JB, Sagot I, Poy F, Pellman D, Goode BL & Eck MJ (2004). Crystal structures of a Formin Homology-2 domain reveal a tethered dimer architecture. Cell, 116, 711-23.

Edit history

Date	Action	Author
2014-10-24	Authored	Orlic-Milacic M
2014-12-26	Authored	Rivero Crespo F
2015-01-17	Created	Orlic-Milacic M
2015-02-02	Edited	Orlic-Milacic M
2021-05-22	Modified	Shorser S

Entities found in this pathway (41)

Input	UniProt Id	Input	UniProt Id	Input	UniProt Id
AURKA	Q96GD4	AURKB	Q96GD4	BIRC5	O15392
BUB1	O43683, O60566	BUB1B	O60566	CDC20	Q12834

Input	UniProt Id	Input	UniProt Id	Input	UniProt Id
CDCA8	Q53HL2	CENPA	P49450	CENPE	Q02224
CENPF	P49454	CENPH	Q9H3R5	CENPK	Q9BS16
CENPL	Q8N0S6	CENPM	Q9NSP4	CENPN	Q96H22
CENPO	Q9BU64	CENPU	Q71F23	DIAPH3	Q9NSV4
DSN1	Q9H410	ERCC6L	Q2NKX8	INCENP	Q9NQS7
KIF18A	Q8NI77	KIF2C	Q99661	MAD2L1	Q13257
NDC80	O14777	NUDC	Q9Y266	NUF2	Q9BZD4
NUP37	Q8NFH4	NUP85	Q9BW27	PLK1	P53350
RANBP2	P49792	RCC2	Q9P258	SGO1	Q5FBB7
SGO2	Q562F6	SKA1	Q96BD8	SKA2	Q8WVK7
SPC24	Q8NBT2	SPC25	Q9HBM1	TUBA1B	P68363
TUBA1C	Q9BQE3	ZWINT	O95229		

24. Mitotic G2-G2/M phases (R-HSA-453274)

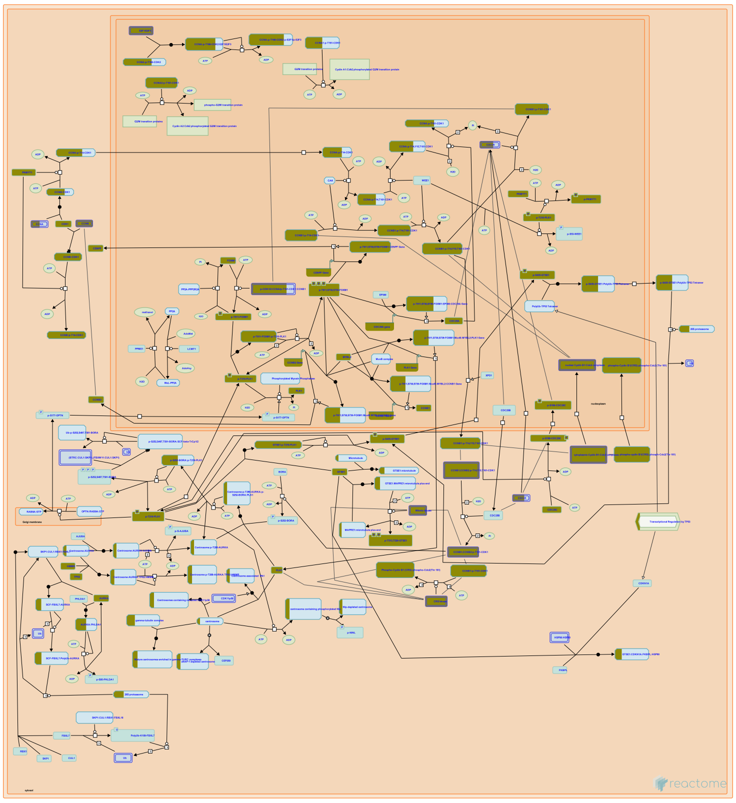

Mitotic G2 (gap 2) phase is the second growth phase during eukaryotic mitotic cell cycle. G2 encompasses the interval between the completion of DNA synthesis and the beginning of mitosis. During G2, the cytoplasmic content of the cell increases. At G2/M transition, duplicated centrosomes mature and separate and CDK1:cyclin B complexes become active, setting the stage for spindle assembly and chromosome condensation that occur in the prophase of mitosis (O'Farrell 2001, Bruinsma et al. 2012, Jiang et al. 2014).

References

Wang G, Jiang Q & Zhang C (2014). The role of mitotic kinases in coupling the centrosome cycle with the assembly of the mitotic spindle. J. Cell. Sci., 127, 4111-22.

O'Farrell PH (2001). Triggering the all-or-nothing switch into mitosis. Trends Cell Biol., 11, 512-9.

Bruinsma W, Raaijmakers JA & Medema RH (2012). Switching Polo-like kinase-1 on and off in time and space. Trends Biochem. Sci., 37, 534-42.

Edit history

Date	Action	Author
2010-01-20	Created	Matthews L
2017-03-24	Edited	Orlic-Milacic M
2018-07-10	Reviewed	Manfredi JJ
2021-05-22	Modified	Shorser S

Entities found in this pathway (39)

Input	UniProt Id	Input	UniProt Id	Input	UniProt Id
ACTR1A	P61163	AURKA	O14965	AURKB	O14965
CCNA2	P20248	CCNB1	P14635	CCNB2	O95067
CDC25A	P30304	CDC25C	P30307	CDK1	P06493, P24941
CDK2	P24941	CENPF	P49454	CEP78	Q5JTW2
E2F1	O00716, Q01094	FOXM1	Q08050	GTSE1	Q9NYZ3
HAUS8	Q9BT25	HMMR	O75330	MYBL2	P10244
MZT2B	Q6NZ67	NEK2	P51955	PKMYT1	Q99640
PLK1	P53350	PLK4	O00444	PSMA2	P25787
PSMA4	P25789	PSMA6	P60900	PSMB3	P49720
PSMB4	P28070	PSMB7	P40306, Q99436	PSMC1	P62191
PSMC3	P17980	PSMC4	P43686	PSMC5	P62195
PSMD12	O00232	PSMD3	O43242	TPX2	Q9ULW0
TUBA1B	P68363	TUBA1C	Q9BQE3	TUBG1	P23258, Q9NRH3

Input	Ensembl Id	Input	Ensembl Id	Input	Ensembl Id
CCNB1	ENSG00000134057	CCNB2	ENSG00000157456	CDC25A	ENSG00000164045
CENPF	ENSG00000117724	PLK1	ENSG00000166851		

25. RHO GTPase Effectors (R-HSA-195258)

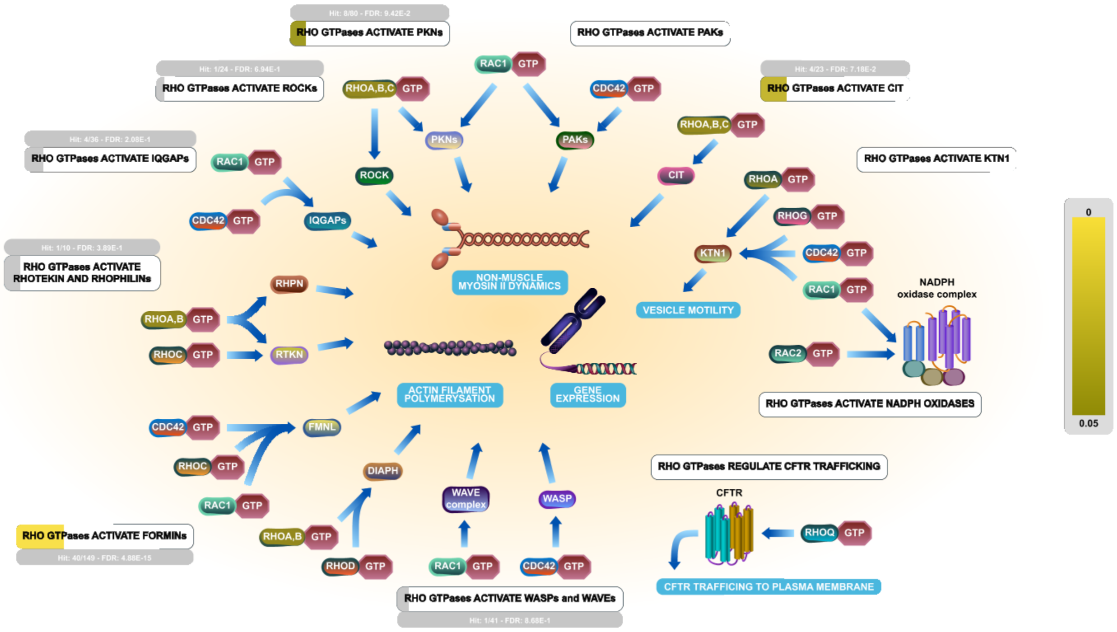

RHO GTPases regulate cell behaviour by activating a number of downstream effectors that regulate cytoskeletal organization, intracellular trafficking and transcription (reviewed by Sahai and Marshall 2002).

One of the best studied RHO GTPase effectors are protein kinases ROCK1 and ROCK2, which are activated by binding RHOA, RHOB or RHOC. ROCK1 and ROCK2 phosphorylate many proteins involved in the stabilization of actin filaments and generation of actin-myosin contractile force, such as LIM kinases and myosin regulatory light chains (MRLC) (Amano et al. 1996, Ishizaki et al. 1996, Leung et al. 1996, Ohashi et al. 2000, Sumi et al. 2001, Riento and Ridley 2003, Watanabe et al. 2007).

PAK1, PAK2 and PAK3, members of the p21-activated kinase family, are activated by binding to RHO GTPases RAC1 and CDC42 and subsequent autophosphorylation and are involved in cytoskeleton regulation (Manser et al. 1994, Manser et al. 1995, Zhang et al. 1998, Edwards et al. 1999, Lei et al. 2000, Parrini et al. 2002; reviewed by Daniels and Bokoch 1999, Szczepanowska 2009).

RHOA, RHOB, RHOC and RAC1 activate protein kinase C related kinases (PKNs) PKN1, PKN2 and PKN3 (Maesaki et al. 1999, Zong et al. 1999, Owen et al. 2003, Modha et al. 2008, Hutchinson et al. 2011, Hutchinson et al. 2013), bringing them in proximity to the PIP3-activated PDPK1 (PDK1) and thus enabling PDPK1-mediated phosphorylation of PKN1, PKN2 and PKN3 (Flynn et al. 2000, Torbett et al. 2003). PKNs play important roles in cytoskeleton organization (Hamaguchi et al. 2000), regulation of cell cycle (Misaki et al. 2001), receptor trafficking (Metzger et al. 2003) and apoptosis (Takahashi et al. 1998). PKN1 is also involved in the ligand-dependent transcriptional activation by the androgen receptor (Metzger et al. 2003, Metzger et al. 2005, Metzger et al. 2008).

Citron kinase (CIT) binds RHO GTPases RHOA, RHOB, RHOC and RAC1 (Madaule et al. 1995), but the mechanism of CIT activation by GTP-bound RHO GTPases has not been elucidated. CIT and RHOA are implicated to act together in Golgi apparatus organization through regulation of the actin cytoskeleton (Camera et al. 2003). CIT is also involved in the regulation of cytokinesis through its interaction with KIF14 (Gruneberg et al. 2006, Bassi et al. 2013, Watanabe et al. 2013).

RHOA, RHOG, RAC1 and CDC42 bind kinectin (KTN1), a kinesin anchor protein involved in kinesin-mediated vesicle motility (Vignal et al. 2001, Hotta et al. 1996). The effect of RHOG activity on cellular morphology, exhibited in the formation of microtubule-dependent cellular protrusions, depends both on RHOG interaction with KTN1, as well as on the kinesin activity (Vignal et al. 2001). RHOG and KTN1 also cooperate in microtubule-dependent lysosomal transport (Vignal et al. 2001).

IQGAP proteins IQGAP1, IQGAP2 and IQGAP3, bind RAC1 and CDC42 and stabilize them in their GTP-bound state (Kuroda et al. 1996, Swart-Mataraza et al. 2002, Wang et al. 2007). IQGAPs bind F-actin filaments and modulate cell shape and motility through regulation of G-actin/F-actin equilibrium (Brill et al. 1996, Fukata et al. 1997, Bashour et al. 1997, Wang et al. 2007, Pelikan-Conchaudron et al. 2011). Binding of IQGAPs to F-actin is inhibited by calmodulin (Bashour et al. 1997, Pelikan-Conchaudron et al. 2011). IQGAP1 is involved in the regulation of adherens junctions through its interaction with E-cadherin (CDH1) and catenins (CTTNB1 and CTTNA1) (Kuroda et al. 1998, Hage et al. 2009). IQGAP1 contributes to cell polarity and lamellipodia formation through its interaction with microtubules (Fukata et al. 2002, Suzuki and Takahashi 2008).

RHOQ (TC10) regulates the trafficking of CFTR (cystic fibrosis transmembrane conductance regulator) by binding to the Golgi-associated protein GOPC (also known as PIST, FIG and CAL). In the absence of RHOQ, GOPC bound to CFTR directs CFTR for lysosomal degradation, while GTP-bound RHOQ directs GOPC:CFTR complex to the plasma membrane, thereby rescuing CFTR (Neudauer et al. 2001, Cheng et al. 2005).

RAC1 and CDC42 activate WASP and WAVE proteins, members of the Wiskott-Aldrich Syndrome protein family. WASPs and WAVEs simultaneously interact with G-actin and the actin-related ARP2/3 complex, acting as nucleation promoting factors in actin polymerization (reviewed by Lane et al. 2014).

RHOA, RHOB, RHOC, RAC1 and CDC42 activate a subset of formin family members. Once activated, formins bind G-actin and the actin-bound profilins and accelerate actin polymerization, while some formins also interact with microtubules. Formin-mediated cytoskeletal reorganization plays important roles in cell motility, organelle trafficking and mitosis (reviewed by Kuhn and Geyer 2014).

Rhotekin (RTKN) and rhophilins (RHPN1 and RHPN2) are effectors of RHOA, RHOB and RHOC and have not been studied in detail. They regulate the organization of the actin cytoskeleton and are implicated in the establishment of cell polarity, cell motility and possibly endosome trafficking (Sudo et al. 2006, Watanabe et al. 1996, Fujita et al. 2000, Peck et al. 2002, Mircescu et al. 2002). Similar to formins (Miralles et al. 2003), cytoskeletal changes triggered by RTKN activation may lead to stimulation of SRF-mediated transcription (Reynaud et al. 2000).

RHO GTPases RAC1 and RAC2 are needed for activation of NADPH oxidase complexes 1, 2 and 3 (NOX1, NOX2 and NOX3), membrane associated enzymatic complexes that use NADPH as an electron donor to reduce oxygen and produce superoxide (O2-). Superoxide serves as a secondary messenger and also directly contributes to the microbicidal activity of neutrophils (Knaus et al. 1991, Roberts et al. 1999, Kim and Dinauer 2001, Jyoti et al. 2014, Cheng et al. 2006, Miyano et al. 2006, Ueyama et al. 2006).

References

Sahai E & Marshall CJ (2002). RHO-GTPases and cancer. Nat. Rev. Cancer, 2, 133-42.

Amano M, Ito M, Kimura K, Fukata Y, Chihara K, Nakano T, ... Kaibuchi K (1996). Phosphorylation and activation of myosin by Rho-associated kinase (Rho-kinase). J Biol Chem, 271, 20246-9.

Ishizaki T, Maekawa M, Fujisawa K, Okawa K, Iwamatsu A, Fujita A, ... Narumiya S (1996). The small GTP-binding protein Rho binds to and activates a 160 kDa Ser/Thr protein kinase homologous to myotonic dystrophy kinase. EMBO J, 15, 1885-93.

Leung T, Chen XQ, Manser E & Lim L (1996). The p160 RhoA-binding kinase ROK alpha is a member of a kinase family and is involved in the reorganization of the cytoskeleton. Mol. Cell. Biol., 16, 5313-27.

Ohashi K, Nagata K, Maekawa M, Ishizaki T, Narumiya S & Mizuno K (2000). Rho-associated kinase ROCK activates LIM-kinase 1 by phosphorylation at threonine 508 within the activation loop. J Biol Chem, 275, 3577-82.

Edit history

Date	Action	Author
2007-04-03	Created	Gopinathrao G
2014-10-24	Authored	Orlic-Milacic M
2014-12-26	Authored	Rivero Crespo F
2015-02-02	Edited	Orlic-Milacic M
2021-05-22	Modified	Shorser S

Entities found in this pathway (55)

Input	UniProt Id	Input	UniProt Id	Input	UniProt Id
AURKA	Q96GD4	AURKB	Q96GD4	BIRC5	O15392
BUB1	O43683, O60566	BUB1B	O60566	CDC20	Q12834
CDC25C	P30307	CDCA8	Q53HL2	CENPA	P49450
CENPE	Q02224	CENPF	P49454	CENPH	Q9H3R5
CENPK	Q9BS16	CENPL	Q8N0S6	CENPM	Q9NSP4
CENPN	Q96H22	CENPO	Q9BU64	CENPU	Q71F23
CFL1	P23528	CIT	O14578, O14578-3	DIAPH3	Q9NSV4
DSN1	Q9H410	ERCC6L	Q2NKX8	H2AFX	P16104
H2AFZ	P0C0S5	HIST1H2AJ	Q99878	HIST1H2BH	P62807, Q93079
HIST1H3F	P68431	HIST2H2AC	Q16777	INCENP	Q9NQS7
IQGAP3	P46940, Q86VI3	KIF14	Q15058	KIF18A	Q8NI77
KIF2C	Q99661	MAD2L1	Q13257	NCKAP1	Q9Y2A7
NDC80	O14777	NUDC	Q9Y266	NUF2	Q9BZD4
NUP37	Q8NFH4	NUP85	Q9BW27	PLK1	P53350
PRC1	O43663	RANBP2	P49792	RCC2	Q9P258
RHPN1	Q8TCX5	SGO1	Q5FBB7	SGO2	Q562F6
SKA1	Q96BD8	SKA2	Q8WVK7	SPC24	Q8NBT2
SPC25	Q9HBM1	TUBA1B	P68363	TUBA1C	Q9BQE3
ZWINT	O95229				

6. Identifiers found

Below is a list of the input identifiers that have been found or mapped to an equivalent element in Reactome, classified by resource.

Entities (436)

Input	UniProt Id	Input	UniProt Id	Input	UniProt Id
ACTR1A	P61163	ADAM10	O14672	ADCY7	P51828
AIP	Q9NWT8	AKAP13	Q12802	ALYREF	Q86V81
ANAPC11	Q9NYG5	ANAPC15	P60006	ANK3	Q12955
ANLN	Q9NQW6	APH1B	Q8WW43	APOBEC3B	Q6NTF7, Q9UH17
ARHGAP11A	Q6P4F7	ARHGAP12	Q8IWW6	ARHGEF39	Q8N4T4
ASF1B	Q9Y294	ASPA	P45381	ATP5MC1	P05496, P48201, Q06055
ATP5ME	P56385	ATP5PD	O75947	ATP5PF	P18859
AURKA	Q96GD4	AURKAIP1	Q9NWT8	AURKB	Q96GD4
BANF1	O75531	BIRC5	O15392	BLM	P54132
BOLA2	Q9H3K6	BRIP1	Q9BX63	BUB1	O43683, O60566
BUB1B	O60566	C1QBP	Q07021	CBX8	Q9HC52
CCDC59	Q9P031	CCNA2	P20248	CCNB1	P14635
CCNB2	O95067	CCNE1	P24864	CCNE2	O96020
CCNF	P41002	CCT4	P50991	CCT7	Q99832
CD3D	P04234, P09693	CDC20	Q12834	CDC25A	P30304
CDC25C	P30307	CDC45	O75419	CDC6	Q99741
CDCA5	Q96FF9	CDCA8	Q53HL2	CDH6	P55285, Q9ULB4
CDK1	P06493	CDK2	P24941	CDK4	P11802
CDKN2D	P55273	CDT1	Q9H211	CENPA	P49450
CENPE	Q02224	CENPF	P49454	CENPH	Q9H3R5
CENPK	Q9BS16	CENPL	Q8N0S6	CENPM	Q9NSP4
CENPN	Q96H22	CENPO	Q9BU64	CENPU	Q71F23
CENPW	Q5EE01	CENPX	A8MT69	CEP78	Q5JTW2
CFL1	P23528	CHAC2	Q8WUX2	CHCHD1	Q96BP2
CHCHD2	Q9Y6H1	CHEK1	O14757	CHEK2	O96017
CIT	O14578, O14578-3	CKS1B	P61024	CNIH2	Q6PI25
COL4A3BP	Q9Y5P4-2	COLGALT1	Q8IYK4, Q8NBJ5	COMMD4	Q9H0A8
COPRS	Q9NQ92	COX5A	P20674	COX6A1	P12074
CPSF3	Q9UKF6	CREBRF	Q8IUR6	CSK	P41240
CSTF3	Q12996	DBF4	Q9UBU7	DCTPP1	Q9H773
DCUN1D5	Q9BTE7	DCXR	Q7Z4W1	DDX39A	O00148
DEPDC1B	Q8WUY9	DIAPH3	Q9NSV4	DLGAP5	Q15398
DNAJC7	Q99615	DNAJC8	O75937	DRAP1	Q14919
DRG1	Q92597	DSCC1	Q9BVC3	DSN1	Q9H410
DTL	Q9NZJ0	DTYMK	P23919	DUT	P33316-2
E2F1	Q01094	E2F2	Q16254	E2F7	Q96AV8
E2F8	A0AVK6	EBP	Q15125	ECT2	Q9H8V3
EIF4A3	P38919	EME1	A4GXA9, Q96AY2	EPC1	Q9H2F5
ERAL1	O75616	ERBIN	Q96RT1	ERCC6L	Q2NKX8

Input	UniProt Id	Input	UniProt Id	Input	UniProt Id
ERN1	O75460	ESCO2	Q56NI9	ESPL1	Q14674
EXO1	Q9UQ84	EXOSC2	Q13868	EXOSC7	Q15024
EZH2	Q15910	FANCA	O15360	FANCB	Q8NB91
FANCD2	Q9BXW9	FANCG	O15287	FANCI	Q9NVI1
FBXO5	Q9UKT4	FEN1	P39748	FNIP2	Q9P278
FOXM1	Q08050	GAB2	Q9UQC2	GADD45GIP1	Q8TAE8
GCC2	Q8IWJ2	GEMIN6	Q8WXD5	GINS1	Q14691
GINS2	Q9Y248	GINS3	Q9BRX5	GINS4	Q9BRT9
GMFG	O60234	GMNN	O75496	GNA15	P30679
GOLGA2	Q08379	GPS1	Q13098	GRK6	P43250
GTF3A	Q92664	GTSE1	Q9NYZ3	H2AFX	P16104
H2AFZ	P0C0S5	HAUS8	Q9BT25	HIST1H2AJ	Q99878
HIST1H2BH	P62807, Q93079	HIST1H3C	Q71DI3	HIST1H3F	P68431
HIST2H2AC	Q16777	HJURP	Q8NCD3	HMGB2	P26583
HMMR	O75330	HNRNPA1	P09651	HNRNPC	P07910
HNRNPD	Q14103	HNRNPL	P14866	IDS	P22304
IL32	P24001	INCENP	Q9NQS7	IQGAP3	P46940, Q86VI3
IRS1	P35568	KAT2B	Q92831	KCNIP4	Q6PIL6
KIF11	P52732	KIF14	Q15058	KIF15	Q9NS87
KIF18A	Q8NI77	KIF18B	Q86Y91	KIF20A	O95235
KIF22	Q14807	KIF23	Q02241	KIF27	Q86VH2
KIF2C	Q99661	KIF4A	O95239, Q2VIQ3	KIF4B	O95239, Q2VIQ3
KIFC1	Q9BW19	KPNA2	P52292	KPTN	Q9Y664
LGR4	Q9BXB1	LIMS1	P48059	LMBRD1	Q9NUN5
LMNB1	P20700	LRR1	Q96L50	LSM2	Q9Y333
LSM4	Q9Y4Z0	LSM5	Q9Y4Y9	LSM7	Q9UK45
MAD2L1	Q13257	MAD2L2	Q9UI95	MAGOH	P61326, Q96A72
MAGOHB	P61326, Q96A72	MAML2	Q8IZL2	MAN2A1	Q16706
MAN2B1	O00754	MCM10	Q7L590	MCM2	P33993, P49736
MCM5	P33992	MCM6	Q14566	MCM7	P33993
MEF2D	Q14814	MEMO1	Q9Y316	MICOS10	Q5TGZ0
MIS18A	Q9NYP9	MND1	Q9BWT6	MRM3	Q9HC36
MRPL11	Q9Y3B7	MRPL12	P52815	MRPL20	Q9BYC9
MRPL21	Q7Z2W9	MRPL27	Q8IXM3, Q9P0M9	MRPL38	Q96DV4
MRPL44	Q9H9J2	MRPL47	Q9HD33	MRPL51	Q4U2R6
MRPL52	Q86TS9	MRPL58	Q14197	MRPS12	O15235
MRPS15	P82914	MRPS17	Q9Y2R5	MRPS23	Q9Y3D9
MRPS24	Q96EL2	MRPS26	Q9BYN8	MRPS34	P82930
MRPS5	P82675	MRPS7	Q9Y2R9	MRPS9	P82933
MTMR3	Q96QG7	MYBL2	P10244	MZT2B	Q6NZ67
NARF	Q8WVD3	NCAPD2	Q15021	NCAPG	Q9BPX3
NCAPH	Q15003	NCKAP1	Q9Y2A7	NDC80	O14777
NDUFAB1	O14561	NDUFB11	Q9NX14	NDUFB9	Q9Y6M9
NDUFS5	O43920	NDUFS6	O75380	NEDD8	Q15843
NEIL3	Q8TAT5	NEK2	P51955	NFATC2	Q12968, Q13469
NHP2	Q9NX24	NIPAL2	Q9H841	NME1	O60361, P15531
NME2	O60361, P22392	NOC4L	Q9BVI4	NOL11	Q9H8H0
NOP56	O00567	NR3C2	P08235-1, P08235-2, P08235-3, P08235-4	NT5C3B	Q969T7

Input	UniProt Id	Input	UniProt Id	Input	UniProt Id
NUDC	Q9Y266	NUDT1	P36639-1, P36639-2, P36639-3, P36639-4	NUF2	Q9BZD4
NUP37	Q8NFH4	NUP85	Q9BW27	OIP5	O43482
ORC1	Q13415	ORC6	Q9Y5N6	OSMR	Q99650
P2RX7	Q99572	PA2G4	Q9UQ80	PABPN1	Q86U42
PAGR1	Q9BTK6	PAM16	Q9Y3D7	PCLAF	Q15004
PCYT2	Q99447	PDAP1	Q13442	PFDN4	Q9NQP4
PGP	A6NDG6	PHB	P35232	PHF19	Q5T6S3
PHKB	Q93100	PIF1	Q9H611	PKMYT1	Q99640
PLK1	P53350	PLK4	O00444	PLXNC1	O60486
POC1A	Q8N3Y1	POLA2	Q14181	POLD1	P28340
POLD2	P49005	POLE2	P56282	POLQ	O75417
POLR2D	O15514	POLR2F	P61218	POLR2I	P36954
POLR2J	P52435	POP7	O75817	PPIH	O43447
PPIL1	Q9Y3C6	PPIP5K1	Q6PFW1	PRC1	O43663
PRKAA1	Q13131	PSMA2	P25787	PSMA4	P25789
PSMA6	P60900	PSMB3	P49720	PSMB4	P28070
PSMB7	P40306, Q99436	PSMC1	P62191	PSMC3	P17980
PSMC3IP	Q9P2W1	PSMC4	P43686	PSMC5	P62195
PSMD12	O00232	PSMD3	O43242	PTGES3	Q15185
PTPN6	P29350	PTTG1	O95997	RAC3	P60763
RACGAP1	Q9H0H5	RAD51	Q06609	RAD51AP1	Q96B01
RAD54L	P46100	RALGAPA2	Q2PPJ7	RAN	P62826
RANBP1	P43487	RANBP2	P49792	RASA1	P20936
RASEF	Q7Z6P3	RBL2	Q08999	RCC1	P18754
RCC2	Q9P258	RFC2	P35249, P35250	RFC4	P35249, P35250
RFC5	P40937, P40938	RHPN1	Q8TCX5	RMI2	Q96E14
RPA3	P35244	RPL26L1	Q9UNX3	RPL38	P63173
RPL39L	Q96EH5	RPP21	Q9H633	RPP30	P78346
RRM2	P31350	RTRAF	Q9Y224	RUVBL2	Q9Y230
SAP30BP	Q9UHR5	SARNP	P82979	SERINC5	Q86VE9
SFPQ	P23246	SGMS2	Q8NHU3	SGO1	Q5FBB7
SGO2	Q562F6	SGPP2	Q8IWX5	SKA1	Q96BD8
SKA2	Q8WVK7	SLBP	Q14493	SLC28A3	Q9HAS3
SLC6A6	P31641	SNF8	Q96H20	SNRNP25	Q9BV90
SNRPA	P09012	SNRPA1	P09661	SNRPB	P14678, P63162
SNRPC	P09234	SNRPD1	P62314, P62316	SNRPD2	P62316
SNRPD3	P62318	SNRPE	P62304	SNRPF	P62306
SNRPG	P62308	SPATA13	Q96N96	SPC24	Q8NBT2
SPC25	Q9HBM1	SPRN	Q5BIV9	SRRT	Q9BXP5
SRSF2	Q01130	SRSF3	P84103	SSB	P05455
SSBP1	Q04837	STIP1	P31948	STMN1	P16949
SUMO2	P61956	SUV39H1	O43463	SUV39H2	Q9H5I1
SYCE2	Q6PIF2	TACC3	Q9Y6A5	TACO1	Q9BSH4
TANK	Q92844	TBC1D7	Q9P0N9	TBCE	Q15813
TFDP1	Q14186	TGOLN2	O43493	THOC6	Q86W42
THOP1	P52888	TIMELESS	Q9UNS1	TIMM50	Q3ZCQ8
TIPIN	Q9BVW5	TK1	P04183	TMPO	P42167-1
TOMM22	Q9NS69	TOMM40	O96008	TOP2A	P11388
TPX2	Q9ULW0	TRA2B	P62995	TRAIP	Q9BWF2

Input	UniProt Id	Input	UniProt Id	Input	UniProt Id
TUBA1B	P68363	TUBA1C	Q9BQE3	TUBG1	P23258, Q9NRH3
TUT7	Q5VYS8	TXNL4A	P83876	TYMS	P04818
U2AF1	Q01081	U2AF2	P26368	UBE2C	O00762
UBE2L3	P68036	UBE2S	Q16763	UBE2T	Q9NPD8
UGCG	Q16739	UHRF1	Q96T88	UTP15	Q8TED0
UTP18	Q9Y5J1	UTRN	P46939	VDAC2	P45880
VRK1	Q99986	WDR34	Q96EX3	WDR77	Q9BQA1
WDR83	Q9BRX9	WRAP53	Q9BUR4	ZNF701	Q9NV72
ZNF791	Q3KP31	ZNRD1	Q9P1U0	ZSWIM8	A7E2V4
ZWINT	O95229				

Input	Ensembl Id	Input	Ensembl Id	Input	Ensembl Id
AIP	ENSG00000110711	BIRC5	ENSG00000089685	BOLA2	ENSG00000183336
CCNA2	ENSG00000145386	CCNB1	ENSG00000134057	CCNB2	ENSG00000157456
CCNE1	ENSG00000105173	CDC25A	ENSG00000164045	CDC25C	ENSG00000158402
CDC45	ENSG00000093009	CDC6	ENSG00000094804	CDK1	ENSG00000170312
CDT1	ENSG00000167513	CENPF	ENSG00000117724	CFL1	ENSG00000172757
CHEK1	ENSG00000149554, ENST00000438015	DLGAP5	ENSG00000126787	DRG1	ENSG00000104419
E2F1	ENSG00000101412	E2F7	ENSG00000165891	EZH2	ENSG00000106462
FANCD2	ENSG00000144554, ENST00000419585	FANCI	ENSG00000140525, ENST00000310775	FBXO5	ENSG00000112029
KPNA2	ENSG00000182481, ENST00000330459.7	LMNB1	ENSG00000113368	MYBL2	ENSG00000101057
ORC1	ENSG00000085840	PLK1	ENSG00000166851	PTPN6	ENSG00000111679
RAD51	ENSG00000051180	RBL2	ENSG00000103479	RRM2	ENSG00000171848
SNRPA1	ENSG00000131876	SSBP1	ENSG00000106028	STMN1	ENSG00000117632
TK1	ENSG00000167900	TOP2A	ENSG00000131747	TYMS	ENSG00000176890

7. Identifiers not found

These 193 identifiers were not found neither mapped to any entity in Reactome.

ABHD2	ALKBH4	AMZ2	ARL6IP1	ASPM	ATP5MD	AUNIP	BBOF1
BCL2L12	BCL7C	BOLA3	C12ORF10	C12ORF57	C17ORF53	C17ORF67	C19ORF48
C20ORF27	C4ORF46	C5ORF34	CARF	CARHSP1	CBX7	CCDC124	CCDC137
CCDC138	CCDC150	CCDC167	CCDC58	CDCA2	CDCA3	CDCA4	CDKN3
CENPV	CEP55	CHAF1A	CHAF1B	CIP2A	CISD1	CKAP2L	CKS2
CLMN	CRIM1	DAZAP1	DDIAS	DEPDC1	DNAJC9	DPP8	DUS1L
ERI3	FAM111B	FAM136A	FAM50A	FAM72A	FAM72B	FAM72D	FAM83D
FBXO43	FICD	FKBP3	FRMD4B	FRY	FYCO1	GNB1L	GPN3
HASPIN	HIVEP2	HMGB3	HMGN2	HNRNPAB	HYPK	ILKAP	JPT1
KCTD18	KNSTRN	LBHD1	LCOR	LEPROT	LIMD2	LMNB2	LOC643387
LSM12	MAGI1	MALSU1	MARF1	MAST4	MAZ	MEA1	MELK
METTL23	METTL2A	MFSD2B	MINDY2	MKI67	MROH7	MRTO4	MTFR2
MXD3	MZB1	NAA10	NBEAL1	NCOA4	NDUFAF8	NFAT5	NRM
NUSAP1	PAQR4	PARPBP	PBK	PBXIP1	PDCL3	PIMREG	PLEKHA7
PLEKHM3	PPM1G	PRPSAP1	PRR11	PSRC1	PTMS	PTPN21	PTTG3P
PUSL1	RALY	RAVER1	RBM43	RCCD1	RDM1	RECQL4	RGPD3
RGPD4	RIBC2	RNASEH2A	RNASEH2C	ROMO1	RREB1	RTKN2	SAAL1
SAC3D1	SAPCD2	SCART1	SCNM1	SECISBP2L	SH3BGRL3	SHCBP1	SIVA1
SKA3	SLC25A33	SLC25A39	SLC35B1	SLC35F5	SLC38A7	SLIRP	SMCR8
SNRK	SPAG5	STAC3	STIL	STX12	SUSD6	TBC1D2B	TCF19
TCOF1	TCP11L2	TECPR1	TEDC1	TEDC2	TEFM	THAP3	TICRR
TM9SF2	TMEM160	TMPO-AS1	TMSB15A	TRAF4	TRIP13	TROAP	TSC22D2
TTK	TYMSOS	UBXN4	VPS13C	VPS16	WDR54	WDR62	WDR74
YDJC	ZBTB38	ZMYND10	ZNF367	ZNF444	ZNF695	ZNF827	ZNFX1
ZNHIT3							
